# Packing Preferences of Chalcones: A Model Conjugated Pharmaceutical Scaffold

**DOI:** 10.1021/acs.cgd.1c01381

**Published:** 2022-02-11

**Authors:** Louise S. Price, Sarah L. Price

**Affiliations:** Department of Chemistry, University College London, 20 Gordon Street, London WC1H 0AJ, U.K.

## Abstract

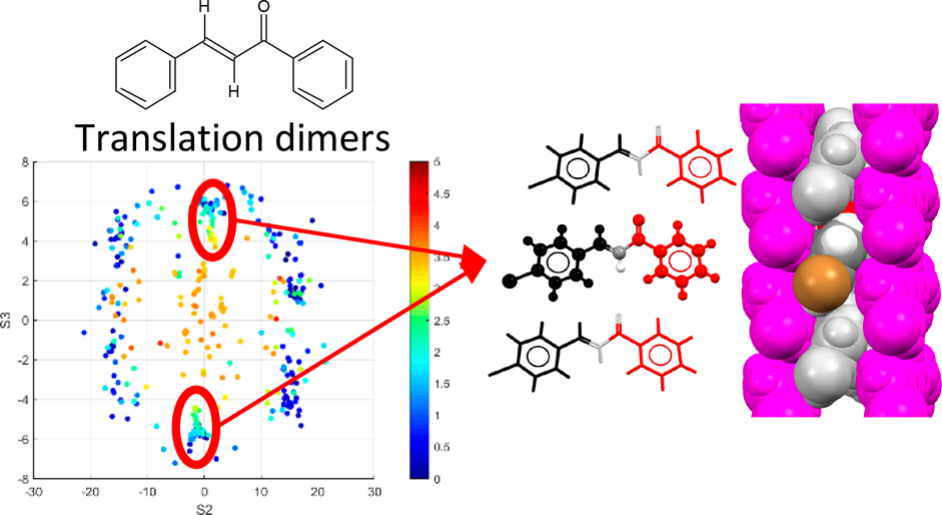

We sought the crystal packing preferences of the chalcone scaffold by analyzing 232 single-component crystal structures of chalcones with a small (six or fewer non-hydrogen atoms) substituent on either or both rings, including the unsubstituted molecule. This covers 216 molecules, as some are polymorphic, and 277 independent molecular conformations, as 16% of the crystal structures have more than one symmetry independent molecule. Quantum mechanical conformational profiles of the unsubstituted molecule and the almost 5000 crystal structures within 20 kJ mol^–1^ of the global minimum generated in a crystal structure prediction (CSP) study have been used to complement this analysis. Although π conjugation would be expected to favor a planar molecule, there are a significant number of crystal structures containing nonplanar molecules with an approximately 50° angle between the aromatic rings. The relative orientations of the molecules in the inversion-related dimers and translation-related dimers in the experimental crystal structures show the same trends as in the CSP-generated structures for the unsubstituted molecule, allowing for the substituent making the side-to-side distances larger. There is no type of dimer geometry associated with particularly favorable lattice energies for the chalcone core. Less than a third of the experimental structures show a face-to-face contact associated with π···π stacking. Analysis of the experimental crystal structures with XPac and Mercury finds various pairs of isostructural crystals, but the largest isostructural set had only 15 structures, with all substituents (mainly halogens) in the para position. The most common one-dimensional motif, found in half of the experimental crystal structures, is a translation-related side-to-side packing, which can be adopted by all the observed conformations. This close-packed motif can be adopted by chalcones with a particularly wide variety of substituents as the substituents are at the periphery. Thus, although the crystal structures of the substituted chalcones show thermodynamically plausible packings of the chalcone scaffold, there is little evidence for any crystal engineering principle of preferred chalcone scaffold packing beyond close packing of the specific molecule.

## Abstract 2

Analysis of 232 crystal structures of chalcones with small substituents and a crystal structure prediction on the unsubstituted chalcone shows that the packing is dominated by the core. Since this core has a wide variety of packings of similar stability, the packing changes necessitated by the substituents produce a wide range of observed structures, with three types of conformations.

## 1 Introduction

The expanding field of crystal engineering aims to aid the design of new functional organic materials through understanding the preferred intermolecular contacts.^1−7^ There are many well-known trends, such as the preference of carboxylic acids to form the *R*_2_^2^(8) hydrogen-bonded dimer,^8^ but this does not define how this dimer packs to form the extended crystal structure, and the preference can be weak, as tetrolic acid shows this motif in one polymorph and a hydrogen-bonded chain in another.^9^ When there are multiple hydrogen-bonding donors and acceptors in a molecule, then Etter’s rule that the strongest donor pairs with the strongest acceptor^10^ is not always reliable. It can be refined by taking into account the bonding environment of the donors and acceptors in calculating the hydrogen-bond propensity^11^ from known crystal structures in the Cambridge Structural Database (CSD).^12^ Exceptions may be understood by looking at whether steric factors prevent the formation of an anticipated hydrogen bond using Full Interaction Maps,^13,14^ again based on CSD data. Hence, even the preferred hydrogen-bond pairing in the crystal structure is not always readily predicted.^11,15,16^ Hydrogen bonding may be in competition with other strong interactions identified with only a few substituent atoms, such as halogen bonding. The competition between hydrogen and halogen bonds^17^ is affected by substituent position^18^ and better rationalized by the electrostatic potential around the molecules (i.e., taking into account the covalent bonding environment within each molecule).^19^ Our knowledge of the competitive strength of different intermolecular interactions identified by small substituents (e.g., -NH_2_, OH, F, Cl, Br, I, C≡N) can be estimated from crystal structures in the CSD, but the data often need supplementing by systematic competitive studies.^20^

The situation is even less clear for aromatic groups. Benzene rings prefer a T-shaped or offset parallel geometry because the electrostatic effects of the π electrons destabilize the simple stacked geometry favored by the dispersion interactions.^21^ However, this preference is readily changed by substituents, e.g., C_6_H_6_/C_6_F_6_ forms columns,^22^ or the introduction of heteroatoms into the π system that results in changes to the σ framework that interact favorably with the π system.^21^ The π···π interactions between two aromatic species are broadly classified by geometry into three categories: edge-to-face T-shape, parallel displaced, and cofacial parallel stacked.^23^ The small, unsubstituted aromatic compounds prefer edge-to-face T-shaped geometry, whereas substituted and large multiring aromatic compounds prefer parallel displaced geometry. Cofacial parallel stacked geometry is rather rarely observed.^23^ However, as many optoelectronic functional materials are based on the stacking of the π systems,^24^ there is huge interest in developing strategies for controlling π···π stacking and its influence on charge transport properties.^25^ The terms π stacking and π···π interactions persist for their relationships to many important properties, despite the arguments that these terms should no longer be used.^26^

Thus, the complex balance of the different types of intermolecular interaction (hydrogen bonding, halogen bonding, π···π, etc.) and the variety of molecular shapes that can be adopted by flexible molecules usually has to be evaluated by computer in a Crystal Structure Prediction (CSP) study.^27−29^ The results are very specific to the molecule but can often show that there are a variety of different packings, often with different hydrogen-bonding motifs and molecular conformations, that are very similar in energy. These competitive structures are different compromises between the geometries of the named atom–atom intermolecular interactions^30^ and the requirement for close packing to optimize the dispersion energy.^31,32^ Indeed, this compromise can result in the first coordination shell including a destabilizing molecule–molecule interaction.^33^

Instead of building up the crystal structure from the interactions between different atoms or small functional groups, it is desirable to consider the packing of a molecular core or scaffold, expecting that molecules that differ only in small substituents should have similarities in their crystal packing. Such a series of molecules may arise from a drug or functional materials discovery program, where the core has desired functionality that can be tuned by varying the substituents. Being able to find isostructural crystal structures of related molecules is often of interest as a route to tunable solid solutions^34^ or templating the first nucleation of a desired polymorph.^35^ There is an increasing number of analyses of large sets of crystal structures of molecules with a large common component, such as the analysis of mandelic acids^36^ and 4,4′ benzenesulfonamidobenzenes.^37^ Such analyses are limited to the members of the series of related molecules that crystallize readily and well enough for structure determination. (Unfortunately there are only few published systematic studies as to the factors which determine whether a series of molecules will crystallize readily, badly, or not at all, for example, a study of acylanilide structures.^38^) The experimental structures of a set of related molecules can be complemented by CSP-generated structures, as has been done to understand the conductivity of chiral [6]-helicene crystals^39^ and the packing preferences of quinoxalines.^40^

**Figure 1:**
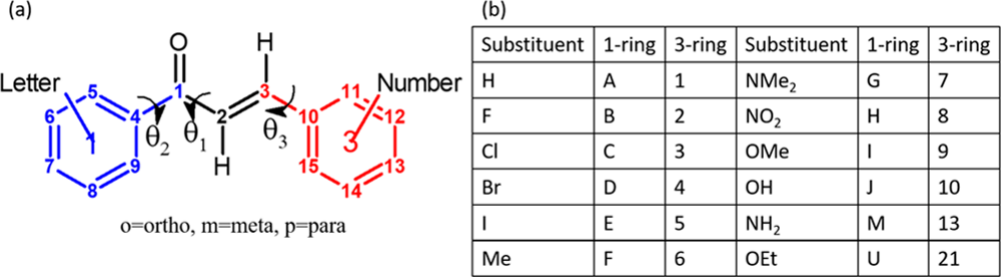
(a) The molecular diagram of the unsubstituted chalcone, with the atomic numbering used in this work. The main torsion angles are marked, with θ_1_ defined by C4–C1–C2=C3, θ_2_ defined by C5–C4–C1–C2, and θ_3_ defined by C2=C3–C10–C11. C5 and C11 are chosen so that θ_2_ and θ_3_ are closer to 180° than 0°. (b) The codes used in this work for the most common substituents. See Table S1 in the Supporting Information for the full list. The systematic naming of molecules studied in this work is XiYj, where X denotes the substituent on the 1-ring as a letter, Y denotes the substituent on the 3-ring as a number, and *i*,*j* denote whether the substituents on the respective rings are in the ortho, meta, or para positions.

One family of molecules that require such analysis is the chalcones ((2*E*)-1,3-diphenylprop-2-en-1-ones, Figure 1). This scaffold is pharmacologically significant^41^ because of the biological activity of chalcones, with some being antioxidants,^42^ cytotoxic, antimicrobial, anticancer,^43,44^ anti-inflammatory,^45^ and antibacterial.^46,47^ Two clinically approved chalcones are Metochalcone, a choleretic drug, and Sofalcone which is both an antiulcer and mucoprotective drug.^48^ Chalcones are used as agrochemicals for the prevention of fungal/insect infestation,^49^ as well as for viral prevention.^50^ It is thought that the main active functional group of these compounds is the α,β unsaturated ketone.^51^ The overlap of the π molecular orbitals of some chalcones leads to a variety of optical properties. Indeed, the name is derived from the Greek word for copper, chalcos, with allusion to the red color of some chalcone derivatives. The bright colors of fruits and vegetables are due in part to the flavonoids, derived from chalcones. Some chalcone derivatives are polymorphic, with different colors exhibited by different polymorphs,^52^ arising from the packing differences. Chalcones are also used as fluorescent probes in imaging, such as a library of dialkyaminochalcones,^53^ many of which showed high fluorescence in DMSO, and a sharp structure–activity relationship in cellular cytotoxicity. A chalcone amide library,^54^ with an amido and an amino on each side of the scaffold as electron donors, was also produced to provide a range of fluorescent probes. Thus, the chalcone chemical scaffold shown in Figure 1 is present in a wide variety of crystal structures in the Cambridge Structural Database. A recent collaboration has generated a significant number of additional crystal structures with small substituents to aid this investigation (Supporting Information, Table S2). Nonetheless, difficulties have been reported in crystallizing chalcones with multiple methoxy substituents^55^ and some chalcones that required laser-assisted crystallization.^56^

The unsubstituted chalcone ((2*E*)-1,3-diphenylprop-2-en-1-one, Figure 1) has only one heteroatom and so cannot form any traditional hydrogen bonds, and only a limited number of C–H···O hydrogen bonds, but the two phenyl rings will give rise to aromatic interactions. The molecule’s two aromatic rings are linked by the enone group and should display some degree of conjugation throughout the molecule, with the fully planar conformation showing the maximum conjugation. Thus, we might expect the crystal structures of chalcones to show π-stacks of planar molecules. However, such stacks are not seen in either of the polymorphs of the unsubstituted chalcone.

In this paper, we have carried out a systematic review of the molecular conformations and crystal structures of a few hundred chalcones with one relatively small substituent (phenyl or smaller) on one or both rings. These analyses are contrasted with the structures for the unsubstituted chalcone generated by a CSP study, to see the extent to which the packing preferences of the core are reflected in the structures of the substituted molecules. The analysis compares first the conformations and then the coordination environments, looking both at pairs of molecules cut from the crystal structure (dimers) and the coordination using the Crystal Packing Similarity tool in Mercury^57^ to establish how many molecules of the 20 molecule cluster can be overlaid and the optimum overlay (RMSD_*n*_). Extended motifs are analyzed using XPac^58^ pairwise comparisons for seeking 1- to 3-D similarity. Crystal Packing Similarity and XPac are used to establish the isostructural families of chalcone crystal structures. This novel combination of complementary analyses and use of CSP could be applied to other families of molecules.

## 2 Experimental Section

### 2.1 Naming Convention

The naming convention for molecules studied in this work is described in Figure 1.

### 2.2 Data Set of Chalcone Experimental Structures

Experimental structures from the 2019 Crystal Structure Database (CSD),^12,59^ including Feb, May, and Aug 2019 updates, and recent structures determined by collaborators, were included in the conformational and structural analysis. A search of the CSD was carried out to find all structures containing the 18 atoms of Figure 1a (i.e., not including ring substituents), where 3-D coordinates were available, discounting any structures containing ions, organometallics, or elements heavier than iodine. This was manually refined to remove zwitterions, multicomponent systems, substituents with more than six non-hydrogen atoms, and molecules with more than one substituent on either ring. This data set was augmented by recently solved structures from our collaborators’ groups. The full criteria for inclusion are given in the Supporting Information, Section S2. A small number of structures were disordered or did not have hydrogen atoms located, and these were edited manually as necessary (see Supporting Information, Section S2), adding hydrogen atoms and removing minor disorder components, to allow automatic analysis of geometries.

### 2.3 CSP Methodology

Conformational analysis of the unsubstituted chalcone backbone was carried out in GAUSSIAN,^60^ at PBE0, B3LYP, MP2, and PBE levels of theory with the 6-31G(d,p) basis set. CrystalPredictor_2.2^61^ was used as the search algorithm. Two separate *Z*′ = 1 searches were carried out, first with a flexible molecule where θ_1_, θ_2_, and θ_3_ were allowed to vary, with θ_1_ around 180° (denoted Region A), and second with a rigid gas phase optimized molecule with θ_1_ close to −30° (denoted Region B). Intramolecular energy and point charges were evaluated at the PBE0/6-31G(d,p) level of theory, and the FIT empirical exp-6 repulsion-dispersion parameters were used.^62,63^ CrystalOptimizer_2.4.7^64^ was used to refine the structures, with DMACRYS_2.3.0.^65^ The intramolecular energy at the PBE0/6-31G(d,p) level of theory was evaluated using GAUSSIAN,^60^ and the distributed multipoles that represent this charge density were evaluated using GDMA_2.2.^66^ The intermolecular lattice energy was evaluated from the distributed multipoles and an empirical repulsion-dispersion potential with the FIT parameters.^62,63^ In addition to the three torsion angles shown in Figure 1, two further torsion angles and six bond angles around the center of the molecule were allowed to change in response to the packing forces as the crystal structure was optimized. Duplicate structures were removed according to similarities in the powder patterns and coordination environment overlay, using RMSD_15_ or RMSD_30_ for structures in the same or different space groups, respectively. Any structures that DMACRYS indicated were high-symmetry saddle points between two lower symmetry structures were reminimized with CrystalOptimizer in the lower symmetry subgroup. Full details of the generation of the CSP crystal structures are in the Supporting Information, Section S5.

### 2.4 Geometric Analyses

The crystal structures generated in the CSP and those in the Experimental Set were inspected using the CCDC Python API on a Linux cluster. This allowed measurement of geometric parameters within the molecules and between pairs of molecules in each crystal structure. The full definition of all parameters measured and the Python code are given in the Supporting Information, Section S7.

#### 2.4.1 Molecular Parameters

The most significant conformational parameters (Figure 1) are θ_1_ (C4–C1–C2=C3), the central torsion angle of the molecule, and the phenyl torsion angles, θ_2_ (C5–C4–C1–C2) and θ_3_ (C2=C3–C10–C11). To give an assessment of the overall planarity of the molecule, the dihedral angle C8–C5–C11–C14 was also measured and is termed planar1.

#### 2.4.2 Intermolecular Parameters

The intermolecular contacts for all pairs of molecules in van der Waals contact were analyzed. The distance between pairs of molecules was measured as C2···C2′ (defined as D1), and we started the analysis with all pairs of molecules whose D1 separation was less than 30 Å for the experimental structures and less than 20 Å for CSP structures. Further geometric parameters were measured (see Supporting Information, Section S7), including an intermolecular torsion angle, C12–C2···C2′–C12′, which was used to define contacts as inversion or translation if this was exactly 180° or 0°, respectively, and these were analyzed in detail.

**Figure 2:**
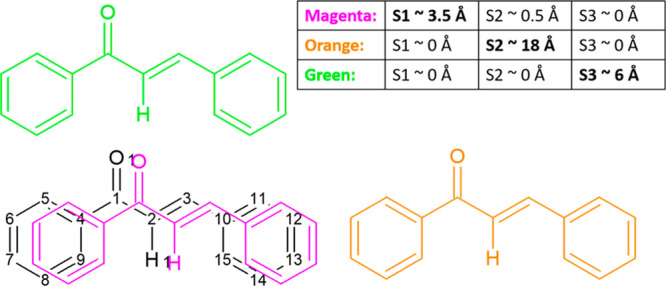
Illustration of the three quantities S1, S2, and S3 for translation-related molecules. For each dimer pair, typical values for S1, S2, and S3 are given for closest approach of the colored molecules with respect to the black molecule.

For each inversion or translation contact, an average plane through each molecule was defined, and the separation of these planes measured as S1. As the C2–H1 and C2–C12 directions and the interplanar separation are approximately orthogonal (see Section S7.3.1 of the Supporting Information for testing of this assumption), the component of D1 parallel to C2–C12 was defined as S2, and the component of D1 parallel to C2–H1 was defined as S3 (Figure 2).

Given the anisotropy of the molecular shape, a “coordination sphere” does not encompass the molecules in van der Waals contact. A “coordination ellipsoid” was defined, and all contacts where  were defined as the first coordination ellipsoid for the structures in the Experimental Set and  for the CSP structures. The values for the denominators were determined by inspecting the full 3-D plots of S1, S2, and S3 and deciding from the absence of structures where the ellipsoids lay. The substituent volumes meant that the maximum values for S1 and S2 are larger for the Experimental Set. Analysis of the conformation-dependent box that contains the molecule^67^ was used to confirm that the maximum values of S1, S2, and S3 chosen for the CSP Set were appropriate (see Supporting Information, Section S6).

### 2.5 Structural Correspondence

The geometries (as S1, S2, and S3) of all inversion and translation van der Waals contact dimers were plotted, to enable a comparison of the entire sets of crystal structures. Where there was a cluster of points from close contacts, the corresponding crystal structures were manually inspected for common dimer packing motifs.

### 2.6 XPac and Mercury Crystal Packing Similarity

Both XPac^58,68^ and Mercury (Crystal Packing Similarity tool)^69^ were used to carry out pairwise analysis of all structures in the Experimental Set. The program’s default settings were used for XPac, and a 20-molecule coordination sphere, 20% distance tolerance, and 20° angle tolerance was used for Mercury. The XPac matrix of pairwise similarities had the structures reordered to group together clusters of higher similarity and the same ordering used for the matrix of *n* values from the calculation of RMSDn in Mercury (Figure S22 and Excel spreadsheet in Supporting Information).

## 3 Results

### 3.1 Data Set of Chalcones Being Analyzed

The list of 232 experimental crystal structures analyzed in this work is given in Table S2 of the Supporting Information. This Experimental Set constitutes 216 different compounds, of which 12 compounds are dimorphic and two are trimorphic. In addition, 16% of the experimental structures have *Z*′ > 1 (33 structures are *Z*′ = 2, two are Z′ = 3, one structure is *Z*′ = 4 and another is *Z*′ = 5; see Table S3 in Supporting Information), and so some crystal structures contribute multiple conformations to the set of 277 molecular conformations. The most common substituents of the molecules in the Experimental Set are Br (67), Cl (61), NO_2_ (45), OH (41), OMe (39), and F (36). The full set of substituents is given in the Supporting Information, both within Table S2 (full list of structures) and in Figure S2 (breakdown of numbers of each substituent).

**Figure 3:**
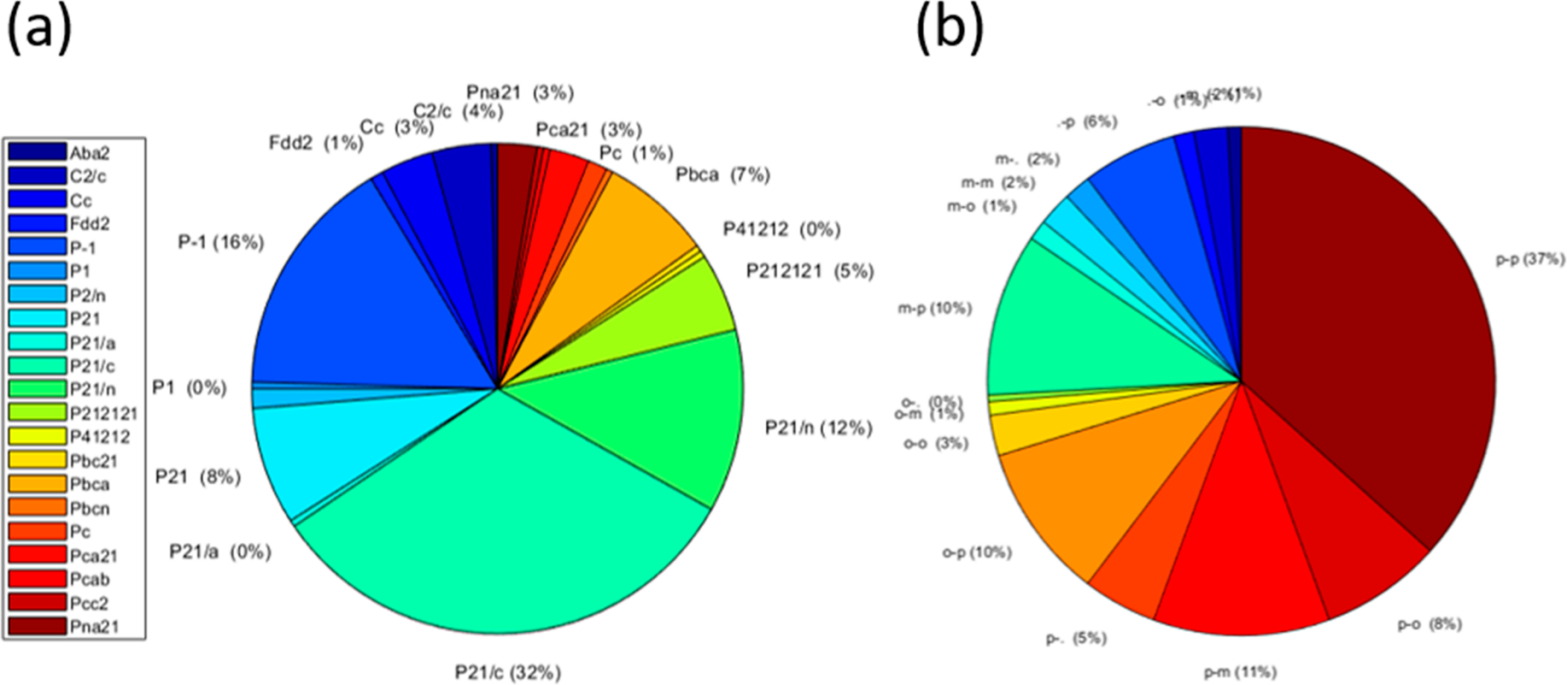
(a) Pie chart showing the space groups (as given in the CSD) of the Experimental Set. For clarity, not all space groups are labeled on the pie chart. (b) Frequency of different combinations of substituent positions within the Experimental Set with o for ortho, m for meta, p for para, and (.) for no substituent. The first letter is the position of the substituent on the 1-ring, and the second letter is the position of the substituent on the 3-ring.

Figure 3a shows that the distribution of space groups in chalcone crystal structures is very similar to that observed for small organic molecules overall,^70^ with only 20% of the crystal structures having a unit cell containing more than four asymmetric units (equivalent to molecules for *Z*′ = 1). Figure 3b shows that para substituents dominate. This may reflect less interest in synthesizing molecules with ortho and meta substituents or greater difficulties in forming crystals suitable for structure determination.

### 3.2 Crystal Structure Prediction

**Figure 4:**
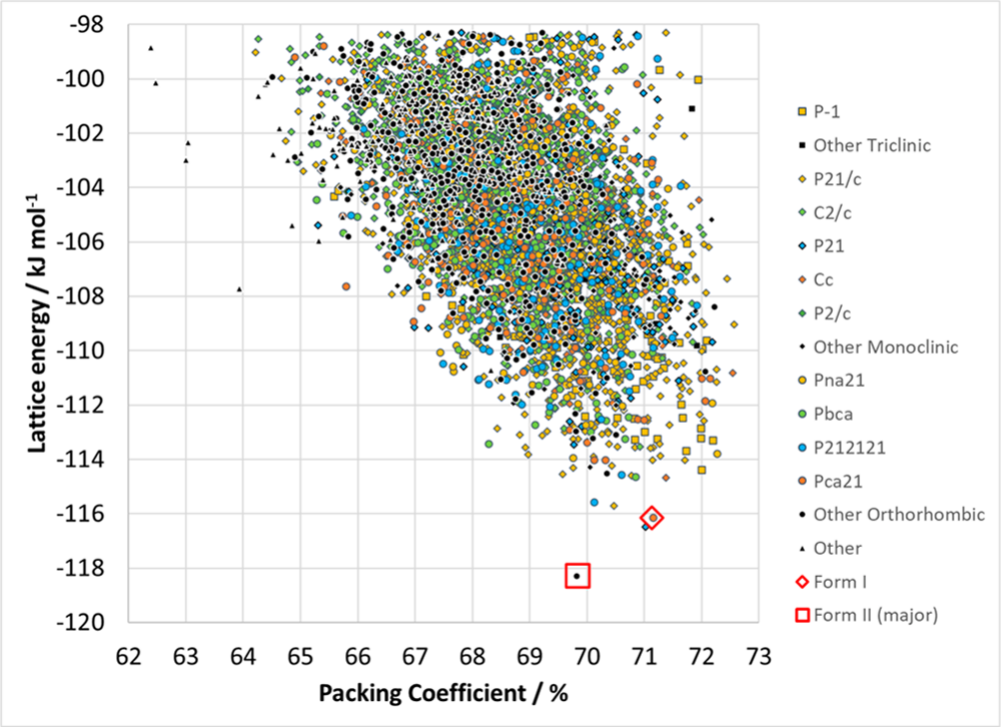
A summary of the CSP lattice energy landscape of the unsubstituted chalcone. Each point represents the lattice energy and packing coefficient of a crystal structure that is a minimum in the lattice energy, denoted by the space group. The lattice energies of the dominant component of form II and that of form I are also shown as open symbols. The minor (12%) component of the disordered form II had an energy of −91.7 kJ mol^–1^ and so is outside the energy range shown.

The lattice energy landscape of the CSP search of the unsubstituted chalcone (Figure 4) is a successful prediction of the crystal structures of the unsubstituted chalcone. Form II has a 88:12 disorder in the enone C=O and C–H bonds in the determination with the best *R*-factor (A-1–*Pbcn*^71^) and contains some contacts where aromatic rings have some π-overlap with the enone part of an adjacent molecule. The major component of form II is found as the global minimum in the CSP. Form I (A-1–*Pbc*21^72^), a structure without any face-to-face aromatic or T-shaped aromatic contacts, is the third lowest energy structure, only 2.2 kJ mol^–1^ higher in energy than form II. This positioning of the known structures of chalcone in the CSP (Figure 4) shows that the model for the lattice energy is realistic. While there are only a few structures that are sufficiently competitive in energy to be possible polymorphs for the specific molecule, there are clearly a vast number of ways of packing the chalcone core that are quite favorable.

### 3.3 Conformation

**Figure 5:**
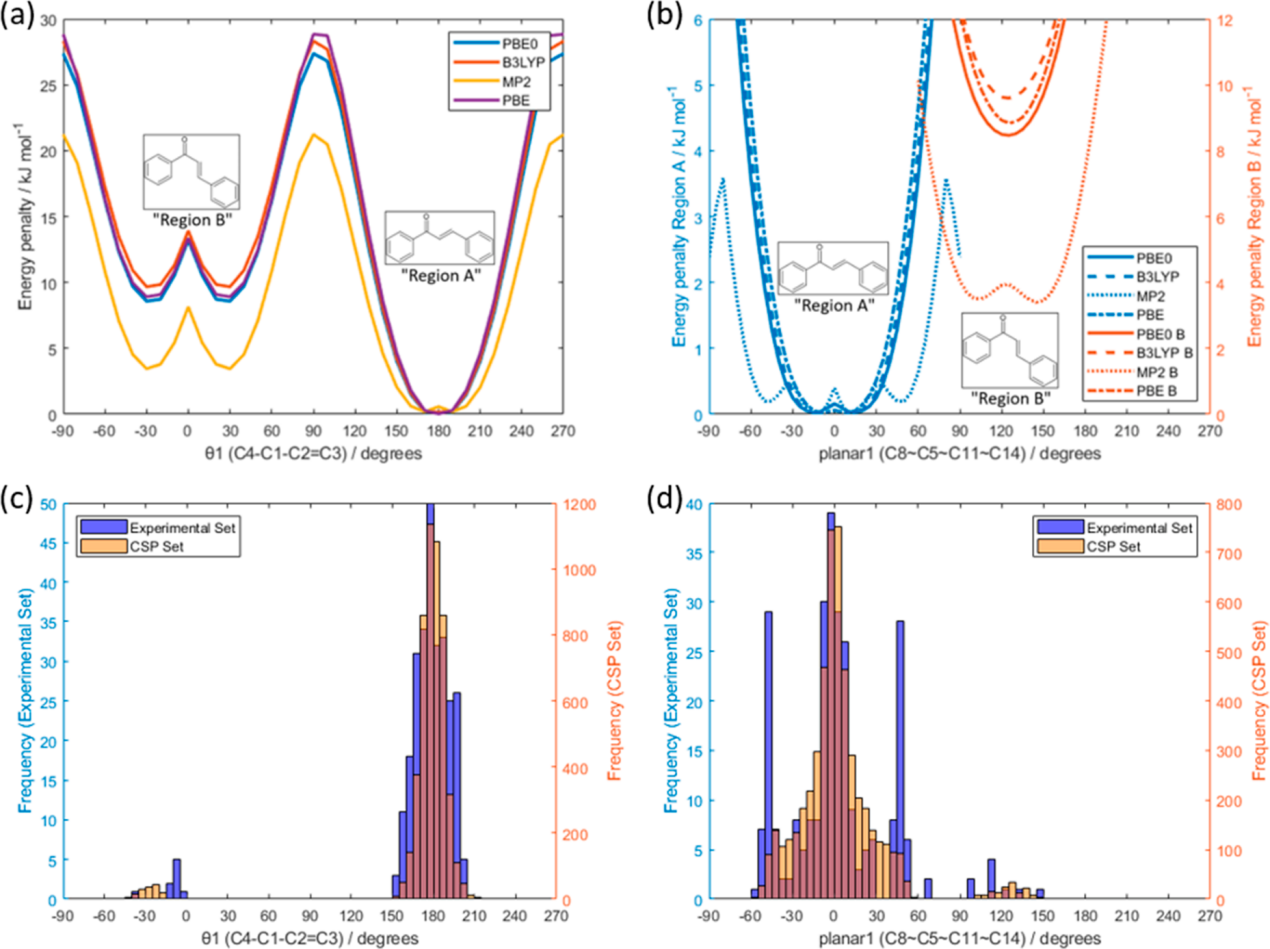
Relaxed torsion angle scans about (a) θ_1_ (defining Region A and B) and (b) planar1 (starting from conformational minima in Region A and Region B) at the PBE0, B3LYP, MP2, and PBE levels of theory with the 6-31G(d,p) basis set. The conformational energy penalty is calculated relative to the global minima in the gas phase optimized geometry. Distributions of (c) the C4–C1–C2=C3 angle (θ_1_) in the Experimental Set (blue) and CSP set (orange) and (d) the C8–C5–C11–C14 angle (planar1) in the Experimental Set (blue) and CSP set (orange). 360° has been added to all angles below −90°, so all angles have a range of −90° to 270°. For the planar1 angle in parts (b) and (d), only one of the two symmetry-equivalent ranges of values was used, so the conformational regions could be visually separated.

The central angle of the molecule, θ_1_ (C4–C1–C2=C3), shows two minima in the torsion angle scan, at 180° and ±30° (Figure 5a), dividing the conformational space into Regions A (s-cis) and B (s-trans). The vast majority of conformations in the experimental crystal structures have the molecule in the Region A conformation. This would be expected as Region B is higher in energy, but the complexity of the balance of the conformation and packing is exemplified by the *Z*′ = 3 structure of Jm8p (1-(3-hydroxyphenyl)-3-(4-nitrophenyl)prop-2-en-1-one), which has two molecules in Region A and one in Region B.

Both phenyl groups have a tendency to be coplanar with the central atoms of the molecule but can vary by ±30° with only a small energy penalty (see Figure S3 in the Supporting Information). Hence, the overall shape of the molecule, as measured by the torsion angle between atoms in the two phenyl rings, planar1, can vary significantly with only a small conformational energy penalty (Figure 5b), such that a wide range of conformations could plausibly be found in crystal structures. This is shown to be the case, as the distribution of the crystalline conformers (Figure 5c) is in good agreement, with the conformations within Region A showing a broad distribution of planar1, centered on a planar molecule. The Region B conformers cannot be planar as this produces an intramolecular steric clash between hydrogen atoms. It is notable that there is a significant proportion (24%) of the molecules in the Experimental Set with planar1 around ±50°, a significantly higher proportion than in the CSP set. This feature appears to reduce the qualitative agreement between the trends in the isolated molecule energies and the distributions seen in crystal structures, as is often expected,^73,74^ but rarely demonstrated for such a large number of closely related molecules. There are an increasing number of examples^75−78^ of a lack of agreement between conformational profiles and observed crystal structures, and the likely causes can be molecule-dependent.^74,79^ In this case, we note that the variation in the calculated molecular conformational surfaces with method is significant, though not unprecedented.^80^ The MP2 conformation scan has local minima such that molecules with a planar1 angle of ±50° are not only low in energy but also two crystals, one with planar1 around 0° and the other with planar1 around 50°, would be considered conformational polymorphs. The high frequency of experimental crystal structures with planar1 close to ±50° thus could reflect the low energy of that conformation or a packing effect.

**Figure 6:**
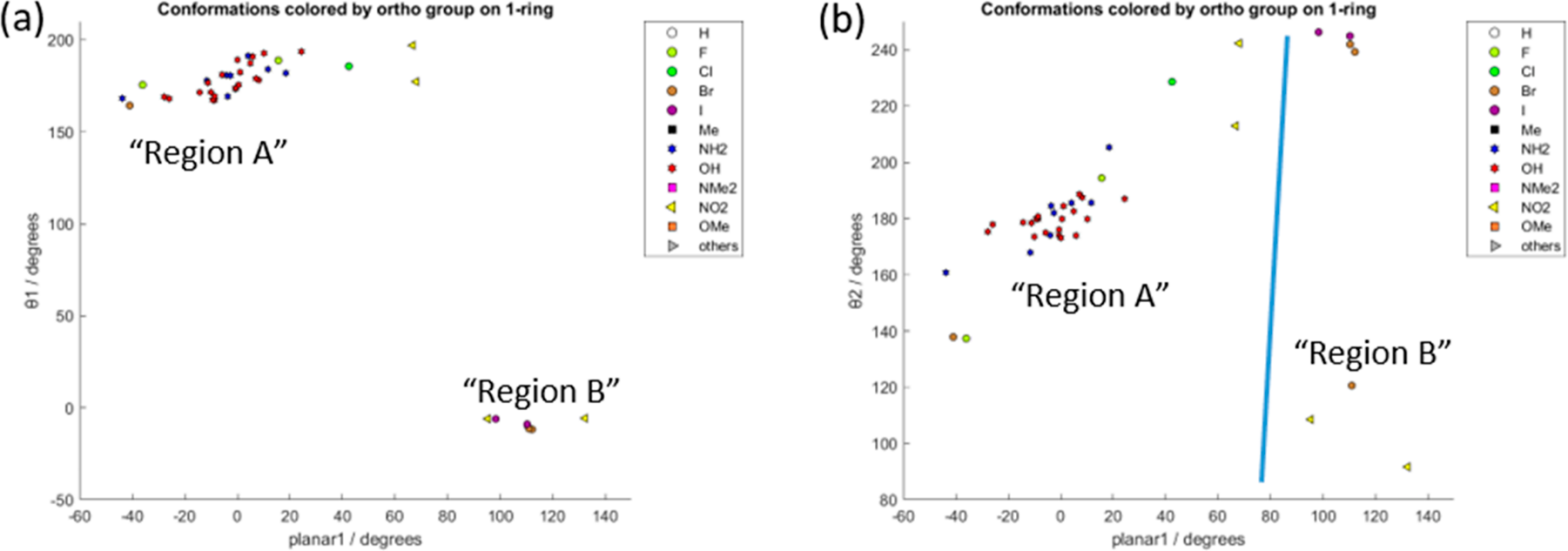
Conformations of the ortho-substituted molecules in the Experimental Set, plotted as the overall planarity of the molecule (planar1, C8–C5–C11–C14) versus (a) the main torsion angle in the molecule (θ_1_, C4–C1–C2=C3) and (b) the angle between the 1-ring and the backbone (θ_2_, C5–C4–C1–C2). Data points are marked by the substituent in the ortho position on the 1-ring. Comparable plots for substituents in other positions of the 1-ring and for substituents on the 3-ring are given in the Supporting Information, Section S7.3.2. 360° has been added to all angles below −90°, so all angles have a range of −90° to 270°.

Figure 6 shows the only significant correlation between the crystalline molecular conformation and the type and position of substituents. (Other plots are given in the Supporting Information, Figure S7.) If the ortho substituent on the 1-ring is amino or hydroxy, it can form an internal hydrogen bond to the carbonyl oxygen, resulting in an unusually narrow spread of θ_2_ values. Larger ortho substituents on the 1-ring, such as chloro or nitro, cause θ_2_ to deviate more, and nitro, bromo, or iodo substituents can cause the entire molecule to adopt the Region B conformation.

### 3.4 Dimer Motifs

**Figure 7:**
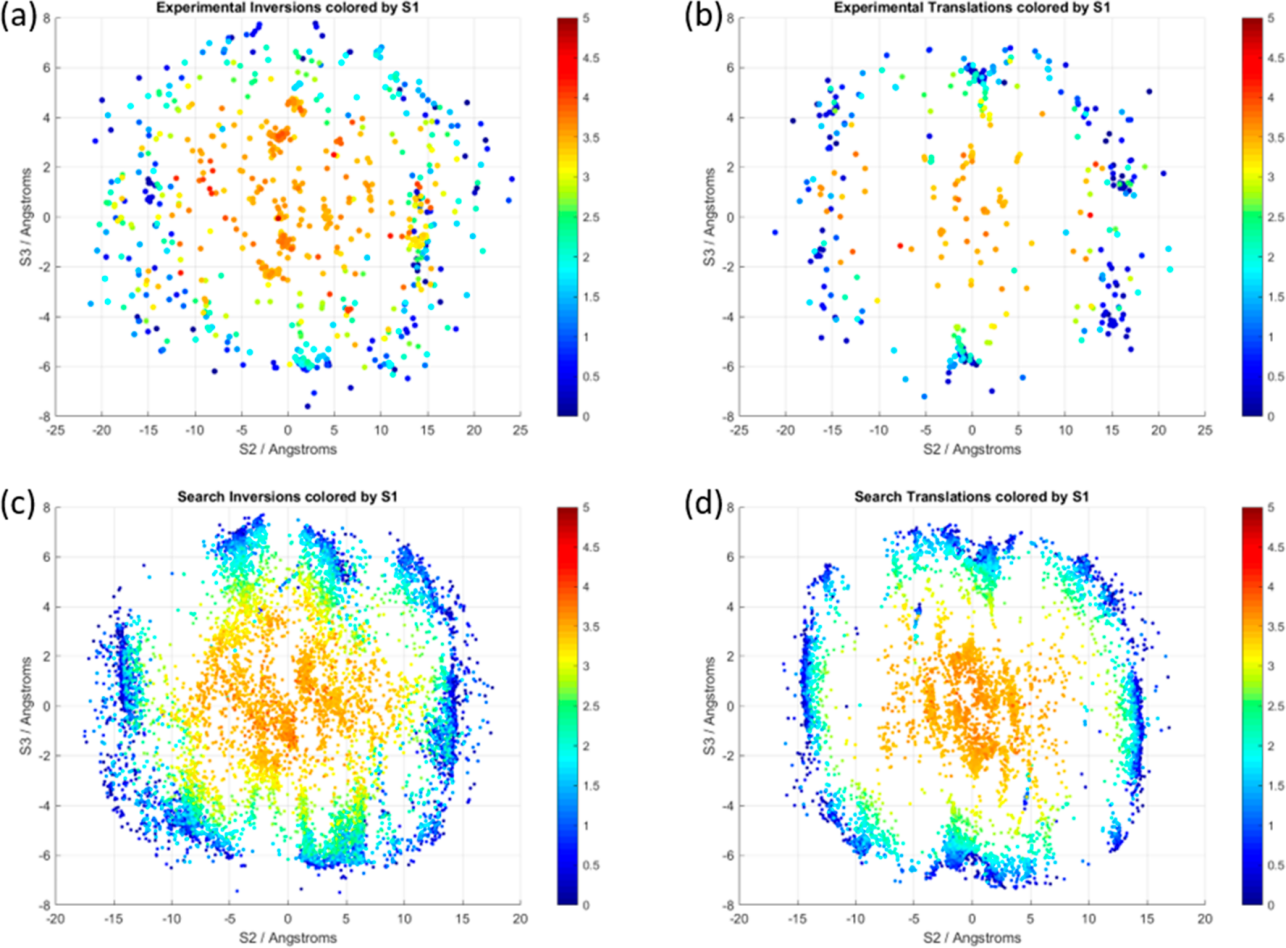
Inversion ((a) and (c)) and translation ((b) and (d)) contacts in the Experimental Set ((a) and (b)) and CSP Set ((c) and (d)) crystal structures, plotted as S2 vs S3, and colored by S1. All distances are in Å. The scale of color used for S1 is constant, to allow comparison of the plots, although no points with S1 > 4 are in the CSP Set. For the plots of translation, the plots are approximately rotationally symmetrical due to a translation relationship between molecules occurring on both sides at equal distances. Since only one of these dimer relationships is included (arbitrarily chosen by the CCDC Python API), the plots are not completely symmetrical, but any point at S2, S3 is equivalent to a point at -S2, -S3.

All pairs of molecules where the two molecules were related by inversion (C12–C2···C2′–C12′ = 180°) or translation (C12–C2···C2′–C12′ = 0°) symmetry that lay within the first coordination ellipsoid (see section 2.4.2) were plotted in Figure 7. Overall, there are 747 inversion and 408 translation contacts in the 232 structures of the Experimental Set and 8540 inversion and 6526 translation contacts in the 4985 structures of the CSP Set (further details of the statistics are in the Supporting Information, Section S7.3.6). The inversion motifs are more numerous than translation motifs because there is the possibility of different inversions on either side of the molecule, and only one of the two symmetry related translations on either side is counted. Structures in chiral space groups will not have any inversion dimers.

There are far more CSP-generated structures than experimental structures, and the substituents of the molecules in the structures of the Experimental Set, being larger than hydrogen atoms to different degrees, increase the end-to-end S2 values by varying amounts. (The larger substituents are predominantly in the para position.) Nonetheless, it is apparent that the clustering of points observed for the CSP structures is reflected in that observed for the experimental structures, albeit with fewer points in the experimental clusters and a more diffuse shape because of the effect of the substituents on the molecular separations. For the face-to-face contacts, where S2 and S3 are approximately 0 Å, the S1 distances would be affected by substituents that are bigger than the aromatic carbons. The variation in S1 in the face-to-face translation contacts is not as great as in the inversion contacts, showing that for the chalcones, translation packing is more space-efficient than inversion, as it is for spoons in a dishwasher.

**Figure 8:**
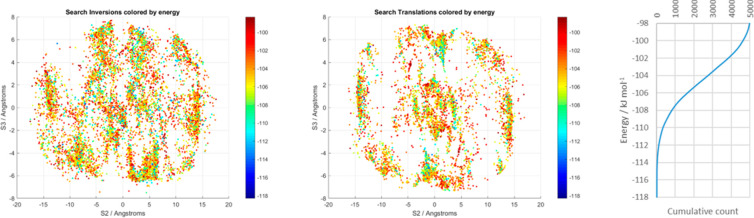
Scatterplots for S1, S2, and S3 dimer contacts in the CSP Set (cf., Figure 7c,d) colored by lattice energy.

Figure 8 shows the same plots for the CSP Set as Figure 7c,d, with the points colored by the lattice energy of the crystal structure. There is very little correlation between the dimer contacts in the structures of the CSP set and the overall lattice energy, showing that the energy is not dominated by one dimer contact but has significant contributions from many molecule–molecule contacts within the crystal structures.

#### 3.4.1 Common Dimer Contacts

The 3-D space defining the close contacts within the Experimental Set and CSP Set (Figure 7) was divided into boxes, spaced at 0.25 Å intervals in S1 and 1 Å intervals in S2 and S3. All the close contacts within each volume were counted, and boxes containing 5+ interactions for the Experimental Set or 30+ for the CSP Set are defined as highly populated. Further details are given in Section S7.3.7 of the Supporting Information. The dimers that fall in the most populated areas of the plots in Figure 7 are given in Table 1. The points nearer the centers of the plots in Figure 7 (small S2, S3, large S1, named I1, I2, I3, and T3 in Table 1 correspond to the motifs that will have a larger degree of π-electron overlap and are analyzed as π···π stacking motifs in section 3.4.1.1. The other common dimer geometries, seen at the top and bottom of Figure 7b and Figure 7d (small S2, large S3, variable S1) correspond to motifs named T1 and T2 in Table 1 and are analyzed as side-to-side contacts in section 3.4.1.2.

**Table 1:**
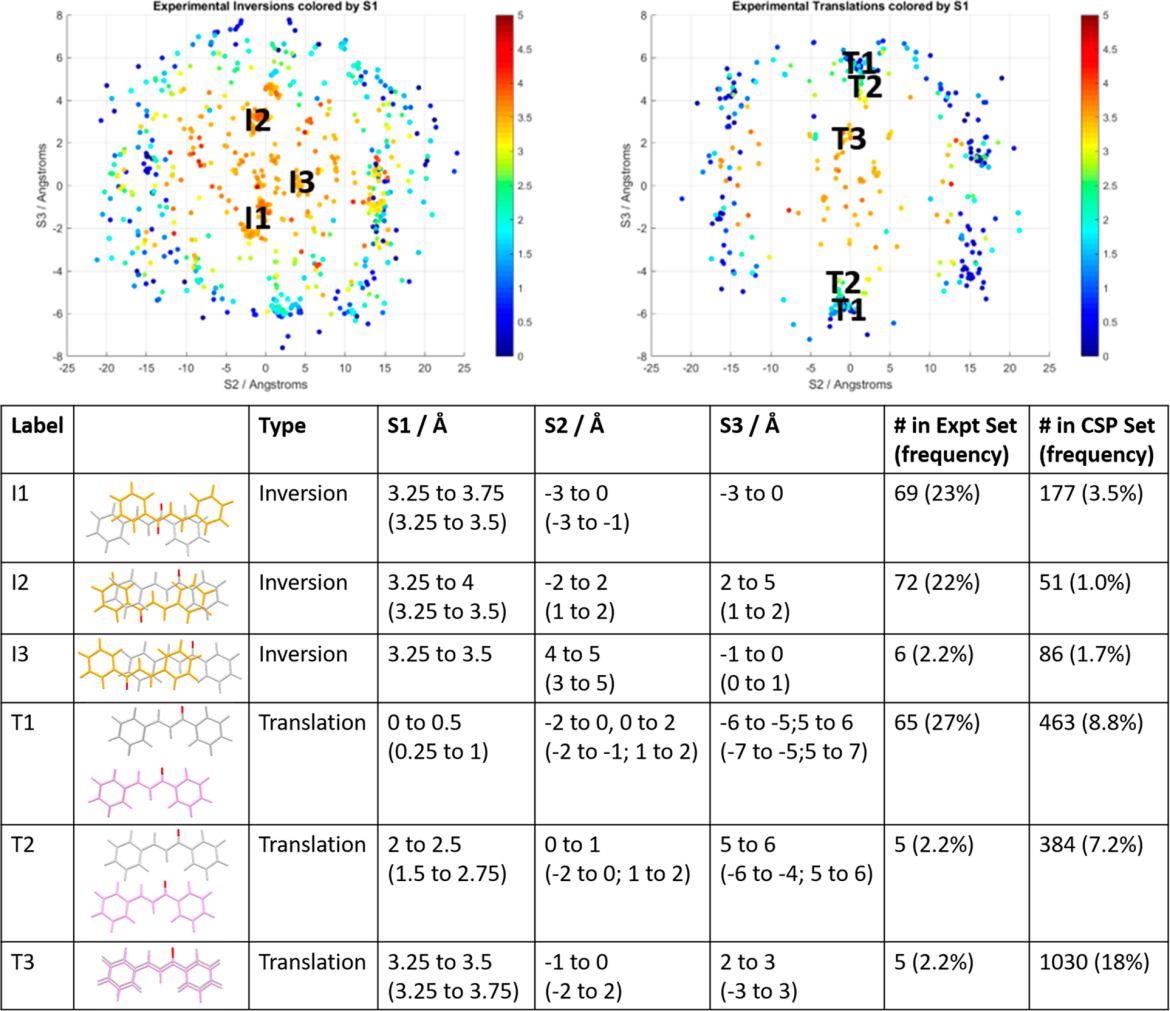
Definitions and Frequencies (in Experimental and CSP Sets) of the Most Populated Areas of the Plots in Figure 7^a^

a Definitions in terms of S1, S2, and S3 are given for the Experimental Set, with the definitions in parentheses for the CSP Set where they differ. Diagrams showing these definitions on the 3-D plots of contacts are included at the top. The number of contacts of this type is given, with the percentage of structures containing that type of contact. Structures may contain the same contact type more than once.

##### 3.4.1.1 π···π Stacking (Face-to-Face Contacts)

As seen in Table 1 (based on Figures S15–S18 and Table S11 of the Supporting Information), there are three distinct inversion relationships that have extensive overlap of the conjugated systems. These do not form a continuum, and there are regions between the populated areas of the coordination ellipsoids that do not have many contacts on Figure 7. There are also translation contacts, named as T3 in Table 1, which cover a broader range of S2 and S3 values in the CSP structures. It is possible for a crystal structure to have more than one of the inversion contacts listed in Table 1, although having translation contact T3 precludes having any of the inversion contacts I1, I2, or I3.

Within the Experimental Set, 68 structures contain at least one face-to-face inversion and 5 structures contain the T3 face-to-face translation. This totals 31% of all structures in the Experimental Set containing face-to-face contacts. Within the CSP Set, 297 structures contain at least one face-to-face inversion, and 935 structures contain the T3 face-to-face translation, totaling 25% of structures. Thus, the vast majority of structures do not contain substantial amounts of π overlap in the inversion or translation contacts.

##### 3.4.1.2 Side-to-Side Contacts

Two common van der Waals contact dimer geometries are T1 and T2 (Table 1). These are translation contacts and differ in the separation in S1. T1 contacts have the molecular planes almost coinciding (<1 Å separation), and almost all have planar1 ∼ ±50°, whereas T2 has the molecular planes separated by between 1.5 and 2.75 Å and planar1 ∼ 0°. In Figure 7b, this is seen at the bottom middle and top middle, where S3 distances are able to get smaller as S1 distances increase. These two types of contacts look like a continuum in the 2-D plot (Figure 7), but Table S11 and the 3-D plots (Figure S16, Figure S18, Figure S19) show that the regions with S1 between 0.5 and 2.0 Å in the Experimental Set (and between 1.0 and 1.5 Å in the CSP Set) do not fulfill the criteria for being highly populated. Hence, there is a subdivision into T1 and T2 dimers, but this depends on the quantitative definition of “highly populated”. In the case of the translation dimers, equivalent contact on both sides of the molecule form 1-D motifs. Hence, we can define a 1-D translational motif with molecules side-to-side, TrSS, which covers both T1 and T2, above, and can be subdivided by molecular conformation. The TrSS_twist_ subset of structures almost all contain the T1 motif defined in Table 1.

As only translational dimers will necessarily form a 1-D motif, it is necessary to use XPac analysis to find other common extended motifs, such as chains, ribbons, sheets, or layers (section 3.5).

#### 3.4.2 Analysis of CSP Structures in Terms of Common Translational 1-D Motifs Seen in the Experimental Set

**Figure 9:**
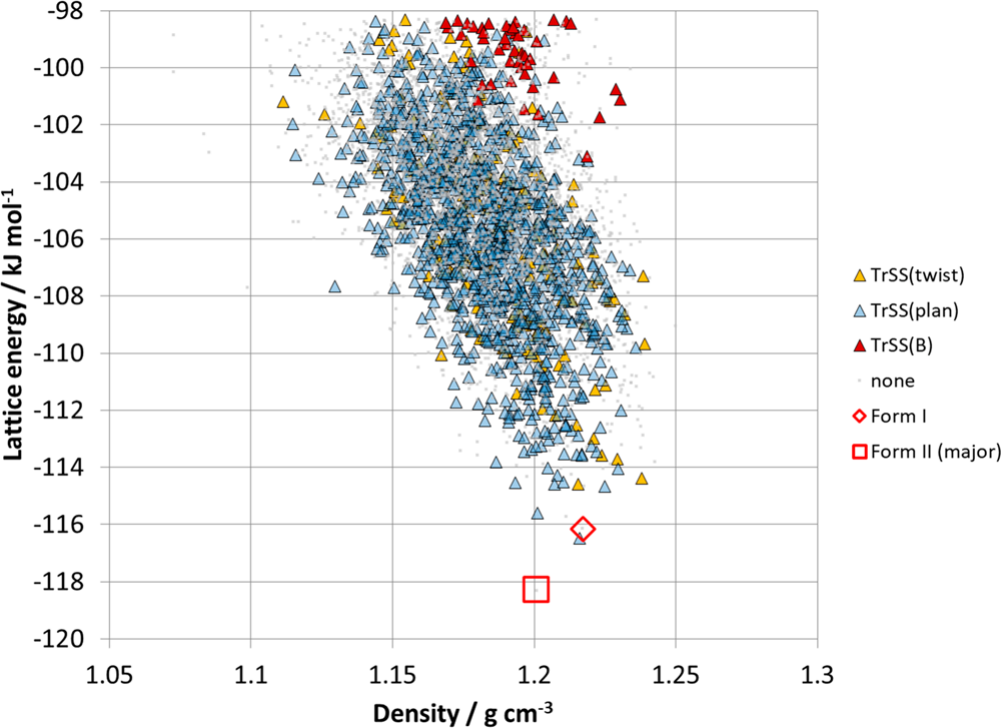
CSP landscape with points classified by whether the crystal structure includes the TrSS motif or not and the conformation within the motif.

The distribution and nature of common inversion and translation dimer motifs in the CSP Set are the same as that seen in the Experimental Set (section 3.4), indicating that the main driving force for the packing is the chalcone backbone. Any link between the dimer motifs and the energies of the crystals can only be assessed for the CSP structures. Figure 9 shows how the TrSS motifs with the planar molecule (TrSS_plan_), twisted molecule (TrSS_twist_), and related motif with the Region B conformations (TrSS_B_) are distributed among the computational structures. There is a significantly higher proportion of structures with the TrSS_plan_ motif than in the Experimental Set, which may be an artifact of the CSP method (see section 4.1). The TrSS_B_ motif is seen in a small number of structures, and these are high in energy. However, the lowest energy structure with the Region B conformational type has an energy of −105.7 kJ mol^–1^, and the lowest energy with the TrSS_B_ motif has an energy of −103.1 kJ mol^–1^, so this motif is not particularly unfavorable for this conformation.

### 3.5 Pairwise Comparisons to Identify Extended Motifs, up to Isostructural Crystals

**Figure 10:**
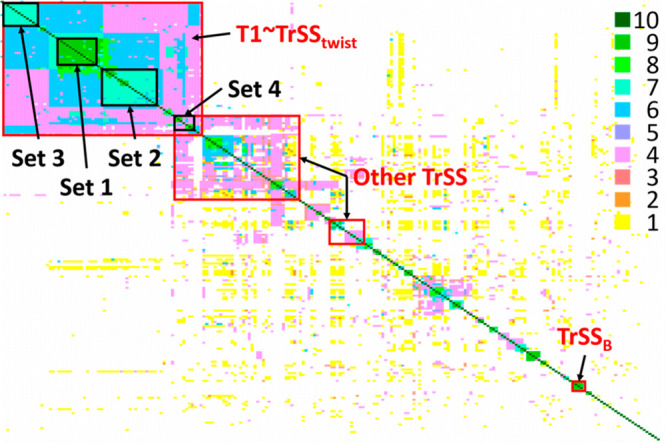
Heatmap of pairwise XPac analysis. The key is degree of similarity, from 1 = common dimer to 9 = 3-D similarity, with 10 for identical structures (i.e., the diagonal). Regions of high similarity where most of the structures contain a variant of the TrSS motif are labeled. The Set labels refer to the groups defined in the isostructurality analysis in Figure 12. (As the Set 4 structures are contained within both the T1 and the Other TrSS sets, they are also included in the analysis in Figure 13.) A large version of this figure with full definitions is in Section S9 of the Supporting Information, and an expandable version is provided in the Excel spreadsheet Supporting Information file.

The analysis for the common one and higher dimensional similarities between pairs of crystal structures, carried out using XPac, is summarized by the heatmap in Figure 10. This is compared with the corresponding diagram for the number (*n* ≤ 20) of molecules which are overlaid in Mercury’s Crystal Packing Similarity RMSDn analysis in Section S9 of the Supporting Information. The key finding is that the large groups of structures with significant structural similarity on Figure 10 contain the translation motif, TrSS. It is also notable that the XPac analysis of periodically repeating motifs gives a similar distribution of similar structures to the Mercury analysis in terms of the coordination sphere (Figure S22). Hence, this TrSS motif is the most common motif found in both the experimental and CSP crystal structures, seen in approximately 50% of crystal structures in the Experimental Set.

**Figure 11:**
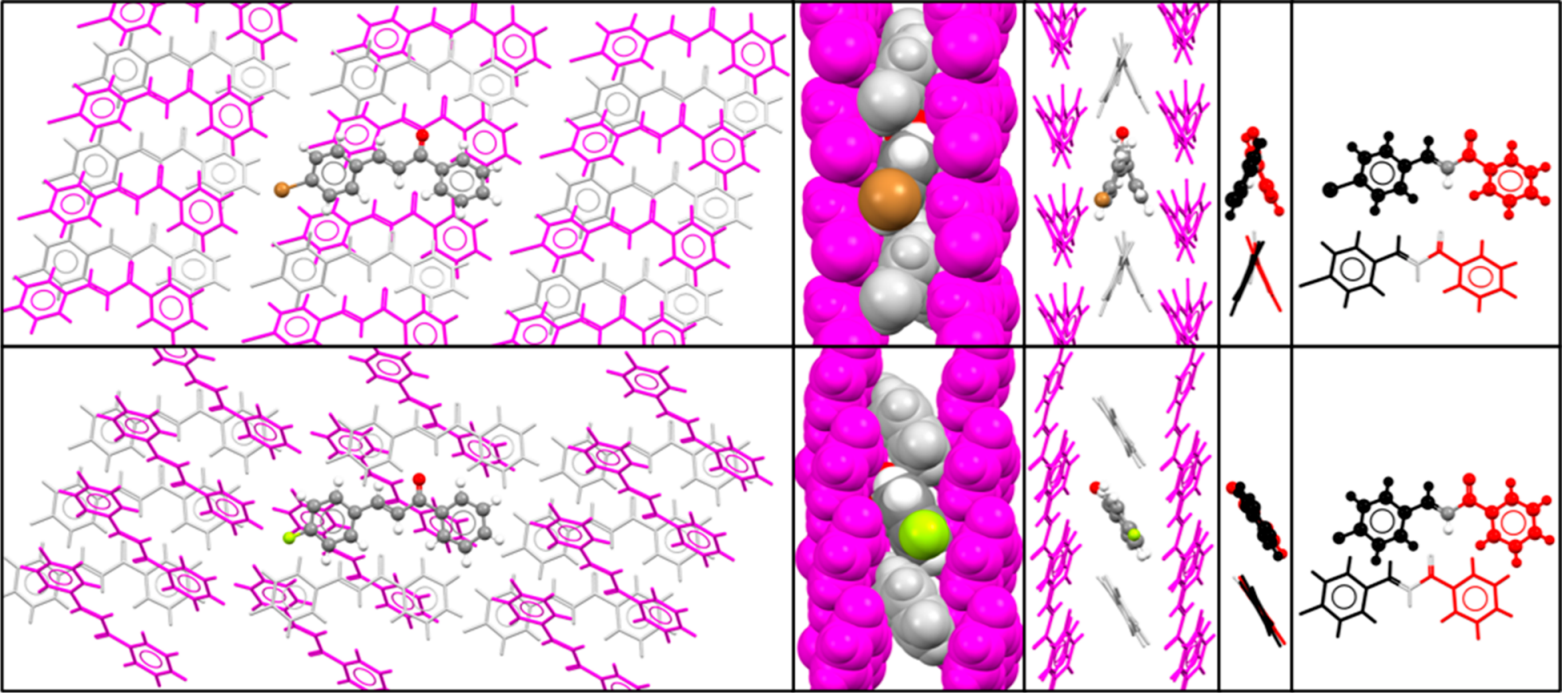
Various views of (top) A-4p(3-(4-bromophenyl)-1-phenyl-2-propen-1-one), which has the TrSS_twist_ motif and (bottom) A-2p-I (3-(4-fluorophenyl)-1-phenyl-2-propen-1-one), which has the TrSS_plan_ motif. Molecules are shown in gray where they are related by a translation to the molecule in atomic colors and in magenta for the glide related molecules. The final views on each line are a pair of molecules extracted from the third and first views respectively, with the 1-ring colored red and the 3-ring colored black to show that the corresponding rings in adjacent molecules of the 1-D packing motif have virtually equivalent relative orientations, regardless of the molecular conformation.

Figure 11 shows comparable views of crystal structures containing the TrSS_twist_ and TrSS_plan_ motifs. The spacefilling diagram shows that the molecules pack efficiently in this motif. The end-on capped-sticks view of each structure shows how the tilt of the planar molecules with respect to one another is similar to the angle of twist in the twisted molecules so that the relationship between one aromatic ring and the corresponding ring in the next molecule of the 1-D motif is similar, despite the different conformations. The balance of conformation and packing in these two structures gives virtually identical intermolecular interactions. These intermolecular interactions between the chalcone scaffold will not be disrupted by p-substituents, as shown by the bromine and fluorine atoms in Figure 11. Indeed, the molecules that have the TrSS motif contain a wide range of substituents, namely, F, Cl, Br, I, CH_3_, N(CH_3_)_2_, NO_2_, OCH_3_, OH, NH_2_, SMe, Ph, OEt, iPr, CF_3_, OCF_3_ CN, C≡CH, piperidine, OC(=O)C(=CH_2_)CH_3_, O(CH_2_)_3_CH_3_, and O(CH_2_)_4_Br. Almost all are seen in the para position, with a few in the meta position and a very few (namely, hydroxyl and fluorine) in the ortho position for the TrSS_plan_ motif.

**Figure 12:**
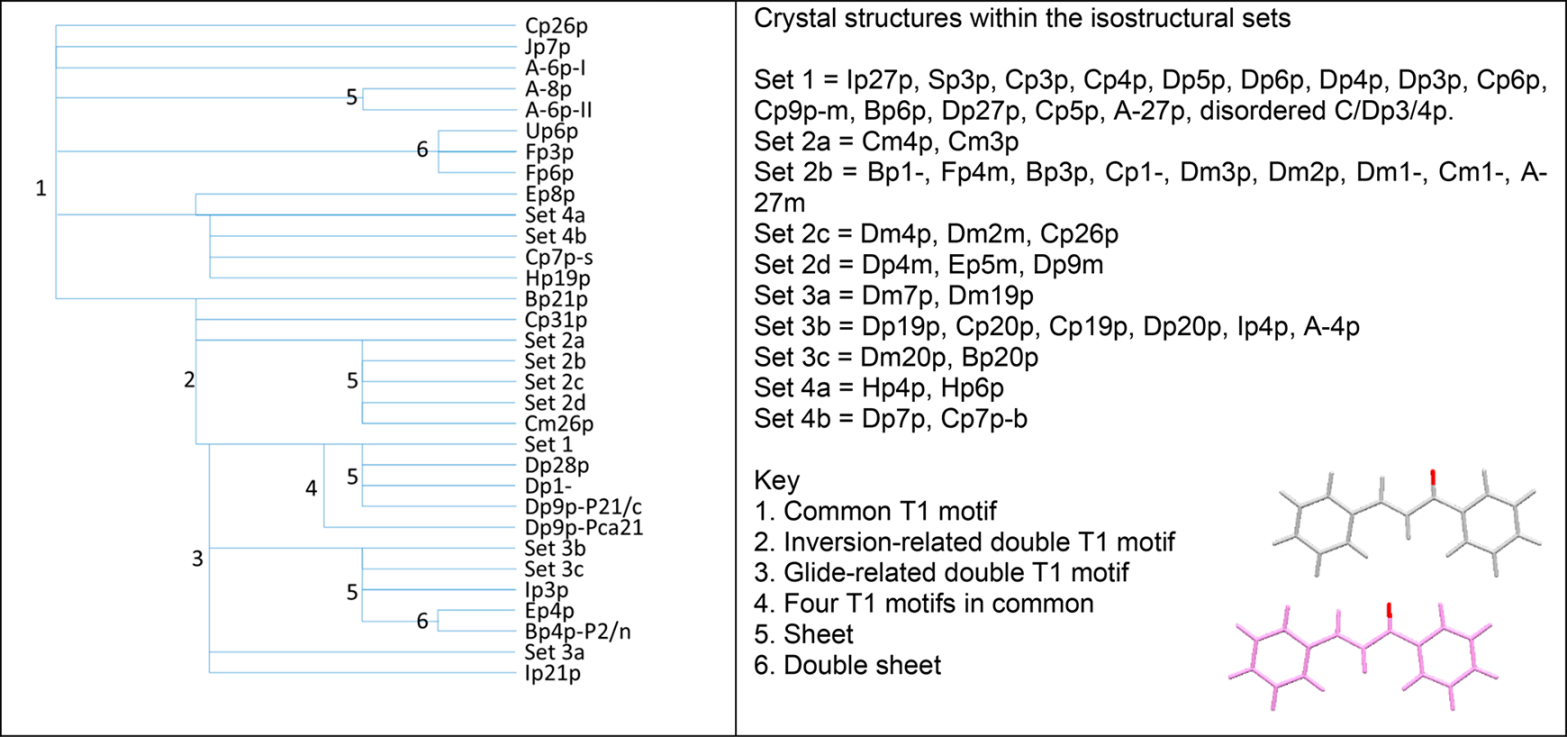
Structural similarities of the 68 crystal structures of the Experimental Set, which contain the T1 contact (and usually the twisted molecule with planar1 ∼ ±50°). Some of the common features are labeled as defined by the key.

**Figure 13:**
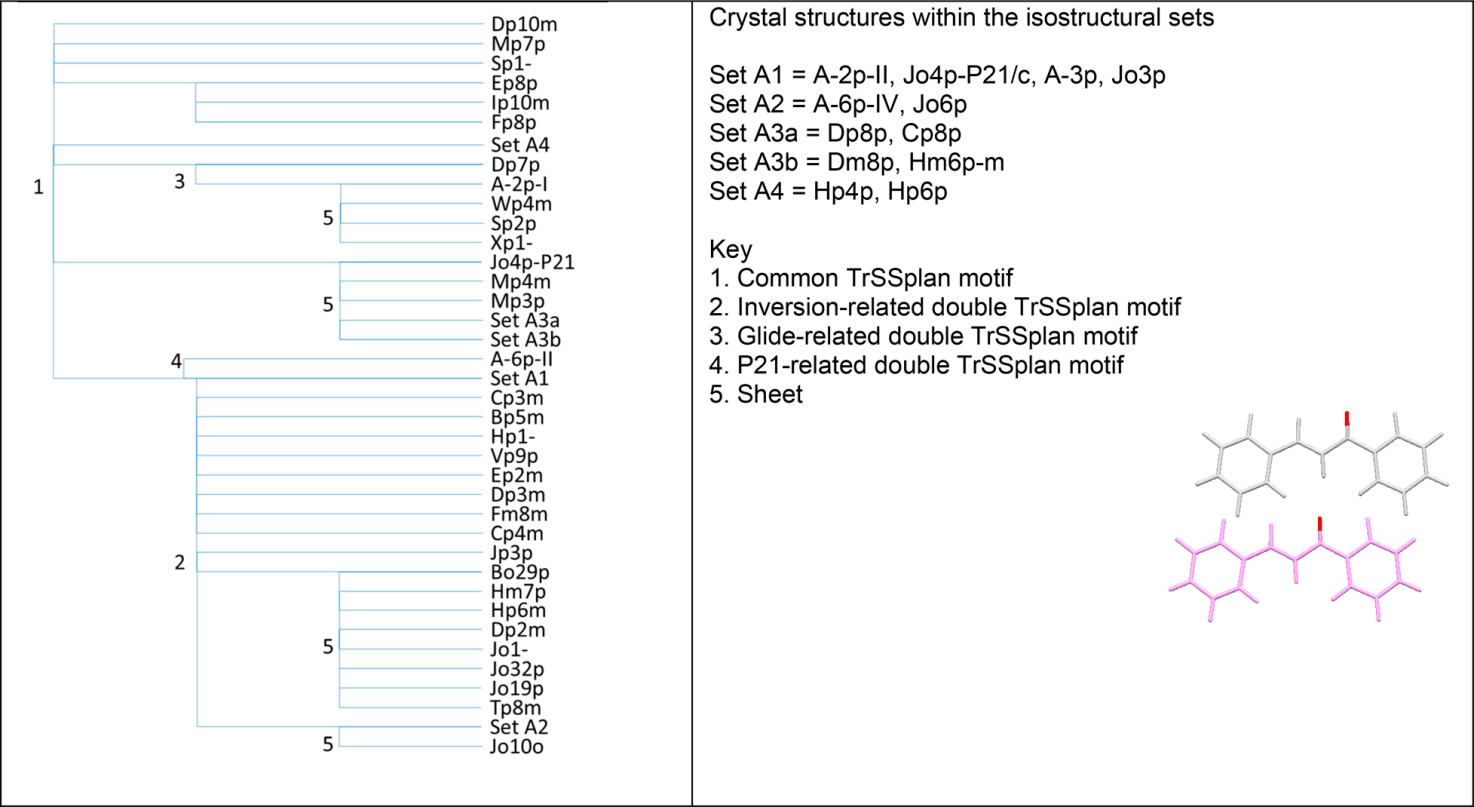
Structural similarities of the 45 crystal structures of the Experimental Set, which contain the Other TrSS contact shown on Figure 10. Some of the common features are labeled as defined by the key.

In the dimer analysis, there is a particularly concentrated area (T1) corresponding to mainly twisted molecules, and a more diffuse set of translation dimers extending beyond the five structures of the T2 dimer motif that completes the TrSS set, the remainder of which mostly contain the planar molecule (see Supporting Information, Section S8). Hence, the crystal structures containing the T1 dimer were analyzed separately (Figure 12) from the rest of the TrSS structures (Figure 13) to see how these translational 1-D motifs build up to form more complex packings and detect isostructurality.

Eight structures contain the Region B conformation (a further structure contains both Region A and Region B conformations), and six of these contain the side-to-side translation interaction that is so similar to the TrSS_twist_ and TrSS_plan_ motifs that it is denoted TrSS_B_. (The different shape of the molecule means that this is not included within the TrSS definition in terms of S1, S2, and S3, Section S8 of the Supporting Information). Of these, four are isostructural, and a fifth contains a sheet in common with them.

The other isostructural groups which do not contain the TrSS or related TrSS_B_ motifs are (Cp3o, Ip1-, Ip2o, Ip2p, Ip3o), (Cp21p, Dm21p, Dp21p), (Jm2p, Jm3p, Jm6p), and (Fp2p, Fp9p, Up9p) and a few isostructural pairs that can be read from the spreadsheets in the Excel spreadsheet Supporting Information file.

## 4 Discussion

It is obvious from looking at only a small sample of chalcone structures that there is no simple strong packing preference, such as a favored π···π stacking of the chalcone core. This study has analyzed a large number of experimental crystal structures, most determined because of the pharmacological and optical applications of chalcones but supplemented with a significant number of new structures from our collaborators. The sample is limited to only one substituent per ring, with the substituents limited to being a phenyl ring or smaller, i.e., less than half the size of the core, so that we could study the preferences of the core in determining the packing. The sample was dominated by para substitution and halide substituents (Figure 3b, Figure S2); i.e., the position and nature of the majority of substituents would be expected to have a relatively minor effect on the conformation and relatively weak substituent-specific intermolecular interactions. However, this study faced the challenge of identifying common motifs in a large set of crystal structures, which are not formed from specific intermolecular synthons, such as hydrogen bonding, but are held together by more diffuse, dispersion-dominated interactions. The large number of chalcone crystal structures reveals that there is a wide variety in the packings. However, this variety is closely mirrored by the structures generated by the CSP study of the unsubstituted chalcone. This shows that the packing is dominated by the core, but since this has a wide variety of packings of similar stability, the packing changes necessitated by the substituents produce a wide range of observed structures. Since there are relatively few extensive sets of crystal structures of similar molecules, we cannot yet say whether this observation is general. However, we do note that the crystal structures of derivative families of compounds often show a surprising overall diversity of crystal structures^75,81,82^ which would warrant comparing with a CSP of the core.

### 4.1 Conformation

The substituents affect the relative energies of the different molecular conformations. An ortho hydroxy or amino group on the 1-ring will form an intramolecular hydrogen bond strongly favoring a planar conformation; yet, a large ortho substituent will clearly lead to a steric clash if the molecule is planar. Larger groups in the ortho position of the 1-ring lead to a complete change in the shape of the molecule to the B conformation as seen for the molecules with iodo or nitro at that position (no groups larger than this were found at the ortho position in the data set examined). Four of the eight crystal structures that contain the Region B conformation were isostructural, with a further two containing a 1-D side-to-side translation contact, similar to TrSS. For cases where the conformation was not dictated by steric clashes or internal hydrogen bonding, there could be an electronic effect. However, there was no correlation of greater planarity with Hammett parameters as might be expected if the deviation from planarity was purely a molecular property (see Supporting Information, Section S7.3.3).

The large number of twisted molecules (planar1 ∼ ±50° in Region A) in the experimental crystal structures is somewhat surprising, given the general tendency of crystal packing to favor extended conformations^79^ as well as the expectation that the chalcone molecule would be planar because of its extended π system. The energy penalty for twisting the molecule is smaller than for adopting the Region B conformation, but both are dependent on the ab initio method (Figure 5), with the MP2 calculation producing a local conformational energy minimum around the twisted conformation. Correctly estimating the intramolecular dispersion, which would favor the twisted conformation, is demanding of the ab initio method. The PBE0 functional may overstabilize the planar conformation through a delocalization error (although this error is usually less for PBE0 than PBE,^83−86^ the difference appears to be small for the isolated chalcone molecule, Figure 5). The MP2 calculations are likely to be affected by an intramolecular basis set superposition error^87^ and are still far from the accuracy that can be needed for accurate CSP molecular calculations.^83^ However, if we assume that the PBE0 functional calculations used in the CSP are overestimating the intramolecular energy penalty for twisting the molecule, this would at least partially account for why there is a smaller proportion of twisted molecules in the CSP than experimental structures (Figure 5). The ability of the molecules that are twisted by 50° to close pack with translational symmetry for a variety of substituents in the TrSS_twist_ motif (Figure 11), may well be a more important factor in explaining the large number of twisted molecules in the Experimental Set.

Whether or not there is another conformational minimum around 50° in the isolated molecule PES surface would affect the definition of conformational polymorphs^88^ as polymorphs where the molecular conformation would optimize to different conformational minima. In this case, whether polymorphic pairs are conformational polymorphs depends on the ab initio method used (Figure 5, Table S4). So the pairs of polymorphs, A-6p-I and A-6p-IV, and Bp4p-*P*21/*c* and Bp4p-*P*2/*n*, are conformational polymorphs on the MP2/6-31G(d,p) but not on the PBE0/6-31G(d,p) conformational energy surface. There is the same difference in the chalcone backbone in the polymorphs of Cp9p-o and Cp9p-m, but since the orientation of the methoxy group also differs, these are unambiguously conformational polymorphs (Supporting Information, Section S3.3). The dependence of intramolecular energies on the computational method exacerbates the problem of accurately calculating polymorphic energy differences and obtaining an accurate ranking of structures in CSP.^83^

### 4.2 Crystal Packing

#### 4.2.1 Discussion of Isostructurality

The unsubstituted molecule contains no hydrogen-bond donor atoms, and only one acceptor, so only the hydroxy and amino substituted molecules are capable of forming strong hydrogen bonds which are expected to determine their crystal packing. Hence, it is noteworthy that A-10m-II, which has the hydroxy group in the meta position of the 3-ring hydrogen bonding to the central carbonyl of a neighboring molecule, is isostructural (RMSD_30_ = 0.34 Å) with one of the polymorphs of the unsubstituted chalcone. Hence, two molecules that would be expected to have very different intermolecular interactions have isostructural polymorphs.

This is just one of the many unusual isostructural pairs of molecules found among the chalcones, in addition to those with the more usual isostructural substitutions of Cl/CH_3_ and F/H. This is illustrated by the largest isostructural group of 15 crystals (Set 1 in Figure 12) given in Table 2. All the substituents are in the para positions on the two rings, and all are halogens or similar-sized groups. There are many structures that might have been expected to be in this isostructural group, but are not, for example A-3p, A-6p (3 polymorphs), A-9p, Bp3p, Bp4p (2 polymorphs) Bp9p, Ip3p, Ip4p, and Ip9p. The conclusions that can be drawn from the isostructurality found in this survey will be limited by the probable lack of polymorph screening for most of the chalcones.^89^ However, the isostructurality found in the detailed study of substituted quinoxalines^40^ involved metastable polymorphs, which emphasizes that any change of substituent does involve a change in the relative thermodynamics and kinetic differences between the observed and any hypothetical crystal structures of similar energy.

**Table 2:** Molecules Which Crystallized in the Largest Isostructural Set and Their Crystal Structures

code	REFCODE	1-ring	3-ring	code	REFCODE	1-ring	3-ring
A-27p	QEDHOY	H	CF_3_	Dp3p	LEPYIP	Br	Cl
Bp6p	MIYCAZ	F	CH_3_	Dp4p	LEHROG	Br	Br
Cp3p	GAVBEL	Cl	Cl	Dp5p	IWALAV	Br	I
Cp4p	GEJJUB	Cl	Br	Dp6p	IZEFOI	Br	CH_3_
Cp5p	TADPEX	Cl	I	Dp27p	PAQJOJ	Br	CF_3_
Cp6p	LOBVEE	Cl	CH_3_	Ip27p	ARUGUR	OCH_3_	CF_3_
Cp9p-m	MEGYON02	Cl	OCH_3_	Sp3p	CIQFEO	SCH_3_	Cl
C/Dp3/4p	a	Cl/Br	Cl/Br				

a A structure that could not be refined to a publishable standard.

#### 4.2.2 Dominant versus Expected Motifs

The dominant packing of the chalcone backbone, the side-to-side translation contacts of the molecules (TrSS, Figure 11), is not seen in the observed crystal structures of the unsubstituted molecule, A-1-, though it is in putative polymorphs, including one that is more stable than form I (Figure 9). This type of 1-D packing is observed with a wide variety of substituents, although not with any ortho substituents when the molecule is in the twisted conformation. Figure 11 shows that these 1-D motifs can accommodate some meta and any size para substituents. The overlap region on Figure 10 and the similarity in the phenyl–phenyl contacts emphasize that TrSS should be considered a single motif, which can accommodate both planar and twisted molecules.

The expected π-overlap of the face-to-face interactions of adjacent molecules is present in many crystal structures and associated with the color,^52^ but there is not a strongly preferred way of overlapping the molecules in this way. The face-to-face inversion-related contacts, I1 and I2 (Table 1), are present in a significant number of structures. However, the maximum π···π overlap (T3 with no offset (S2, S3) displacement) is rarely observed.

### 4.3 Development of Crystal Structure Comparison Methods

This approach of using the CCDC Python API to systematically analyze a subset of the dimer contacts in the entire set of crystal structures, and then manually inspect this to find trends, is an efficient way of analyzing a large data set to find common structural features. This approach is useful for analyzing the large data sets of structures generated in a CSP study but does require some molecule-specific definitions. The chalcone core is very nonspherical, but its approximate planarity and symmetry allow the definition of a coordination ellipsoid with axes S1, S2, and S3 (the interplanar, end-to-end, and side-to-side molecular distances) to determine which molecules are in van der Waals contact. This first application to simultaneously compare a set of crystal structures of different molecules required the limits on S1, S2, and S3 to be adjusted for variations in substituent size. The coordination ellipsoid definition encompassed all inversion and translation pairs in van der Waals contact but did not include all molecule pairs with a different symmetry relationships (Supporting Information, Section S7.3.5).

This analysis of the inversion and translation contacts in the crystal structures showed the structural diversity of the experimental structures and, by comparison with the much larger set of CSP-generated structures of the chalcone core, allowed the identification of some common dimer geometries (Table 1). For many of the crystal structures, the inversion and translation dimers go a long way to defining the overall crystal packing as the other molecules have to complete the overall close packing of the coordination ellipsoid. Nonetheless, the focus on inversion and translation dimers could in principle miss a strongly interacting dimer related by another (or approximate) symmetry element. This was not the case for the chalcones as this would have been detected by the XPac analysis.

The more established methods of comparing crystal structures, Mercury’s Crystal Packing Similarity tool and XPac, have the major disadvantage of being pairwise comparisons. These comparisons then need to be grouped, which is difficult to automate to find common motifs that are not 3-D (isostructurality). For example, where two structures, A and B, have a 2-D similarity, and B also has a 2-D similarity with C, it does not imply that A and C have a 2-D similarity. If A and C contain the same molecule only and are *Z*′ = 1, it can be assumed that they have at least a 1-D similarity (since the two planes in B must intersect at a line). Both XPac and Mercury’s Crystal Packing Similarity tool require parameters values defining cutoffs and hence at least manual inspection that the default values are appropriate for the specific group of molecules. For example, Mercury’s Crystal Packing Similarity tool, using the default 15 molecule coordination sphere, can appear to show isostructurality when it has matched a 15 molecule double layer of very anisotropic molecules, but the double layers can stack differently. This can be avoided by increasing the number of molecules to be matched to 20 or even 30 molecules, but this can exceed the capabilities of desktop PCs for larger molecules. XPac is a valuable complement to coordination environment comparisons as it looks for matching motifs of different dimensions. However, XPac can match alternate molecules and report a 3-D similarity, which is not isostructurality as the unmatched molecules will be different.

### 4.4 Systematics of the Chalcone Packings - Is This Unusual?

The investigation of the packing preference of the chalcone core compared the set of experimental substituted crystal structures with the lower energy structures generated by a CSP study of the unsubstituted chalcone. This showed sufficient similarity in the distribution of geometries of inversion and translation-related dimers to confirm that the substituted chalcones had packing preferences similar to the core. However, there were no dimer geometries associated with particularly favorable lattice energies (Figure 8), implying that there is no strongly preferred dimer interaction.

There is a reassuring consistency that the more common chalone dimer geometries mapped approximately onto the more common motifs identified in the isostructurality analysis using Mercury’s Crystal Packing Similarity tool and XPac. The definitions are different, and since there are some structures that are close to the limits, there can be small differences in the numbers of structures, for example, in T1 and TrSS_twist_. Face-to-face contacts between the chalcone core with some degree of π···π overlap are seen in less than a third of the crystal structures. However, the TrSS translational motif is significantly more common, appearing in approximately half the experimental structures. This TrSS motif is nicely close-packed in a way that is not affected by any para or smaller meta substituents (Figure 11). These substituents will therefore determine how the motif is packed in 3-D. Hence, this translational motif is seen in the crystals of many molecules because it can accommodate a wide range of substituents and can pack in a variety of ways, not because the intermolecular interactions within the TrSS motif are particularly strong.

The observation that the chalcones adopt a wide variety of crystal packings reflects the different spatial requirements of substituents requiring different structures to maintain close packing. Even substituents in the same position and of almost the same size will have different charge distributions affecting the local electrostatic and repulsive forces. When there are many packing possibilities, then very small changes will tip the balance of which structures are the most favorable. What is reassuring is that the packing preferences of the chalcone core are reflected in all the crystal structures (i.e., no structures have been seen with highly unfavorable conformations or poor packing of the core). This is possible because there are a large number of ways of packing the chalcone core, as shown by the CSP (Figure 4, Figure 9). This may be a general finding. If the chalcone core had a very strong energetic preference to be planar and pack in a particular 1-D motif (e.g., π-stack), with a large energy penalty for a sideways shift in the stacking, then would so many substituted chalcones be able to crystallize? There would be relatively few substitution patterns that would enable this motif to crystallize with close packing of the atoms at the edges of the stack and favorable substituent intermolecular interactions. (Solvent molecules might be incorporated into the structure if it would otherwise have large voids around the substituents.) Substituents that were larger than the aromatic carbons would provide a force trying to increase the separation of the π-stacked molecules. Thus, attempts to generate a large library of crystal structures of related molecules may be hampered by many just not crystallizing^38^ if the interactions within the core packing motif were so strong as to make this motif ubiquitous and virtually rigid. The CSD contains hundreds of crystal structures of simple chalcones because the interest in these molecules for their biological and optical properties has coincided with the ability to form at least one crystal suitable for structural determination.

## 5 Conclusion

We have carried out a range of complementary systematic analyses of the crystal structures of 232 chalcone molecules with up to one small substituent on each ring and made comparisons with the low-energy structures generated in a CSP study of the unsubstituted molecule. The similarities between the low-energy crystal structures of the core and the structures of the substituted chalcones confirm that the packing of the core plays a major role in determining the crystal structures. However, there are a wide range of crystal structures in both the Experimental and CSP sets of crystal structures. The molecular conformation can be twisted, with an angle of about 50° between the phenyl rings, as well as the planar conformation expected from the conjugation of the molecule. The diversity of experimental crystal structures is such that no isostructural group contains more than 15 structures, and most contain only a pair of crystal structures. Many of the two dozen crystal structures observed for more than one compound (i.e., isostructural) are unexpected from the similarity in substituent size and position. The most common 1-D motif, observed in half the experimental chalcone structures, is not a typical crystal engineering motif but a translation packing that can be adopted by different conformations of the molecule, with differently sized substituents, particularly in the para position. This study underlines the subtle balance of intermolecular interactions and conformational strain that makes the observed crystal structure quite molecule specific, illustrating why closely related molecules that have similar biological properties will often have very different crystallization behaviors.

## Supporting Information Available

Details of the CSD search. Full details of the crystal structures included in the analysis, with CCDC deposition numbers for as yet unpublished structures. Full details of the CSP. Details of the parameters extracted using the Python code, and the Python codes. Full details and results of analysis of van der Waals contacts and S1, S2, S3 scatterplots, with respect to various parameters including substituent identity and position, and Hammett parameter (PDF)
Common close contacts and higher similarities (XPac and Mercury analyses) as a separate Excel spreadsheet (XLSX)
