# Supplementary material for: Packing Preferences of Chalcones: A Model Conjugated Pharmaceutical Scaffold
Source: Cryst Growth Des. 2022 Feb 11;22(3):1801–16. doi: 10.1021/acs.cgd.1c01381 (PMC9097456; doi:10.1021/acs.cgd.1c01381)
Supplement: Supplementary file 1 — cg1c01381_si_001.pdf [file cg1c01381_si_001.pdf]

Supplementary Information for:

Packing preferences of chalcones: a model conjugated pharmaceutical scaffold.

*Louise S. Price, Sarah L. Price**

Department of Chemistry, University College London, 20 Gordon Street, London WC1H 0AJ, UK.

Table of Contents

S1. Naming convention 2
S2. Full details of the CSD search 3
S3. Structures in the Experimental Set. 4
S3.1 Summary of frequency of substituents 8
S3.2 Summary of number of independent molecules for crystal structures in various space groups. 8
S3.3 Investigating conformational polymorphs 9
S4. Further conformational analysis 9
S5. Full details of the Crystal Structure Prediction (CSP) 9
S5.1 Search method 9
S5.2 Structure refinement 10
S5.3 Symmetry reduction 10
S5.4 Removal of approximately duplicate structures 11
S6. Coordination Ellipsoid 11
S7. Analysis with the CCDC Python API 12
S7.1 Method 12
S7.1.1 Molecular parameters 12
S7.1.2 Intermolecular parameters 12
S7.1.3 Higher Z' structures 13
S7.2 Python scripts 14
S7.2.1 Analyzing crystal structures for molecular parameters 14
S7.2.2 Analyzing crystal structures for intermolecular parameters 14
S7.3 Additional Results 15
S7.3.1 Testing Assumptions 15
S7.3.2 Variation of conformation with substituent 17
S7.3.3 Variation of conformation with Hammett Parameter 18
S7.3.4 Variation of packing efficiency with conformation 19
S7.3.5 The relationship between molecules in van der Waals contact and inversion and translation related molecules within the coordination ellipsoid 19
S7.3.6 Close contacts in the Experimental and CSP Sets 21
S7.3.7 Highly populated areas of the S1/S2/S3 charts 24
S8. Common 1-D motifs 31
S8.1.1 Alternative views of the comparison between $TrSS_{twist}$ and $TrSS_{plan}$ motifs 32
S9. Mercury's Crystal Packing Similarity and XPac analysis 33

The molecular diagram of the unsubstituted chalcone is given in Figure S1.

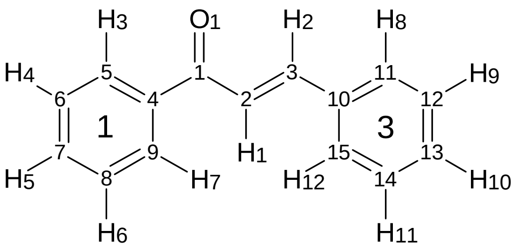

Figure S1. The molecular diagram of the unsubstituted chalcone, with the numbering used in this work. The large numbers show the 1-ring and 3-ring.

S1. Naming convention

The chalcones have all been named according to their substituents and substituent positions. The code is XiYj, where X (alphabetic) and Y (numeric) denote the types of substituents on the 1-ring and 3-ring respectively, and i and j denote the positions of the substituents on the 1-ring and 3-ring, respectively. An example is Gm8p, which denotes a dimethylamino group (“G”) in the meta position (“m”) on the 1-ring and a nitro group (“8”) in the para position (“p”) on the 3-ring.

Table S1. The full list of substituents covered in this work.

Functional group	1-ring	3-ring	Functional group	1-ring	3-ring
H	A	1	SMe	S	19
F	B	2	Ph	T	20
Cl	C	3	OEt	U	21
Br	D	4	piperidine	V	22
I	E	5	$OC(=O)C(=CH_2)Me$	W	23
Me	F	6	$O(CH_2)_4Br$	X	24
NMe_2	G	7	iPr		26
NO_2	H	8	CF_3		27
OMe	I	9	OCF_3		28
OH	J	10	CN		29
NH_2	M	13	$O(CH_2)_4Me$		30
imidazole	O	15	$O(CH_2)_3Me$		31
NEt_2	Q	17	C≡CH		32

S2. Full details of the CSD search

The 2019 Cambridge Structural Database with Feb 2019, May 2019 and Aug 2019 updates was searched for all structures with the following criteria:

- The fragment in Figure S1, including hydrogens on C2 and C3 (but not the other hydrogens).
- Elements H, C and O were required to be present, and I was the heaviest permitted element.
- 3-D coordinates were available, there were no ions, and only organics were included (i.e. no organometallics).

The 842 structures were then viewed and manually sorted to remove:

- Multicomponent systems (with the exception of Cp19p, which is a Cl/Br solid solution).
- Any structures where one substituent was bound to two ring atoms (such as a naphthalene group).
- Any structures where one substituent contained more than 6 non-hydrogen atoms.
- Any zwitterionic species.

This left 470 structures, of which 238 had a maximum of 1 substituent per ring. Structures that were redeterminations of the same solution were removed, retaining the structure with the lowest R factor and/or temperature. Including the 30 novel structures crystallized by the Bristol or Gonzaga teams, this generated a set of 232 structures for analysis.

Some of the structures needed to be edited in order to analyze them with the Python API as this requires whole molecules, with defined hydrogen positions. Hydrogen atoms were added to Ho4p using the edit structure function in Mercury. In the case of A-1--Pbcn (the molecule is disordered with 12% of the molecules having the C=O and C-H groups swapped), the minor component was removed. For Cp19p and Cp3p+Dp4p_disorder, where chloro/bromo disorder was evident, no action was needed as these atoms were not included in the analysis. Cp7p-s has disorder in the orientation of the 3-ring, so two ordered models were used in the analysis.

S3. Structures in the Experimental Set.

Table S2. Full details of structures included in the Experimental Set.

CSD REFCODE / CCDC deposition number	Code	Space group	Z'	Year	1-ring substituent			3-ring substituent			Ref
					ortho	meta	para	ortho	meta	para	
2025587	Gm1-	C2/c	1	2020		NMe2					
2028980	Qp8p	P21/c	1	2020			NEt2			NO2	
2029052	Qp8m	C2/c	1	2020			NEt2		NO2		
2029583	Ep5o	P-1	1	2020			I	I			
2029624	Eo5o	P21/n	1	2020	I			I			
2029648	Eo5p	P21/c	1	2020	I					I	
2029677	Ep4p	Pcc2	1	2020			I			Br	
2029733	Bo4o	P21/c	1	2020	F			Br			
2029763	Cp3m	P21/c	2	2020			Cl		Cl		
2029828	Dm4p	P2/n	1	2020		Br				Br	
2038993	Gm4p	P21/n	1	2020		NMe2				Br	
2050676	A-8p	P1	1	2020						NO2	
2050705	Gp1-	Pbca	1	2020			NMe2				
2050907	Bp5m	P21/n	1	2020			F		I		
2050908	Co4p	C2/c	1	2020	Cl			Br			
2050909	Do5p	P21/c	1	2020	Br					I	
2054068	Ip3p	Pbca	1	2020			OMe			Cl	
AJUFAO	Dm19p	Pbca	1	2011		Br				SMe	1
AMIWEA	Cm26p	P21/c	1	2016		Cl				iPr	2
ARUGUR	Ip27p	P21/c	1	2017			OMe			CF3	3
BRCHAL	Dp1-	P21/c	2	1973			Br				4
BRNICH	Ho4p	P21/c	1	1970	NO2					Br	5
BUDXOO	Hp1-	P21/c	1	2009			NO2				6
BUDYOP	A-2p-I	Cc	1	2009						F	7
BUDYOP01	A-2p-II	P21/c	1	2013						F	8
BZYACO	A-1--Pbc21	Pbc21	1	1970							9
BZYACO03	A-1--Pbcn	Pbcn	1	2006							10
CAGZUI	Mo10p	P21/c	5	2016	NH2					OH	11
CAMFIH	Dp13p-o	Fdd2	1	2011			Br			NH2	12
CAMFIH01	Dp13p-t	P-1	4	2016			Br			NH2	13
CAMFUT	Cp13p	Fdd2	1	2011			Cl			NH2	14
CERYAA01	A-6p-II	P21	3	2008						Me	15
CERYAA02	A-6p-I	P21/n	1	2008						Me	15
CERYAA03	A-6p-IV	C2/c	1	2008						Me	15
CICLUW	Dm1-	P-1	1	2007		Br					16
CICSAJ	Dp9m	P-1	2	2007			Br		OMe		17
CIQFAK	A-3o	Pca21	1	2007				Cl			18
CIQFEO	Sp3p	P21/c	1	2007			SMe			Cl	19
CNCHAL	Ho3m	P21/c	1	1970	NO2				Cl		5
DEGCAU	A-4m	P21/c	1	2006					Br		20
DEGCEY	Hp9p	P212121	1	2006			NO2			OMe	21
DMCHAL	Fp6p	P212121	1	1974			Me			Me	22
DUMMAA	Cp17p	P21/c	2	2010			Cl			NEt2	23
DUSVUJ	Dp9o	P21/n	1	2010			Br	OMe			24
DUSWAQ	Vp9p	P-1	1	2010			piperidine			OMe	25
DUSZEZ	Gp8m	P21/c	1	2020			NMe2		NO2		26
DUSZID	Hm7m	P21/n	1	2020		NO2			NMe2		26
DUSZOJ	Hm1-	P21/c	2	2020		NO2					26
DUZQIB	Gm8p-R	P21/n	1	2020		NMe2				NO2	27
DUZQIB03	Gm8p-B	P21/c	1	2020		NMe2				NO2	27
DUZQOH02	Gm8m-O	P-1	1	2020		NMe2			NO2		27
DUZQOH03	Gm8m-Y	P21/c	1	2020		NMe2			NO2		27
EVOCOJ	Mp3m	P21	1	2017			NH2		Cl		28
FAQFOS	Jo6p	C2/c	1	1999	OH					Me	29
FASKES	Dp28p	Pbca	1	2017			Br			OCF3	30
FASRUP	Hp6o	Pca21	1	2015			NO2	Me			31
FEDLUW	Cp10p	P212121	1	2005			Cl			OH	32

FEDSOX01	Jp9p	Pbca	1	2018			OH			OMe	33
FEDSUD	Dp10m	P21/c	1	2005			Br		OH		34
FINHIU02	Fp9p	P21/c	1	2017			Me			OMe	35
FIWHAV	Ip10m	P-1	2	2005			OMe		OH		36
GASBEK	Dm2m	P21/n	1	2017		Br			F		37
GAVBEL	Cp3p	P21/c	1	2005			Cl			Cl	38
GEJJUB	Cp4p	P21/c	1	2006			Cl			Br	39
GOFWUU	Jp3o	Pc	2	2007			OH	Cl			40
GOKPED	Cp26p	P21/c	1	2014			Cl			iPr	41
HAGZOH	Jo10p	P21	2	2013	OH					OH	42
HESFER	Ip3o	Pbca	1	2006			OMe	Cl			43
HESGES	Dp8p	Pna21	1	2006			Br			NO2	44
HIVPOR	Jo10m	P21/c	1	1999	OH				OH		45
HOKLAU02	A-7p	P21/c	1	2006						NMe2	46
HOPBEV	Jo3o	P212121	2	2014	OH			Cl			47
HORDUN01	Jo10o	C2/c	1	2001	OH			OH			48
HUFZAK	Cm1-	P-1	1	2010		Cl					49
HUFZEO	Cm4p	P-1	1	2010		Cl				Br	50
HUGCIW	Fp7p	P212121	1	2010			Me			NMe2	51
HURXAT	Wp4m	Pna21	1	2003			OC(=O)C(=CH2)Me		Br		52
IDIPAM	Jp1-	Pbca	1	2002			OH				53
IDUPAZ	Bp1-	P-1	1	2006			F				54
IFUWIQ	Sp2p	P21/c	1	2008			SMe			F	55
IGAPAI	Fp4m	P-1	1	2008			Me		Br		56
IJULUV	Bp7p	P21/c	1	2011			F			NMe2	57
IKOFAR	Jo4p-P21	P21	1	2016	OH					Br	58
IKOFAR01	Jo4p-P21/c	P21/c	1	2017	OH					Br	59
ILIMEV	A-10m-I	P2/n	1	2003					OH		60
ILIMEV01	A-10m-III	Pbca	1	2012					OH		61
ILIMEV02	A-10m-II	Pca21	1	2012					OH		61
IWALAV	Dp5p	P21/c	1	2017			Br			I	62
IZEFOI	Dp6p	P21/c	1	2004			Br			Me	63
JAXTEI	Mp4m	P21	1	2005			NH2		Br		64
JEJCAF	Mo3p	P212121	2	2016	NH2					Cl	65
JOFKAR	Dm21p	P21	1	2008		Br				OEt	66
KIKCEN	Fp8p	Pc	2	2007			Me			NO2	67
KIKKOF	Dp19p	Cc	1	2007			Br			SMe	68
KIKQOL	Fp2p	P21/c	1	2007			Me			F	69
KOFGUI	Dp3o	Pna21	1	2008			Br	Cl			70
KOGQEF	Dp3m	P-1	2	2019			Br		Cl		71
KOGQIJ	Dp2o	P21/c	1	2019			Br	F			71
KORROY	Ip4p	Pc	1	1992			OMe			Br	72
KOTSER02	Ip1-	Pbca	1	2018			OMe				73
LANRID	Bp3p	P-1	1	2012			F			Cl	74
LEBGUU	A-3p	P21/c	1	1994						Cl	75
LEHROG	Dp4p	P21/c	1	2006			Br			Br	76
LEPYIP	Dp3p	P21/c	1	2006			Br			Cl	77
LERXOW	Bo29p	P-1	1	2006	F					CN	78
LIKDEO	Jo9o	P21/n	1	1995	OH			OMe			79
LOBVEE	Cp6p	P21/c	1	2008			Cl			Me	80
LUHCAT	Cp15p	P21/n	1	2009			Cl			imidazole	81
LUHCEX	Bp15p	P21/c	1	2009			F			imidazole	81
MAMLEU	Jm8p	P-1	3	2016		OH				NO2	82
MEGYON01	Cp9p-o	Pna21	1	2006			Cl			OMe	83
MEGYON02	Cp9p-m	P21/c	1	2010			Cl			OMe	84
MEHGIQ	Hp4p	P-1	2	2006			NO2			Br	85
MENCOY	Dp9p-P21/c	P21/c	2	2006			Br			OMe	86
MENCOY01	Dp9p-Pca21	Pca21	1	2017			Br			OMe	87
MIKMUR	Jo2p	P212121	1	2018	OH					F	88
MIYCAZ	Bp6p	P21/c	1	2008			F			Me	89
MOBNOJ	Fm8m	P21/n	1	2019		Me			NO2		90
MOBZAC02	A-9p	P21	1	2018						OMe	91
MOGNON01	Dp7p	P21/a	2	2017			Br			NMe2	87
NAMXIL	Ip7p	P21/c	1	2017			OMe			NMe2	92

NAWCEU	Qp29p	P21/c	1	2005			NEt2			CN	93
NEQVAH	Hp19p	Aba2	1	2006			NO2			SMe	94
NIFDOW	Hm7p	P-1	1	2007		NO2				NMe2	95
NURCIN	Dp2p	P21/n	1	2010			Br			F	96
NURFIQ	Mp27o	P21/c	1	2010			NH2	CF3			97
OBIYUW	Dm2p	P-1	1	2017		Br				F	98
OBIZAD	Hm2p	P21/n	2	2017		NO2				F	98
ODEDEH	Dp4m	P21/c	1	2006			Br		Br		99
OTAMAA	Ep2o	P21/c	1	2021			I	F			100
OTAMEE	Ep2m	P-1	2	2021			I		F		100
OTAMII	Ep8p	P21/c	2	2021			I			NO2	100
OZUMUT	Cp19p	Cc	1	2016			Cl/Br			SMe	101
PAQJEZ	Hp6m	P21/c	1	2017			NO2		Me		102
PAQJOJ	Dp27p	P21/c	1	2017			Br			CF3	103
PEFNAS	Up6p	P21/c	1	2017			OEt			Me	104
PELROO	Bp9p	P212121	1	2006			F			OMe	105
PICGEP	Hm10p	P21/c	1	2013		NO2				OH	106
PICGIT	Hm4o	P21/n	1	2013		NO2		Br			106
PIRDEB	Fp3p	P21	1	2013			Me			Cl	107
PIVJIQ	Dp2m	P-1	2	2019			Br		F		108
PIVJUC	Hp2p	P21/c	1	2019			NO2			F	109
POWWII	Cp4m	P21/n	1	2009			Cl		Br		110
PUGVAP	Dm20p	Pca21	1	2009		Br				Ph	111
PUQSOJ	Cp1-	P-1	1	1998			Cl				112
PUQSUP	Fp1--m	C2/c	1	1998			Me				112
PUQSUP01	Fp1--o	Pbca	1	2018			Me				113
QECRAT	Bp2o	P21/c	1	2012			F	F			114
QEDHOY	A-27p	P21/c	1	2012						CF3	115
QEMCER	Ip10p	Pbca	1	2006			OMe			OH	116
QEMCUH	Cp2p	P21/n	1	2006			Cl			F	117
QERNUX	Mp3p	P21/c	1	2006			NH2			Cl	118
QEXLAH	Jo3p	P21/c	1	2007	OH					Cl	119
QOFJUR	Dp21p	P21	1	2008			Br			OEt	120
QOQKAK	Mp7p	P212121	1	2014			NH2			NMe2	121
QUYXOY	Do3p	P21/c	1	2010	Br					Cl	122
QUZCOE	Do9p	P21	1	2010	Br					OMe	123
QUZKUS	Do4p	P21/c	1	2010	Br					Br	124
REPXES	Jo1-	P-1	2	2018	OH						125
RIPDOM	Bp4p-P21/c	P21/c	1	2018			F			Br	126
RIPDOM01	Bp4p-P2/n	P2/n	2	2019			F			Br	127
RIYRAT	Hp6p	P-1	2	2008			NO2			Me	128
RIYRIB	Hp3o	C2/c	1	2008			NO2	Cl			129
ROGBEV	Mp3o	P21/c	1	2008			NH2	Cl			130
ROVHAN	A-21o	P41212	1	2014				OEt			131
RUCKIM	Ep5m	P21/c	1	2019			I		I		132
RUFGOP	Cm3p	P-1	1	2009		Cl				Cl	133
RUGRER	Ho9p	P21/c	1	2009	NO2					OMe	134
SEQLAB	Jp6p	Pcab	1	1998			OH			Me	135
SEZSIA	Ip20p	Cc	1	2007			OMe			Ph	136
SEZSOG	A-20p	P21/n	1	2007						Ph	137
SEZSUM	Dp20p	Cc	1	2007			Br			Ph	138
SEZTAT	Cp20p	Cc	1	2007			Cl			Ph	139
SOGCOH	Sp1-	P212121	1	2008			SMe				140
SOYLEY	A-30p	P21/c	2	2009						O(CH2)4Me	141
SUHZUR	Ip2o	Pbca	1	2009			OMe	F			142
SUZPIN	Jo21p	P-1	1	2010	OH					OEt	143
TADPEX	Cp5p	P21/c	1	2020			Cl			I	144
TAMMUS	Dp8m	P21/n	1	2017			Br		NO2		87
TARCIY	A-4p	Cc	1	1992						Br	145
TERTAM	Cp31p	P-1	1	2006			Cl			O(CH2)3Me	146
TIFWOX	Mo8p	P21/n	1	2018	NH2					NO2	147
TIGSUA	Bp8p	P21/n	1	2018			F			NO2	148
TIHHEY	Jm2p	P21/n	1	2007		OH				F	149
TIHQAD	Jm6p	P21/n	1	2007		OH				Me	150

TIHTOU	Jm3p	P21/c	1	2007		OH				Cl	151
TULQUM	A-10o	P21/n	1	1996				OH			152
TUPFOA	Ip3m	P21	1	2010			OMe		Cl		153
TUPTEE	Ho2p	P21/n	1	2010	NO2					F	154
UDUSES	Hm3o	P21/n	1	2007		NO2		Cl			155
UNOXAX	Jp3p	P21/n	1	2011			OH			Cl	156
UVUSOV	Jo32p	P-1	2	2017	OH					C≡CH	59
VAGNEX02	A-10p	P212121	1	2012						OH	157
VEHRIM	Bp21p	P-1	2	2017			F			OEt	158
VIDDES	Ip9m	P21	1	2007			OMe		OMe		159
VIDFEU	Dm3p	P-1	1	2007		Br				Cl	160
VIDJIC	Dm7p	Pbca	1	2007		Br				NMe2	161
VIVJIU	A-27m	P-1	1	2007					CF3		162
VOQXUV	Cp7p-s	P21/c	1	2009			Cl			NMe2	163
VOQXUV01	Cp7p-b	P21/c	2	2013			Cl			NMe2	164
VUTFEV02	Ip9p	P212121	1	2018			OMe			OMe	165
WEFMIE	Hp10m	P21/c	1	2006			NO2		OH		166
WINMAI	Jo19p	P-1	1	2007	OH					SMe	167
WOBBUL	Cp3o	Pbca	1	2008			Cl	Cl			168
WOTRAA	Tp8m	C2/c	1	2015			Ph		NO2		169
XALQAF	Dm8p	P21	1	2017		Br				NO2	170
XEFLUS	Xp1-	P21/c	1	2017			O(CH2)4Br				171
XEFMAZ	Xp9p	P21/c	1	2017			O(CH2)4Br			OMe	171
XEXXUW	Bp26p	P21/n	1	2018			F			iPr	172
XIGQEK01	Jo9p	Pca21	1	2007	OH					OMe	173
XISGAI	Hm6p-t	P-1	2	2008		NO2				Me	174
XISGAI01	Hm6p-m	P21	1	2019		NO2				Me	175
XOFFEF	Tp6o	P-1	1	2014			Ph	Me			176
XOHHUA	Up9p	P21/n	1	2018			OEt			OMe	177
XOHJAI	Up3m	P-1	2	2018			OEt		Cl		177
XOHJEM	Up3o	P-1	2	2018			OEt	Cl			177
XORXAD02	Jo7p	P21/c	1	2014	OH					NMe2	178
YAWFUZ	Vp8m	Pbca	1	2012			piperidine		NO2		179
YESDUW	Ip4m	P21	1	2006			OMe		Br		180
YESMEP	Cp19p	Cc	1	2006			Cl			SMe	181
YEYFAK	Hp9o	Pbca	1	2007			NO2	OMe			182
YEYFEO	Hm9p	P21/c	2	2007		NO2				OMe	183
YICLIG	Cp21p	P21	1	2007			Cl			OEt	184
YIFVOZ	Bp20p	P21	2	2007			F			Ph	185
YISKAR02	Ip21p	Pna21	1	2017			OMe			OEt	186
YODKEI	Im3o	P-1	1	2008		OMe		Cl			187
YOKTEX	Wp4o	P-1	1	2002			OC(=O)C(=CH2)Me	Br			188
YOTYIQ	Ip2p	Pbca	1	2009			OMe			F	189
YUQFIA	Jp7p	P21	2	2010			OH			NMe2	190
ZIJQEQ	Cp8p	Pna21	1	2013			Cl			NO2	191
*	C/Dp3/4p	P21/c	1	*			Cl/Br			Cl/Br	

* is a structure that could not be refined to a publishable standard.

S3.1 Summary of frequency of substituents

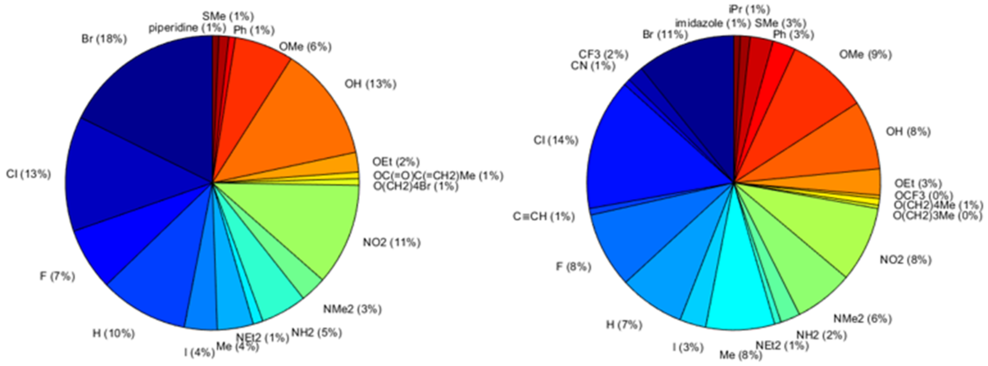

Figure S2. Breakdown of the substituents on (left) the 1-ring and (right) the 3-ring.

S3.2 Summary of number of independent molecules for crystal structures in various space groups.

Table S3. Breakdown of number of independent molecules in the crystal structures within each space group.

	Z'					
**Space group**	**1**	**2**	**3**	**4**	**5**	**Total**
Aba2	1					1
C2/c	8	1				9
Cc	8					8
Fdd2	2					2
P-1	22	13	1	1		37
P1	1					1
P2/n	2	1				3
P21	14	3	1			18
P21/a		1				1
P21/c	65	9			1	75
P21/n	27	1				28
P212121	10	2				12
P41212	1					1
Pbc21	1					1
Pbca	17					17
Pbcn	1					1
Pc	1	2				3
Pca21	6					6
Pcab	1					1
Pcc2	1					1
Pna21	6					6
**Total**	**195**	**33**	**2**	**1**	**1**	**232**

S3.3 Investigating conformational polymorphs

Strictly, conformational polymorphs have molecules that, when the geometry is optimized, are different conformations.

Table S4. Conformational differences in molecules whose crystal structures show conformational polymorphism. The planar1 angle in the CSD crystal structure and the same angle and conformational (and relative conformational) energy calculated at the PBE0/6-31G(d,p) and MP2(6-31G(d,p) levels of theory are given. The angles are also given for the molecule without substituents, labelled A-1-.

Structure	Exp planar1	Molecule	PBE0 opt planar1	PBE0 opt E / H (relative kJ mol^{-1})	MP2 opt planar1	MP2 opt E / H (relative kJ mol^{-1})
A-6p-I	50.20°	A-6p	16.226°	-692.557649025 (0)	46.040°	-691.21779322633 (0.186)
		A-1-	18.368°	-653.285089668 (<0.001)	47.552°	-652.03038368825 (0.183)
A-6p-IV	-2.32°	A-6p	16.226°	-692.557649024 (<0.001)	14.501°	-691.21786414699 (0)
		A-1-	18.365°	-653.285089667 (< 0.001)	12.965°	-652.03045343663 (0)
Cp9p-o[1]	17.66°	Cp9p	17.645°	-1227.12487703 (0)	18.707°	-1225.2601792988 (0)
		A-1-	18.346°	-653.285089661 (< 0.001)	12.929°	-652.03045342875 (<0.001)
Cp9p-m[1]	-52.94°	Cp9p	-16.128°	-1227.12446287 (1.087)	-45.220°	-1225.2598091760 (0.972)
		A-1-	-18.358°	-653.285089672 (0)	-47.566°	-652.03038367282 (0.183)
Bp4p-P21/c	12.36°	Bp4p	-11.913°	-3323.20942861 (0)	-13.135°	-3320.4553554575 (0)
		A-1-	-18.355°	-653.285089668 (<0.001)	-12.956°	-652.03045343027 (<0.001)
Bp4p-P2/n-1	45.19°	Bp4p	11.895	-3323.20942858 (<0.001)	13.129°	-3320.4553554274 (<0.001)
		A-1-	18.347°	-653.285089669 (<0.001)	12.933°	-652.03045341663 (<0.001)
Bp4p-P2/n-2	46.94°	Bp4p	11.923°	-3323.20942860 (<0.001)	46.144°	-3320.4553069767 (0.127)
		A-1-	18.370°	-653.285089669 (<0.001)	47.551°	-652.03038369873 (0.183)

[1] Cp9p-o and Cp9p-m have different conformations of the methoxy group, so are strictly conformational polymorphs, but not in the context of the chalcone conformation.

S4. Further conformational analysis

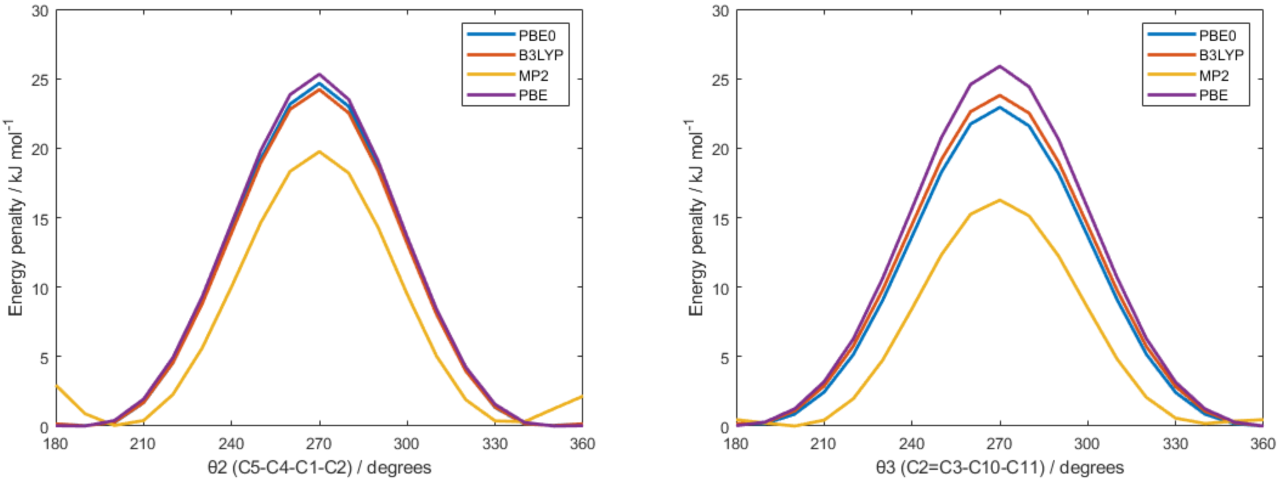

Figure S3. Relaxed torsion angle scans for the unsubstituted chalcone molecule in the Region A conformation about (left) θ_2 and (right) θ_3 at the PBE0, B3LYP, MP2, and PBE levels of theory with the 6-31G(d,p) basis set.

S5. Full details of the Crystal Structure Prediction (CSP)

S5.1 Search method

CrystalPredictor_2.2[192] was used to search for plausible crystal structures. The search was divided into two parts; Region A corresponding to the conformation illustrated in Figure S1, and Region B, the alternative conformation that corresponded to a high energy local minimum (see Figure 5 of the main paper). In Region A, the central angle (θ_1, C4-C1-C2=C3) was centred on 0°, with the full range allowed given in Table S5. CrystalPredictor uses libraries of LAMs for the conformation, conformational energy and point charge description of the molecule, as the molecule varies through the conformational ranges defined in Table S5. The library of LAM points was constructed by carrying out a constrained minimization of the molecule in GAUSSIAN at the PBE0 level of theory with the 6-31G(d,p) basis set. Point charges at each expansion point were also calculated at the PBE0 level of theory with the 6-31G(d,p) basis

set. The search was carried out in 61 space groups, namely *P*1, *P*-1, $P2_1$, $P2_1/c$, $P2_12_12$, $P2_12_12_1$, $Pna2_1$, $Pca2_1$, *Pbca*, *Pbcn*, *C*2/*c*, *Cc*, *C*2, *Pc*, *Cm*, $P2_1/m$, *C*2/*m*, *P*2/*c*, $C222_1$, $Pmn2_1$, $Cmc2_1$, *Aba*2, *Fdd*2, *Iba*2, *Pnna*, *Pccn*, *Pbcm*, *Pnnm*, *Pmmn*, *Pnma*, *Cmcm*, *Cmca*, *Fddd*, *Ibam*, $P4_1$, $P4_3$, *I*-4, *P*4/*n*, $P4_2/n$, *I*4/*m*, $I4_1/a$, $P4_12_12$, $P4_32_12$, $P\text{-}42_1c$, *I*-42*d*, $P3_1$, $P3_2$, *R*3, *P*-3, *R*-3, $P3_121$, $P3_221$, *R*3*c*, *R*-3*c*, $P6_1$, $P6_3$, $P6_3/m$, $P2_13$, *Pa*-3, $P222_1$, and *Pba*2. Parameters from the FIT potential were used to model the repulsion-dispersion interactions. 1,000,000 structures were generated and minimized in Region A, with duplicates and structures whose total lattice energy was more than 15 kJ mol^{-1} higher than the lowest energy structure rejected, resulting in 18,212 unique Region A structures.

In Region B, only the PBE0/6-31G(d,p) gas phase optimized molecular structure was used for the search because this higher energy local conformation was in a sharply-defined energy well. The same energy model was used as for Region A, and the same 61 space groups. 100,000 structures were generated and minimized in Region B, with duplicates and structures whose total lattice energy was more than 15 kJ mol^{-1} higher than the most stable in this Region rejected, resulting in 2,498 unique Region B structures.

Table S5. The ranges of the independent degrees of freedom allowed for the search in Region A and the values of these degrees of freedom in the rigid molecule search in Region B.

Region A	θ_1: C4-C1-C2=C3	θ_2: C5-C4-C1-C2	θ_3: C2=C3-C10-C11
Search range	140-220°	130-230°	130-230°
LAM points	150-210° in 20° steps	140-220° in 20° steps	140-220° in 20° steps
Region B	θ_1 : C4-C1-C2=C3	θ_2 : C4-C1-C2=C3	θ_3 : C4-C1-C2=C3
Optimized value	334.3°	150.6°	175.7°

S5.2 Structure refinement

CrystalOptimizer_2.4.7[193] was used to refine the crystal structures allowing the conformations to change in response to the packing forces. This is a two level optimization, with the intramolecular energy evaluated at the PBE0/6-31G(d,p) level of theory with GAUSSIAN, and the intermolecular energy evaluated with DMACRYS_2.3.0,[194] which makes use of a distributed multipole model of the charge density, up to hexadecapole, on each atom. The charge density was calculated in GAUSSIAN at the PBE0/6-31G(d,p) level of theory, and the multipoles extracted using GDMA_2.2.[195] The isotropic exp-6 repulsion-dispersion parameters were from the FIT potential.[196] The independent degrees of freedom that were allowed to optimize in response to the packing were 5 dihedral angles: C4-C1-C2=C3 (θ_1), C5-C4-C1-C2 (θ_2), C10-C3=C2-C1, C11-C10-C3=C2 (θ_3) and O1=C1-C2=C3 (to not assume planarity of this group), and 6 bond angles: O1=C1-C2, C4-C1-C2, C3=C2-C1, H1-C2-C1, C10-C3=C2, and H2-C3-C2, as shown in Figure S4.

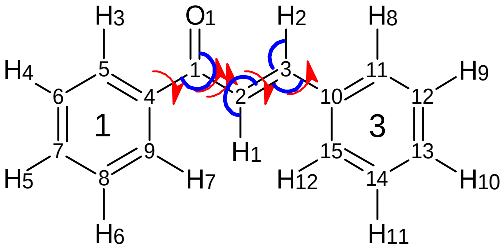

Figure S4. The independent degrees of freedom refined by CrystalOptimizer as influenced by the crystal structure specific intermolecular forces. Dihedral angles (C4-C1-C2=C3 (θ_1), C5-C4-C1-C2 (θ_2), C10-C3=C2-C1, C11-C10-C3=C2 (θ_3) and O1=C1-C2=C3) are shown by red arrows, and bond angles (O1=C1-C2, C4-C1-C2, C3=C2-C1, H1-C2-C1, C10-C3=C2, and H2-C3-C2) are shown by blue curves.

S5.3 Symmetry reduction

Any structures that DMACRYS identified as high energy saddle points between two lower symmetry lower energy structures had the symmetry reduced and were reminimized with CrystalOptimizer in the lower symmetry subgroup. Thus although CrystalPredictor only generated Z'=1 structures, the search dataset includes approximately 10 % Z'>1 structures (410 in Region A and 12 in Region B).

S5.4 Removal of approximately duplicate structures

All pairs of structures with a lattice energy difference less than 2 kJ mol^{-1}, and a density difference of less than 0.1 g cm^{-3} were analyzed to determine whether they were effectively equivalent. Structures were considered effectively equivalent if they were in the same space group, their powder patterns matched with a similarity of better than 0.9, and a 15-molecule coordination sphere with distance tolerance of 20% and angle tolerance of 20° matched with $RMSD_{15}$ < 0.5 Å. If structures were in different space groups, they were deemed effectively equivalent if their powder patterns matched with a similarity of better than 0.9, and a 30-molecule coordination sphere with distance tolerance of 20% and angle tolerance of 20° matched with $RMSD_{30}$ < 0.1 Å. This resulted in 4725 unique structures within 20 kJ mol^{-1} of the global energy minimum, of which 141 were in Region B. The 20 kJ mol^{-1} cutoff was chosen to include Region B structures, knowing that some experimental structures adopted this conformation.

S6. Coordination Ellipsoid

Initially, the coordination ellipsoid for the definition of close contacts was decided by visual inspection of the plots of translation and inversion dimer contacts, which showed a clear cutoff by the absence of any dimers. For the CSP Set, a maximum S1 distance of 4 Å, a maximum S2 distance of 18 Å, and a maximum S3 distance of 8 Å was chosen. These were increased to 5, 25 and 8 Å for dimer contacts of structures in the Experimental Set.

Table S6. Definition of a box containing a single molecule from a sample of the (Z'=1) crystal structures. The reason for selection is given alongside the structure name, and the key molecular angles are given. The atoms at the extremities of the coordinate system applied by NEIGHCRYS, with the x-line along C5-C11 and the y-plane defined by C5, C11, H1, are given, and the total lengths of the principal axes of inertia, including the van der Waals radii, are given in the final column.

Structure	θ_1 / °	θ_2 / °	θ_3 / °	planar1 / °	Axis	Atom	Coordinate / Å	vdW radius / Å	Limit / Å	Length / Å
A9368 (lowest energy conformation)										
	-174.6	-168.8	-179.7	16.6	x	H5	-5.995834	1.2	-7.196	14.6
						H10	6.233045	1.2	7.433	
					y	H11	-2.814481	1.2	-4.014	7.8
						O1	2.144806	1.52	3.765	
					z	H3	-0.36347	1.2	-1.563	3.6
						H6	0.837563	1.2	2.038	
A15148 (most planar molecule)										
	180	-179.9	179.8	0.04	x	H5	-6.017207	1.2	-7.217	14.7
						H10	6.245709	1.2	7.446	
					y	H11	-2.824589	1.2	-4.025	7.7
						O1	2.137913	1.52	3.658	
					z	H3	-0.005390	1.2	-1.205	2.4
						H6	0.004475	1.2	1.204	
A10998 (planar1 closest to 50°)										
	-162.0	-165.1	-159.3	50.0	x	H5	-5.804905	1.2	-7.005	14.4
						H10	6.148389	1.2	7.348	
					y	H11	-2.830966	1.2	-4.031	7.6
						O1	2.015228	1.52	3.535	
					z	H11	-1.011759	1.2	-2.211	5.6
						H6	2.208905	1.2	3.409	
B2027 (lowest energy Region B)										
	-26.0	148.4	174.8	120.6	x	H4	-4.956057	1.2	-6.156	13.4
						H10	6.045054	1.2	7.245	
					y	H11	-2.784394	1.2	-3.984	8.6
						H5	3.396598	1.2	4.597	
					z	H3	-1.251260	1.2	-2.451	6.3
						H6	2.685774	1.2	3.886	

A cross check on the ellipsoid axis lengths was made by comparison with the dimensions of a box that contained the molecule, with side parallel to the three orthogonal principal axes of inertia.[197] Using the functionality in NEIGHCRYS,[194] the input file generator for DMACRYS, an orthogonal coordinate system was set up for a set of

molecules. The x-axis for each molecule was set to be from C5 to C11 and the y-plane was defined by C5, C11 and H1. The atoms with the highest and lowest values in each axis are given in Table S6, and the van der Waals radius added for each atom. This gives three axes for each molecule, which can be used to define the principal axes of inertia, and hence the box size and shape. Table S6 shows that the definition of the coordination ellipsoid chosen for the structures in the CSP Set of S1<=4 Å, S2<=18 Å, S3<=8 Å is reasonable. Pidcock's work notes that the boxes don't align within the crystal structures since the edges are not parallel to the unit cell axes, and hence the molecules readily overlap into neighbouring boxes, hence although the box is a different shape for the molecule with planar1 closest to 50° and for the molecule in Region B, this definition of the ellipsoid will identify most of the molecules in van der Waals contact.

S7. Analysis with the CCDC Python API

S7.1 Method

S7.1.1 Molecular parameters

The CCDC Python API was used to analyze all crystal structures, and automatically measure molecular geometric parameters. The code is given in Section S7.2 and the variable names in the code are used. Both Greek and code names for variables are given when appropriate.

For each crystal structure, the chalcone backbone was identified using the SMART notation

[c]1[c][c][c][c][c]1[CD3](=[OD1])[CH1D2]([H])=[CH1D2][c]2[c][c][c][c][c]2

Within this substructure, the variables listed in Table S7 were measured and recorded.

Table S7. The variables measured for the chalcone molecules.

Variable	Definition	Notes
TOR1=θ_1	C4-C1-C2-C3	
TOR2=θ_2	C5-C4-C1-C2	C9-C4-C1-C2 also measured, and the one closest to 180° stored
TOR3=θ_3	C2=C3-C10-C11	C2=C3-C10-C15 also measured, and the one closest to 180° stored
planar1	C8~C5~C11~C14	Depending on which definitions of TOR2=θ_2 and TOR3=θ_3 were stored, this could have been C6~C9~C11~C14, C8~C5~C15~C12 or C6~C9~C15~C12
C2-CENT	Distance between C2 and the geometric centroid of the 17 atoms in the SMART substructure	
ANG1	H1-C2~C12	H1-C2~C14 stored if C2=C3-C10-C15 was stored

For all angles below -90°, 360° was added so that torsion angles were all in the range -90° to 270°, so similar conformations were grouped together.

In the analysis of the intermolecular geometries of pairs of molecules, it was assumed that C2 was the centre of the molecule and H1-C2~C12 was 90°. The C2-CENT and ANG1 measurements were taken to test the validity of these assumptions.

S7.1.2 Intermolecular parameters

The SMART substructure defined in Section S7.1.1 was identified in two molecules within the crystal structure, and an intermolecular distance between the C2 atoms was measured as D1. Only dimers where this value was below 30 Å for structures in the Experimental Set and below 20 Å in the search structures were considered further.

Between these two molecules, a number of other geometric parameters were measured, as listed in Table S8. Since the H1-C2~C12 angle was found to be virtually 90° for all molecules (in both Region A and Region B; see Figure S6), the distance between the two molecules could be separated into three orthogonal components. These components are parallel to C2-H1 (S3), parallel to C2~C12 (S2) and perpendicular to these two directions (S1) (see Figure 2 in main paper). To measure the displacement perpendicular to H1-C2~C12, the average geometric plane of the 17 atoms in the SMART structure was defined for each molecule, and the separation of these planes measured (S1).

(The separation of a plane can only be defined if the planes are parallel or anti-parallel.) For pairwise contacts where the two molecules are parallel, τ_1 and τ_2 are both 0°, and for pairwise contacts where the two molecules are anti-parallel, τ_1 and τ_2 are both 180°. These are defined as translation and inversion contacts. S2 and S3 were derived from the measured quantities, as listed in Table S8.

Table S8. The geometric parameters, both measured and derived, between two distinct molecules in each crystal structure.

Variable	Definition	Notes
D1	C2···C2′	
A1	C2···C2′~C12′	C2···C2′~C14′ may be stored depending on the definition used for TOR3=θ_3
T1=τ_1	C12~C2···C2′~C12′	C14~C2···C2′~C14′ may be stored depending on the definition used for TOR3=θ_3
A2	C2···C2′-H1′	
T2=τ_2	H1-C2···C2′-H1′	
S1	Distance between average geometric planes of two molecules.	Face-to-face displacement
S2	$\cos(A1) * D1$	End-to-end displacement
S3	$\cos(A2) * D1$	Side-to-side displacement

S7.1.3 Higher Z′ structures

There were a number of crystal structures with Z′>1, some of which had quite different conformations. Table S9 lists the crystal structures with high Z′ (<1) where the conformations are not similar. These occasionally posed a problem for the automated analysis, in matching conformation with S1, S2, S3 parameters.

Table S9. Z′>1 experimental crystal structures where molecular conformations were not the same, i.e. the 15 carbon and 1 oxygen atoms matched in Mercury's Structure Overlay with $RMSD_1$>0.15 Å, unless otherwise stated.

Crystal structure	Z′	Conformations				Comments
		TOR1=θ_1	TOR2=θ_2	TOR3=θ_3	Planar1	
20mec09_pub(Cp3m)	2	150,172	176,174	169,187	-41,-2	One planar, one twisted
CAGZUI(Mo10p)	5	168,169,178, 181,184	161,184,168, 174,186	169,192,187, 176,183	-44,-4,-12, -4,12	Two planar, one twisted, two slightly twisted
CAMFIH01(Dp13p-t)	4	166,173, 173,178	196,199, 159,160	184,182, 186,188	2,10, -23,-15	One planar, three slightly twisted
CERYAA01(A-6p-II)	3	180,186,195	165,203,209	182,205,186	-5,48,44	One planar, two twisted
Ep2m(Ep2m)	2	180,186	173,175	184,172	-4,-4	Different meta substituent position ($RMSD_1$=0.069)
Ep8p(Ep8p)	2	180,187	170,206	198,192	5,44	One planar, one twisted
FIWHAV(Ip10m)	2	183,192	175,171	182,193	5,13	One planar, one slightly twisted
GOFWUU(Jp3o)	2	172,189	168,187	152,191	-43,29	One twisted, one slightly twisted
JEJCAF(Mo3p)	2	181,182	182,205	175,172	-3,18	One planar, one slightly twisted
KOGQEF(Dp3m)	2	167,179	183,187	185,183	-5,6	Different meta substituent position ($RMSD_1$=0.128)
MAMLEU(Jm8p)	3	-3,181,187	119,179,172	190,177,171	146,1,-7	One Region B, two planar
MEHGIQ(Hp4p)	2	174,183	161,165	167,193	-41,1	One planar, one twisted
MOGNON01(Dp7p)	2	161,191	161,173	175,192	-47,6	One planar, one twisted
PIVJIQ(Dp2m)	2	181,185	174,174	180,174	-3,-5	Different meta substituent position ($RMSD_1$=0.046)
RIYRAT(Hp6p)	2	175,176	160,195	167,168	-42,-1	One planar, one twisted
VEHRIM(Bp21p)	2	177,189	191,208	173,191	-1,49	One planar, one twisted
VOQXUV01(Cp7p-b)	2	191,198	172,199	191,186	5,48	One planar, one twisted
YUQFIA(Jp7p)	2	192,195	162,201	186,194	-4,48	One planar, one twisted

S7.2 Python scripts

The python scripts used in this work are given below. Not all measured parameters are reported in this work. Auxiliary scripts were used to determine which of the multiple definitions for some angles was the correct one to choose.

S7.2.1 Analyzing crystal structures for molecular parameters

```
from ccdc import io
crystal_reader = io.CrystalReader('lowest.res')
cryst = crystal_reader[0]
cryst.assign_bonds()
#print (cryst.molecule.smiles)
from ccdc.search import SMARTSSubstructure
chalcone = SMARTSSubstructure("[c]1[c][c][c][c][c]1[CD3](=[OD1])[CH1D2]([H])=[CH1D2][c]2[c][c][c][c][c]2")
from ccdc.search import SubstructureSearch
from ccdc.descriptors import MolecularDescriptors as MD, GeometricDescriptors as GD
substructure_search = SubstructureSearch()
sub1 = substructure_search.add_substructure(chalcone)
substructure_search.add_torsion_angle_measurement('TOR1', (sub1, 5),(sub1, 6), (sub1, 8), (sub1, 10))
substructure_search.add_torsion_angle_measurement('TOR2', (sub1, 4),(sub1, 5), (sub1, 6), (sub1, 8))
substructure_search.add_torsion_angle_measurement('TOR2a', (sub1, 0),(sub1, 5), (sub1, 6), (sub1, 8))
substructure_search.add_torsion_angle_measurement('TOR3', (sub1, 8),(sub1, 10), (sub1, 11), (sub1, 12))
substructure_search.add_torsion_angle_measurement('TOR3a', (sub1, 8),(sub1, 10), (sub1, 11), (sub1, 16))
substructure_search.add_plane('PLANE1', (sub1, 0), (sub1, 1), (sub1, 2), (sub1, 3), (sub1, 4), (sub1, 5),
(sub1, 6), (sub1, 7), (sub1, 8), (sub1, 9), (sub1, 10), (sub1, 11), (sub1, 12), (sub1, 13), (sub1, 14),
(sub1, 15), (sub1, 16))
substructure_search.add_centroid('CENT1', (sub1, 0), (sub1, 1), (sub1, 2), (sub1, 3), (sub1, 4), (sub1, 5),
(sub1, 6), (sub1, 7), (sub1, 8), (sub1, 9), (sub1, 10), (sub1, 11), (sub1, 12), (sub1, 13), (sub1, 14),
(sub1, 15), (sub1, 16))
substructure_search.add_distance_measurement('C2-CENT', 'CENT1', (sub1, 8))
substructure_search.add_angle_measurement('ANG1', (sub1, 9), (sub1, 8), (sub1, 13))
substructure_search.add_angle_measurement('ANG1a', (sub1, 9), (sub1, 8), (sub1, 15))
substructure_search.add_torsion_angle_measurement('planar1', (sub1, 1),(sub1, 4), (sub1, 12), (sub1, 15))
substructure_search.add_torsion_angle_measurement('planar1a', (sub1, 1),(sub1, 4), (sub1, 16), (sub1, 13))
substructure_search.add_torsion_angle_measurement('planar1b', (sub1, 3),(sub1, 0), (sub1, 12), (sub1, 15))
substructure_search.add_torsion_angle_measurement('planar1c', (sub1, 3),(sub1, 0), (sub1, 16), (sub1, 13))

hits = substructure_search.search(cryst)
for h in hits:
    cell_a = h.crystal.cell_lengths[0]
    cell_b = h.crystal.cell_lengths[1]
    cell_c = h.crystal.cell_lengths[2]
    cell_alpha = h.crystal.cell_angles[0]
    cell_beta = h.crystal.cell_angles[1]
    cell_gamma = h.crystal.cell_angles[2]
    print ('%.4f ' % cell_a,
           '%.4f ' % cell_b,
           '%.4f ' % cell_c,
           '%.4f ' % cell_alpha,
           '%.4f ' % cell_beta,
           '%.4f ' % cell_gamma,
           '(TOR1, %.4f )' % h.measurements['TOR1'],
           '(TOR2, %.4f )' % h.measurements['TOR2'],
           '(TOR2a, %.4f )' % h.measurements['TOR2a'],
           '(TOR3, %.4f )' % h.measurements['TOR3'],
           '(TOR3a, %.4f )' % h.measurements['TOR3a'],
           '(C2-CENT, %.4f )' % h.measurements['C2-CENT'],
           '(ANG1, %.4f )' % h.measurements['ANG1'],
           '(ANG1a, %.4f )' % h.measurements['ANG1a'],
           '(planar1, %.4f )' % h.measurements['planar1'],
           '(planar1a, %.4f )' % h.measurements['planar1a'],
           '(planar1b, %.4f )' % h.measurements['planar1b'],
           '(planar1c, %.4f )' % h.measurements['planar1c'])
```

S7.2.2 Analyzing crystal structures for intermolecular parameters

```
from ccdc import io
crystal_reader = io.CrystalReader('lowest.res')
cryst = crystal_reader[0]
cryst.assign_bonds()
#print cryst.molecule.smiles
from ccdc.search import SMARTSSubstructure
chalcone = SMARTSSubstructure("[c]1[c][c][c][c][c]1[CD3](=[OD1])[CH1D2]([H])=[CH1D2][c]2[c][c][c][c][c]2")
from ccdc.search import SubstructureSearch
from ccdc.descriptors import MolecularDescriptors as MD, GeometricDescriptors as GD
substructure_search = SubstructureSearch()
sub1 = substructure_search.add_substructure(chalcone)
sub2 = substructure_search.add_substructure(chalcone)
```

```
substructure_search.add_plane('PLANE1', (sub1, 0), (sub1, 1), (sub1, 2), (sub1, 3), (sub1, 4), (sub1, 5),
(sub1, 6), (sub1, 7), (sub1, 8), (sub1, 9), (sub1, 10), (sub1, 11), (sub1, 12), (sub1, 13), (sub1, 14),
(sub1, 15), (sub1, 16))
substructure_search.add_plane('PLANE1B', (sub2, 0), (sub2, 1), (sub2, 2), (sub2, 3), (sub2, 4), (sub2, 5),
(sub2, 6), (sub2, 7), (sub2, 8), (sub2, 9), (sub2, 10), (sub2, 11), (sub2, 12), (sub2, 13), (sub2, 14),(sub2,
15), (sub2, 16))
substructure_search.add_centroid('CENT1', (sub1, 0), (sub1, 1), (sub1, 2), (sub1, 3), (sub1, 4), (sub1, 5),
(sub1, 6), (sub1, 7), (sub1, 8), (sub1, 9), (sub1, 10), (sub1, 11), (sub1, 12), (sub1, 13), (sub1, 14),
(sub1, 15), (sub1, 16))
substructure_search.add_centroid('CENT1B', (sub2, 0), (sub2, 1), (sub2, 2), (sub2, 3), (sub2, 4), (sub2, 5),
(sub2, 6), (sub2, 7), (sub2, 8), (sub2, 9), (sub2, 10), (sub2, 11), (sub2, 12), (sub2, 13), (sub2, 14),
(sub2, 15), (sub2, 16))
substructure_search.add_distance_constraint('D1', (sub1, 8), (sub2, 8), (0, 30), 'Intermolecular')
substructure_search.add_angle_measurement('A1', (sub1, 8), (sub2, 8), (sub2, 13))
substructure_search.add_angle_measurement('A1a', (sub1, 8), (sub2, 8), (sub2, 15))
substructure_search.add_torsion_angle_measurement('T1', (sub1, 13),(sub1, 8), (sub2, 8), (sub2, 13))
substructure_search.add_torsion_angle_measurement('T1a', (sub1, 15),(sub1, 8), (sub2, 8), (sub2, 15))
substructure_search.add_angle_measurement('A2', (sub1, 8), (sub2, 8), (sub2, 9))
substructure_search.add_torsion_angle_measurement('T2', (sub1, 9),(sub1, 8), (sub2, 8), (sub2, 9))

hits = substructure_search.search(cryst)
for h in hits:
    h.measurements['S1'] = h.geometric_objects['PLANE1'].plane_distance(h.geometric_objects['PLANE1B'])
    h.measurements['PLANE1-CENT1B'] =
h.geometric_objects['PLANE1'].point_distance(h.geometric_objects['CENT1B'])
    h.measurements['PLANE1B-CENT1'] =
h.geometric_objects['PLANE1B'].point_distance(h.geometric_objects['CENT1'])
    h.measurements['PLANEANGLE'] = h.geometric_objects['PLANE1'].plane_angle(h.geometric_objects['PLANE1B'])
    print ('(D1, %.4f )' % h.constraints['D1'],
           '(S1, %.4f )' % h.measurements['S1'],
           '(PLANE1-CENT1B, %.4f )' % h.measurements['PLANE1-CENT1B'],
           '(PLANE1B-CENT1, %.4f )' % h.measurements['PLANE1B-CENT1'],
           '(PLANEANGLE, %.4f )' % h.measurements['PLANEANGLE'],
           '(A1, %.4f )' % h.measurements['A1'],
           '(A1a, %.4f )' % h.measurements['A1a'],
           '(T1, %.4f )' % h.measurements['T1'],
           '(T1a, %.4f )' % h.measurements['T1a'],
           '(A2, %.4f )' % h.measurements['A2'],
           '(T2, %.4f )' % h.measurements['T2'])
```

S7.3 Additional Results

S7.3.1 Testing Assumptions

The analysis of the crystal structures with the Python API tools was much simpler using atoms to define angles and distances. This section shows how closely C2 approximates the geometric centroid of the molecule and the extent to which H1-C2 and C2~C12 are orthogonal. The intermolecular C2···C2 distances and the angle this makes with the intramolecular C2~C12 and C2~H1 vectors define the end-to-end and side-to-side displacements S2 and S3 (Table S8).

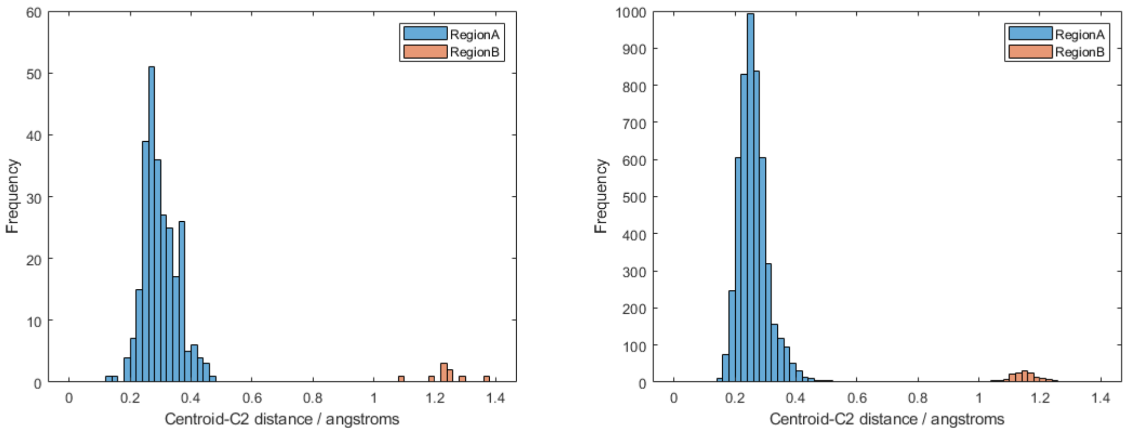

Figure S5. The distance between the geometric centroid of the molecule and the C2 atom in (left) the Experimental Set and (right) the CSP Set. The structures are colored by whether the θ_1 angle is close to 180° (Region A) or close to ±30° (Region B).

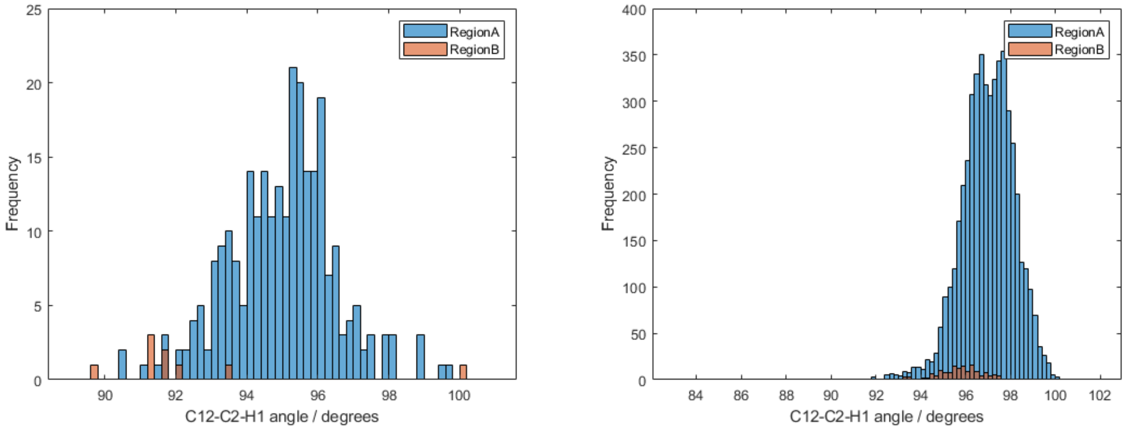

Figure S6. The H1-C2~C12 angle in (left) the Experimental Set and (right) the CSP Set. The structures are colored by whether the θ_1 angle is close to 180° (Region A) or close to ±30° (Region B).

Figure S5 shows that taking C2 as the centre of the molecule is a reasonable approximation (<0.5 Å) for the vast majority of structures in Region A, and C2 is the closest atom to the geometric centroid. For the sparsely populated Region B, the geometric centroid is in the hollow in the molecule but since C2 is only about 1.2 Å from this point, it is likely to be one of the closest atoms.

Figure S6 shows that the H1-C2~C12 angle is between 90 and 100° for all structures in both the Experimental Set and the CSP Set, regardless of the θ_1 angle (Region A/B). While H1-C2~C12 is not exactly 90°, within the translation contacts of the Experimental Set, less than 10% of all contacts (i.e. up to D1=20/30 Å) have $\sqrt{S1^2+S2^2+S3^2}$ deviating more than 10% from D1, and less than 1% of the contacts in the first coordination ellipsoid are outside this deviation. The maximum discrepancy between the geometric mean of the three components of the intermolecular separation, $\sqrt{S1^2+S2^2+S3^2}$ and the measured molecular separation, D1 (C2···C2') is about 20%.

S7.3.2 Variation of conformation with substituent

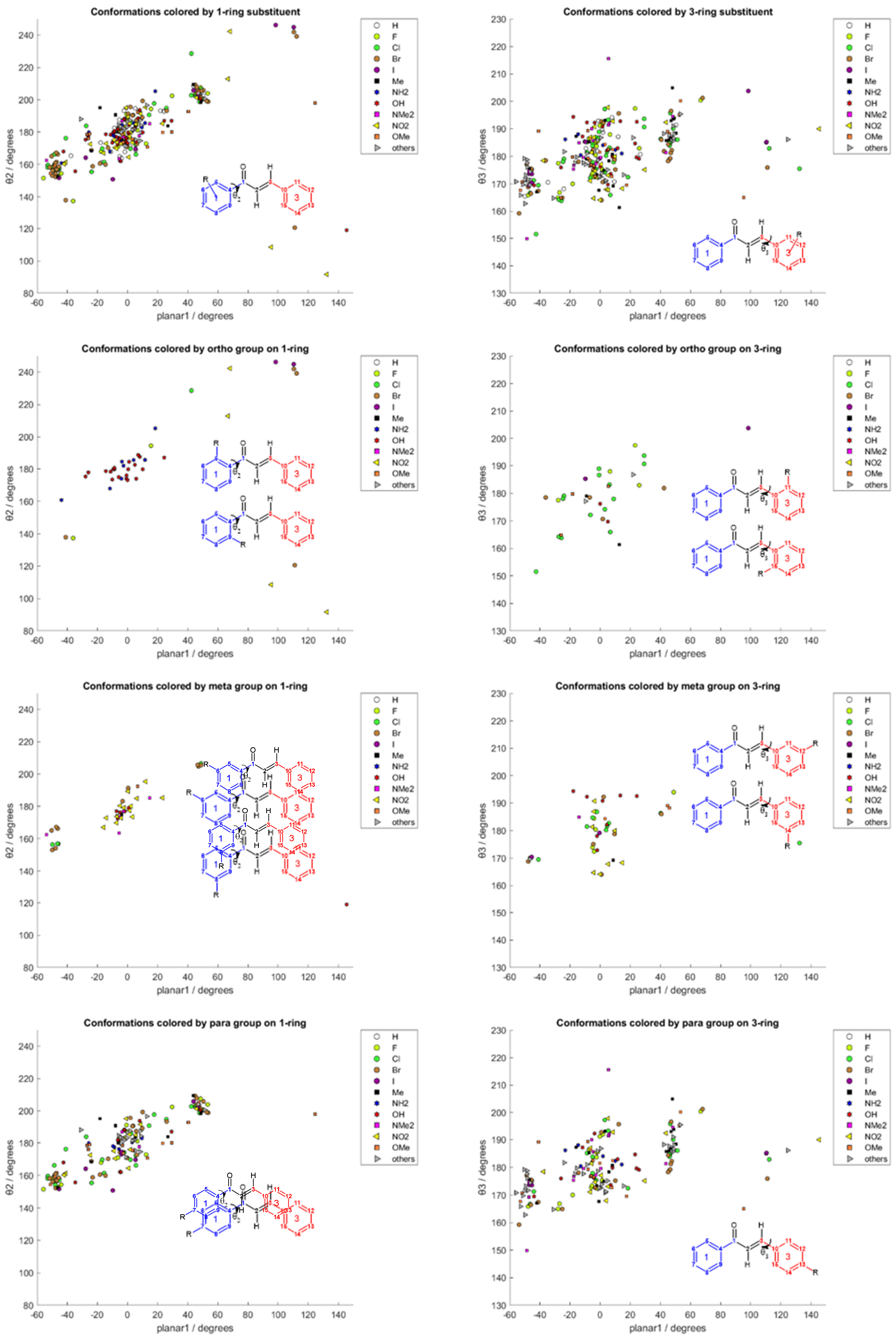

Figure S7. Plots of the conformations of molecules in the Experimental Set colored by the substituent (and position) on each ring. Each plot shows the overall planarity of the molecule (planar1, C8~C5~C11~C14, x-axis) versus the angles between the aromatic rings and the backbone (left: θ_2, C5-C4-C1-C2 and right: θ, C11-C10-C3=C2, y-axis).

S7.3.3 Variation of conformation with Hammett Parameter

Hammett parameters, where available, were taken from the work of Hansch et al.[198]

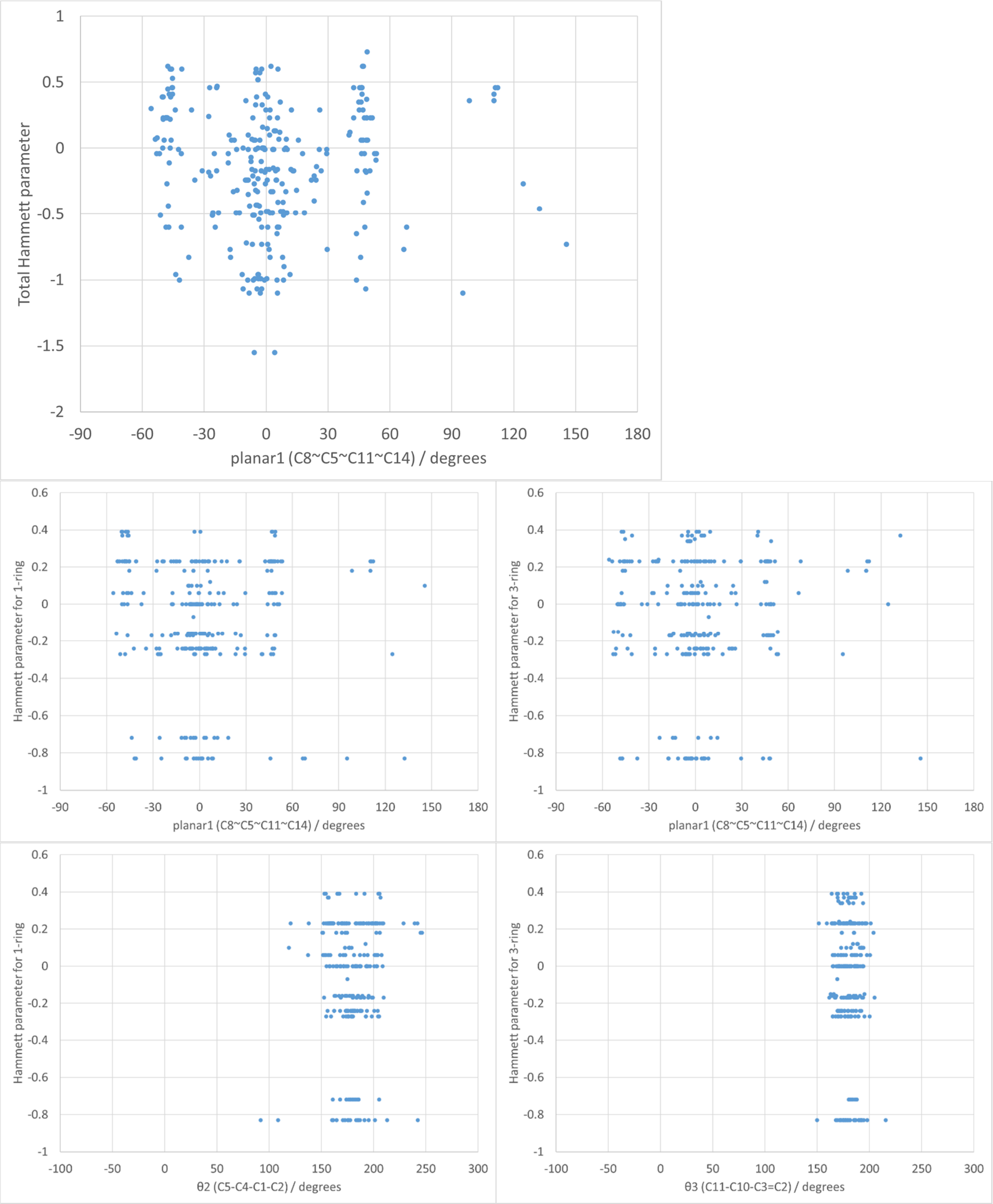

Figure S8. Variation of conformation of chalcone molecules of the Experimental Set with Hammett parameter.

S7.3.4 Variation of packing efficiency with conformation

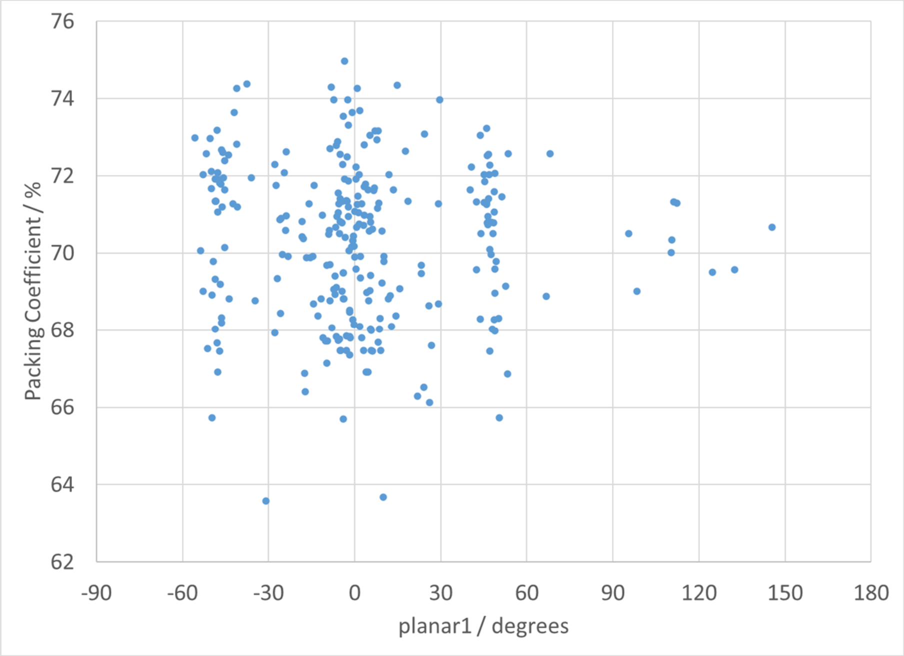

Figure S9. Plot of packing efficiency versus planar1 angle, for all the crystal structures of the experimental set. For structures with Z'>1, points for all conformations are plotted.

Figure S9 shows that the significant number of non-planar molecules (planar1 ~ ±50°) have a very similar distribution of packing efficiencies to the planar molecules (planar1 ~ 0°), which is similar to that generally observed for organic molecules. (Spheres have a packing coefficient of 74%.)

S7.3.5 The relationship between molecules in van der Waals contact and inversion and translation related molecules within the coordination ellipsoid.

The types of dimer relationships of molecules in van der Waals contact within the first coordination ellipsoid of a crystal structure depends on the space group, the precise orientation of the molecules with respect to the unit cell and symmetry elements, and Z'. The analysis of the coordination ellipsoid in terms of S1, S2 and S3 is designed to look for molecules that are parallel (S1, the distance between the two molecular planes is only defined if the molecules are related by inversion or translation), to examine the incidence of π stacking. The XPac/Crystal Packing Similarity analysis shows other types of packing motifs. The difference between the view of crystal packing in terms of entire molecules, rather than the van der Waals contact atoms, is important for larger, non-spherical molecules and in considering the interaction energies between molecules, rather than assigning a contribution to the atom-atom contacts.

The role of the inversion and translation related molecules in the overall crystal packing is illustrated for the most common space group (m/s Figure 3) and Z'=1, using the lowest energy P21/c structure from the CSP search, A225. Using Mercury, all molecules with atoms within the van der Waals radius plus 0.3 Å were selected and analyzed in Table S10 and Figure S10, with the molecules colored by symmetry relationship (white = translation, orange = inversion, green = rotoinversion, and pink = glide) to the central molecule (ball and stick). This shows that only half the molecules with the van der Waals contacts to the central molecule are included in the analysis of just inversion and translation.

Table S10. List of contacts around a central molecule in CSP structure A225, a typical P21/c crystal structure. The rows are shaded according to the symmetry operation relating the molecules, grey=translation, orange=inversion, green=rotoinversion, pink=glide. Only dimers with D1<11.15 Å are listed.

I	D1	S1	Plane1-Cent1B	Plane1B-Cent1	Plane Angle	A1	A1a	τ 1	τ 1a	A2	τ 2	S1†	S2	S3	Included in analysis
1	5.07	3.55	3.55	3.55	0.00	44.10	47.33	0.00	0.00	84.39	0.00	3.55	3.64	0.50	Translation
2	6.32	1.27	1.27	1.27	0.00	69.13	96.30	180.00	-180.00	159.85	-180.00	1.27	2.25	-5.94	Inversion
3	6.43	2.41	2.41	2.41	0.00	73.62	45.37	-180.00	180.00	30.95	180.00	2.41	1.81	5.51	Inversion
4	7.55	5.96	5.96	5.96	0.00	104.01	81.78	180.00	180.00	48.37	180.00	5.96	-1.83	5.02	Outside coordination ellipsoid
5	7.77	2.28	2.28	2.28	0.00	40.70	69.34	180.00	-180.00	134.41	180.00	2.28	5.89	-5.44	Inversion
6	8.42	4.82	4.82	4.82	0.00	99.49	119.36	180.00	-180.00	139.78	180.00	4.82	-1.39	-6.43	Outside coordination ellipsoid
7	8.78	1.14	1.14	1.14	0.00	51.58	25.02	-180.00	180.00	46.79	180.00	1.14	5.45	6.01	Inversion
8	9.31	0.00	7.08	2.40	86.89	137.97	113.13	62.67	55.98	48.55	-4.27	4.74	-6.91	6.16	Outside coordination ellipsoid
9	9.71	0.00	3.53	5.95	86.89	109.69	91.29	65.45	59.05	46.71	5.01	4.74	-3.27	6.66	Outside coordination ellipsoid
10	10.13	0.00	8.35	5.22	86.89	145.97	140.64	-175.43	168.82	85.76	-177.50	6.79	-8.40	0.75	Outside coordination ellipsoid
11	10.14	7.10	7.10	7.10	0.00	44.10	47.33	0.00	0.00	84.39	0.00	7.10	7.28	0.99	Outside coordination ellipsoid
12	10.25	0.00	4.80	8.77	86.89	117.65	115.41	173.60	-166.85	83.03	177.04	6.79	-4.76	1.24	Outside coordination ellipsoid

†Where S1 could not be measured as the interplanar separation (in column 3), the average of the perpendicular distances between the geometric centroid of one molecule and average plane of the other molecule was used.

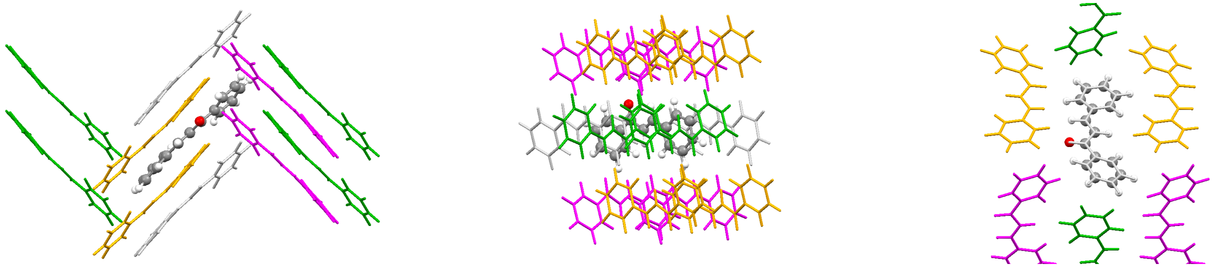

Figure S10. Crystal structure A225 from the CSP search, showing the central molecule colored by element with ball-and-stick depiction and all those with short contacts below the sum of the van der Waals radii + 0.3 Å. (left) viewed along a, (middle) viewed along b, (right) viewed along c. Molecules are colored by symmetry operation, white=translation, orange=inversion, green=rotoinversion, pink=glide.

For this crystal structure, it is clear that all inversion and translation interactions that should be analyzed are within the coordination ellipsoid. The contacts with molecules with rotoinversion or glide symmetry with respect to the central molecule are not included, although they give a lot of information about the structure. These contacts are important in the complete packing description for the crystal structures, but are only analyzed within the XPac and Crystal Packing Similarity analyses. There are sufficient inversion and translation dimers being analyzed to give an overview of trends in the crystal packing across the sets of crystal structures.

Only two crystal structures in the Experimental Set have no inversion or translation dimers within this definition; high symmetry structure A-21o (in $P4_12_12$) and Z'=2 structure Mo3p. In the CSP Set there are 3.3% (156 structures) which do not have any inversion or translation dimers, but these are all very unstable, low-density structures.

Note that inversion interactions are different on different sides of the molecule, whereas translation interactions are the same, and so there are more inversion related dimers than translation related dimers. However, 25% of the structures in the Experimental Set do not have an inversion centre in the list of close contacts, although only 14% are in non-centrosymmetric space groups (Figure 3a in manuscript). A higher proportion (35%) of structures in the CSP set do not have inversion dimers.

There were 747 inversion and 408 translation dimer contacts in the 232 structures of the Experimental Set and 8,540 inversion and 6,526 translation dimer contacts in the CSP Set. The larger number of motifs in the CSP set helps establish the correlation (or not) with the lattice energy, as if there had been a particular dimer contact that gave a dominant energetic contribution, this would show in Figure 8 of the manuscript.

S7.3.6 Close contacts in the Experimental and CSP Sets

To test comparability, within the lowest energy 232 structures of the CSP Set (to give the same number of crystal structures as in the Experimental Set) there were 493 inversion and 292 translation related close contacts. The greater number of close contacts in the Experimental Set is mainly because of the difference in definition of the first coordination ellipsoid for the two sets of data, made to allow for the size of the substituents. If you use the Experimental set definition of the first coordination ellipsoid, then there are 701 inversion and 373 translation close contacts in the comparably sized CSP set, which is very similar to the 747 inversion and 408 translation motifs in the Experimental Set. We note that a significant difference between the two data sets is that isostructural crystals in the experimental set are retained, whereas they are removed from the CSP set as duplicates.

The inversion and translation close contacts were plotted as 3-D scatterplots, for the Experimental and CSP Sets of structures separately. These are given in Figure 7 of the manuscript, colored by S1. The same 3-D scatterplots of the close contacts of the CSP Set are given colored by the total lattice energy, as Figure 8 in the main paper. In addition, these 3-D scatterplots are plotted colored by various molecular geometric parameters (planar1, and the torsion angles θ) in Figure S11 for the Experimental Set and Figure S12 for the CSP Set, and by substituent, position and substituent Hammett parameter on Ring 1 in Figure S13 and Ring 3 in Figure S14.

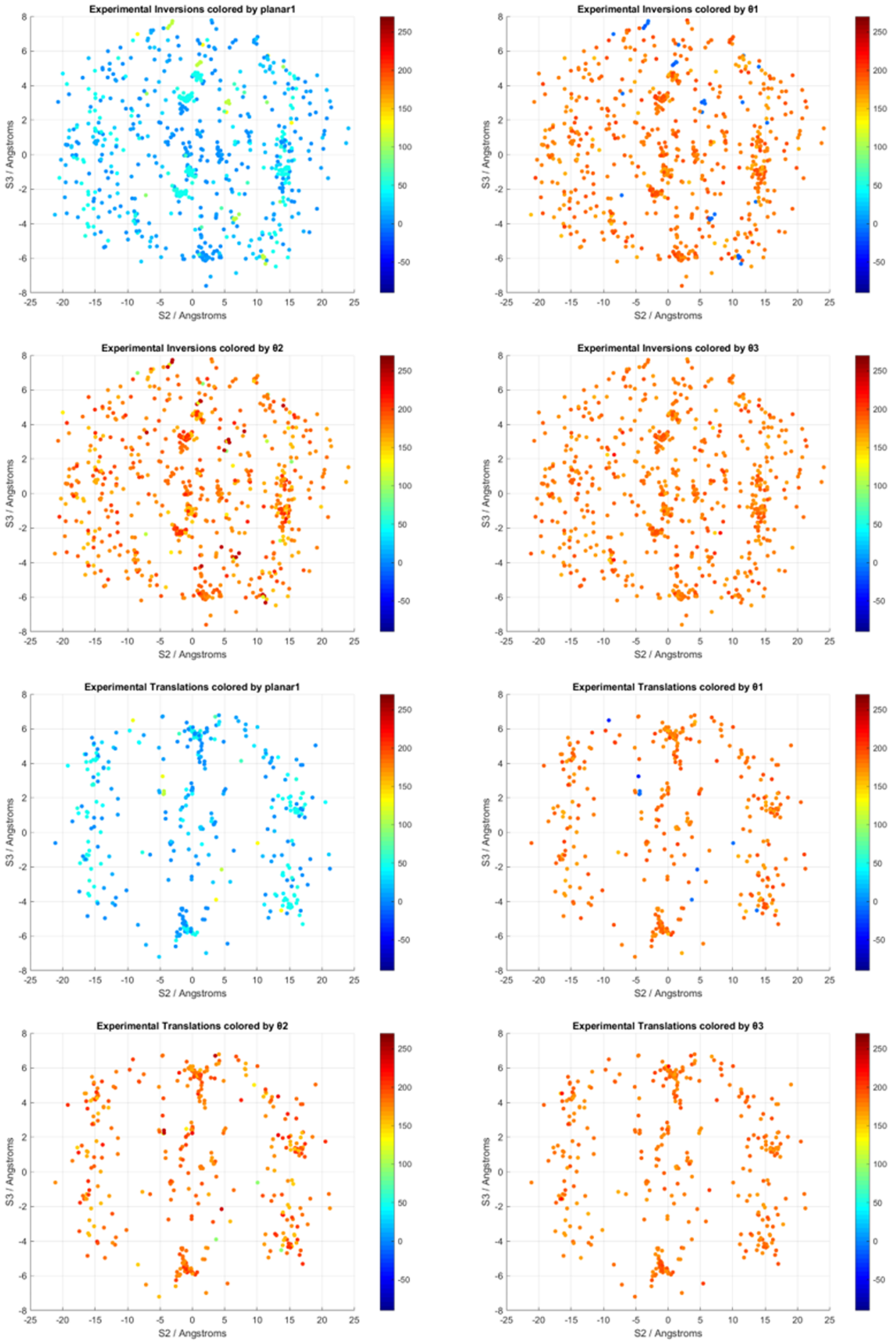

Figure S11. Scatterplots of S2 (end-to-end displacement) vs S3 (side-to-side displacement), for the Experimental Inversion and Translation contacts, colored by the main geometric parameters (planar1:C8~C5~C11~C14, θ_1:C4-C1-C2=C3, θ_2:C5-C4-C1-C2, and θ_3:C2=C3-C10-C11).

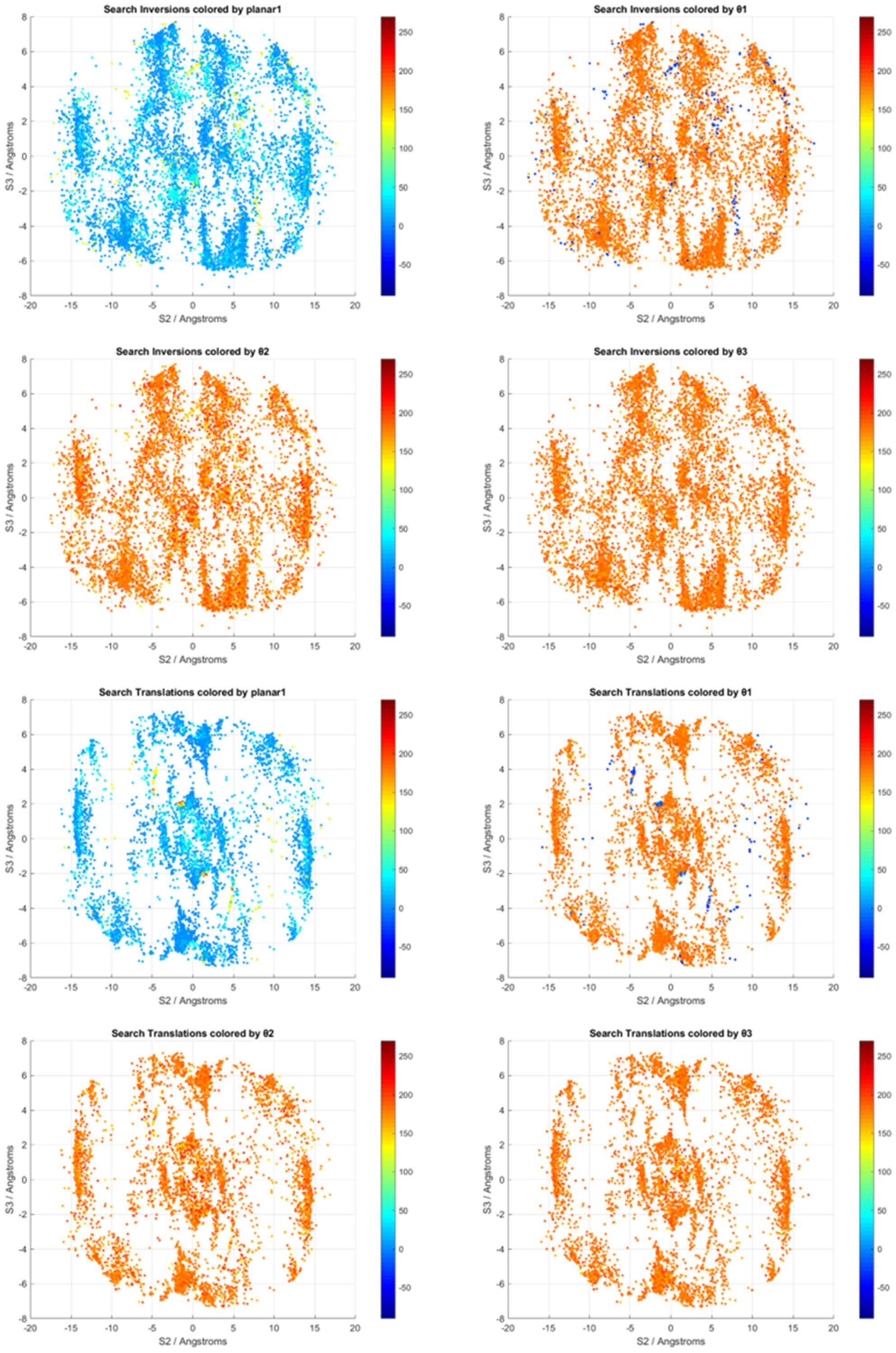

Figure S12. Scatterplots of S2 (end-to-end displacement) vs S3 (side-to-side displacement), for the Search Inversion and Translation contacts, colored by the main geometric parameters (planar1:C8~C5~C11~C14, θ_1:C4-C1-C2=C3, θ_2:C5-C4-C1-C2, and θ_3:C2=C3-C10-C11).

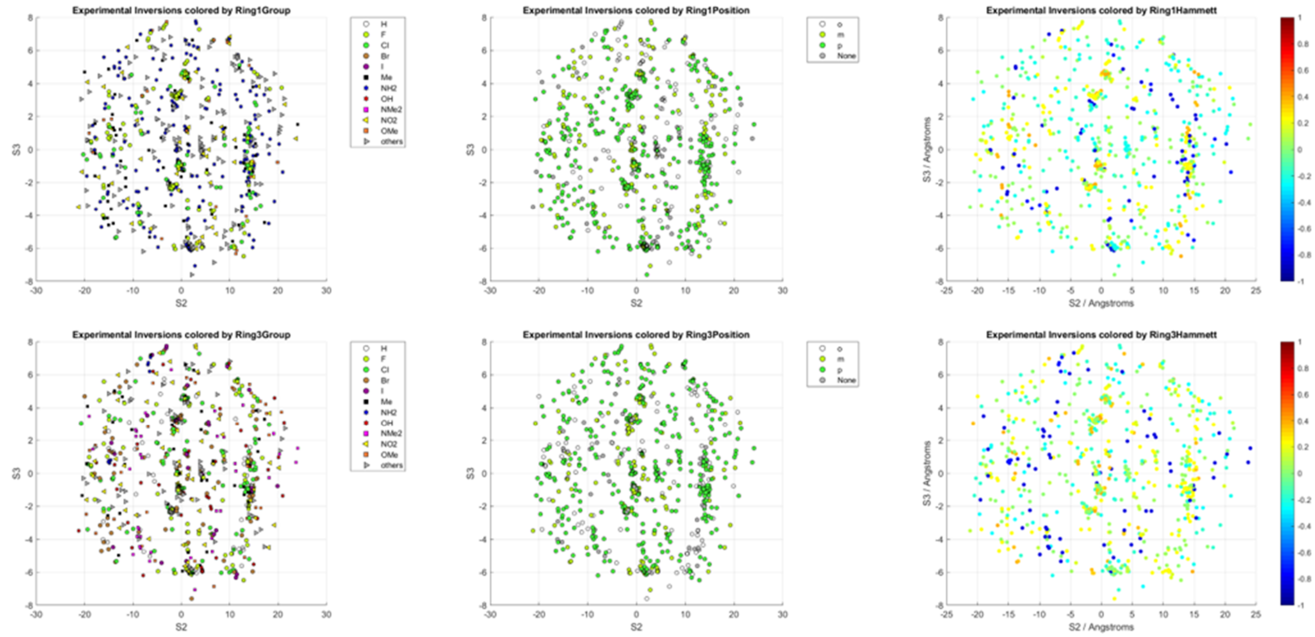

Figure S13. Scatterplots of S2 (end-to-end displacement) vs S3 (side-to-side displacement), for the Experimental Inversion contacts, colored by the substituents and positions of the substituents on the two rings, and the Hammett parameter of the substituents.

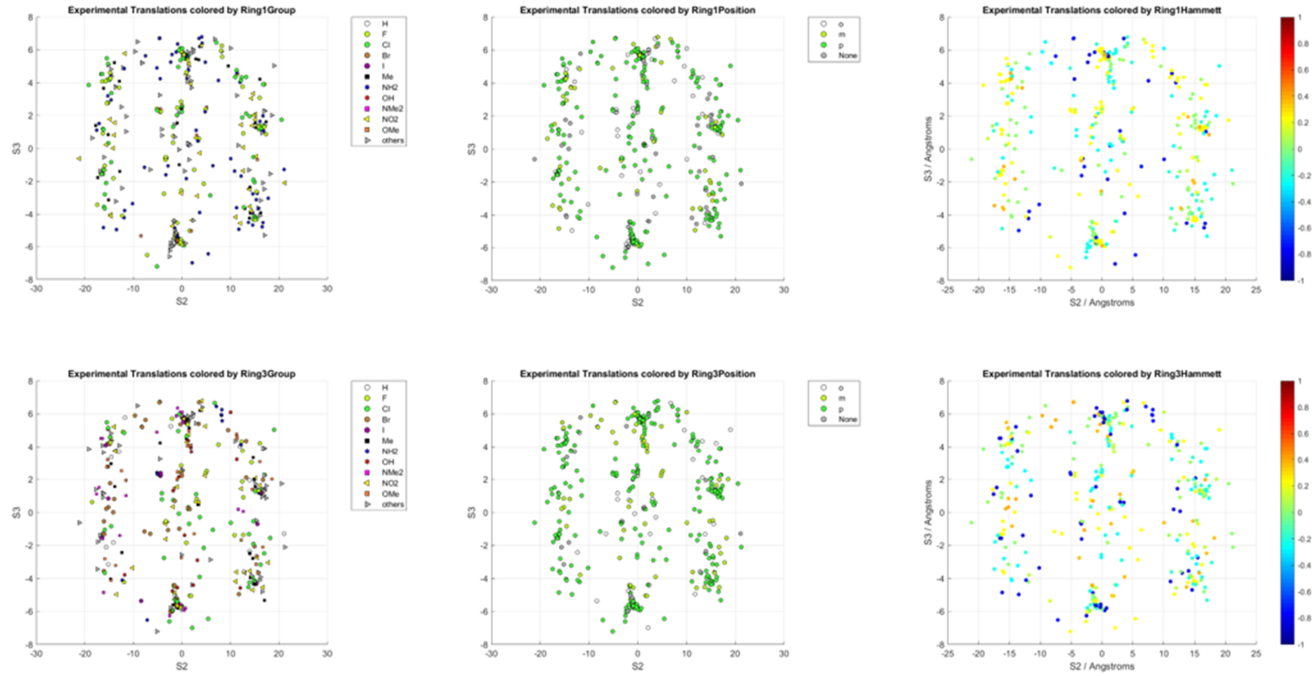

Figure S14. Scatterplots of S2 (end-to-end displacement) vs S3 (side-to-side displacement), for the Experimental Translation contacts, colored by the substituents and positions of the substituents on the two rings, and the Hammett parameter of the substituents.

S7.3.7 Highly populated areas of the S1/S2/S3 charts

The 3-D space defining the close contacts within the Experimental Set and CSP Set was divided into boxes, spaced at 0.25 Å intervals in S1 and 1 Å intervals in S2 and S3. All the close contacts within each volume were counted, and the results are given in Figure S15, Figure S16, Figure S17, and Figure S18. Boxes containing 5+ interactions for the Experimental Set or 30+ for the CSP Set are listed in and grouped together in Table S12. A group of boxes is defined as a region where boxes with high populations are adjacent, including diagonally. These may be extended to be the same length the whole way across. In the case of translation interactions, symmetry means that opposite regions are equivalent and these are combined in Table S11 and Table S12.

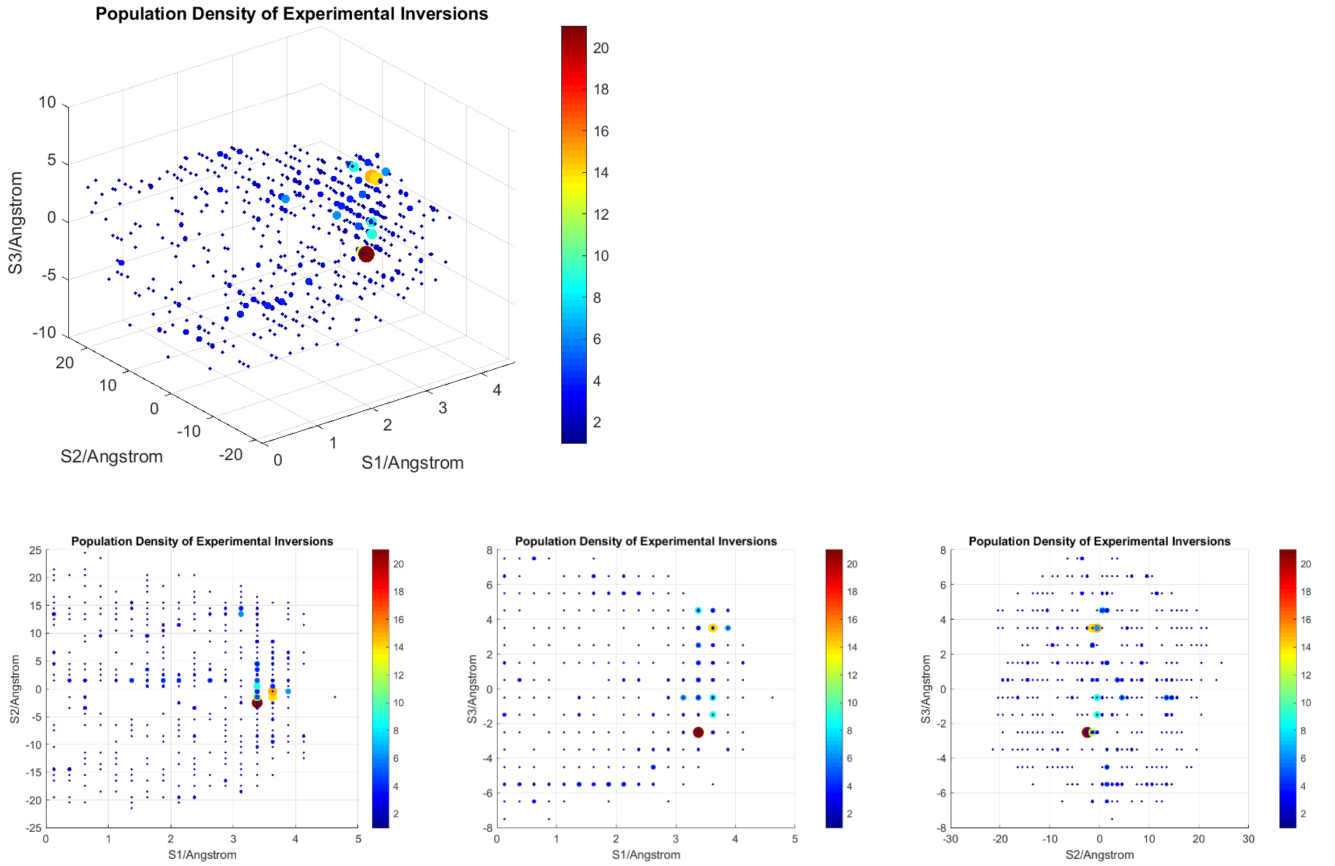

Figure S15. Population Density of inversion contacts in the Experimental Set. S1 is divided into 0.25 Å intervals, and S2 and S3 are divided into 1 Å intervals. The color and size of the points are determined by the number of inversion contacts (in the first coordination ellipsoid) in that box.

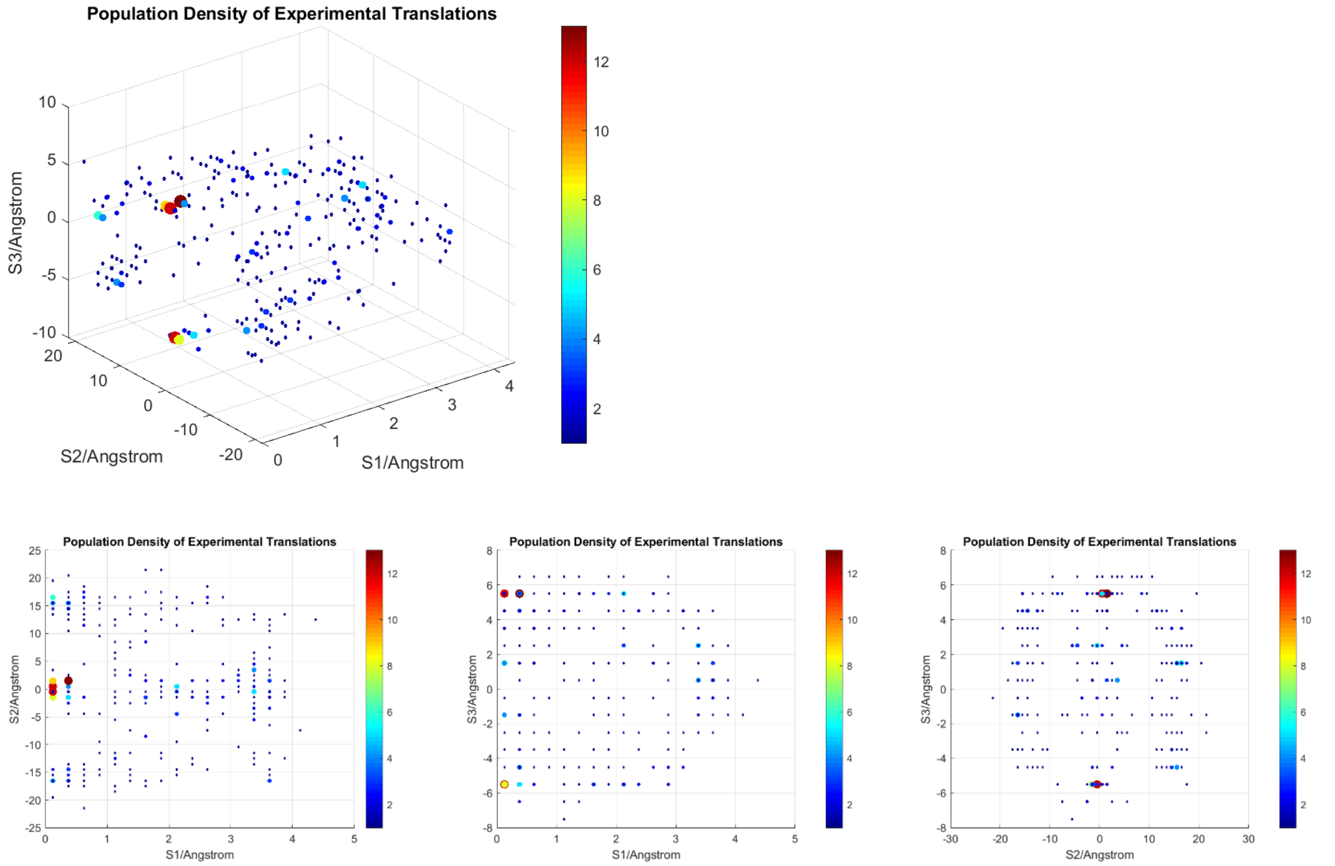

Figure S16. Population Density of translation contacts in the Experimental Set. S1 is divided into 0.25 Å intervals, and S2 and S3 are divided into 1 Å intervals. The color and size of the points are determined by the number of translations contacts (in the first coordination ellipsoid) in that box.

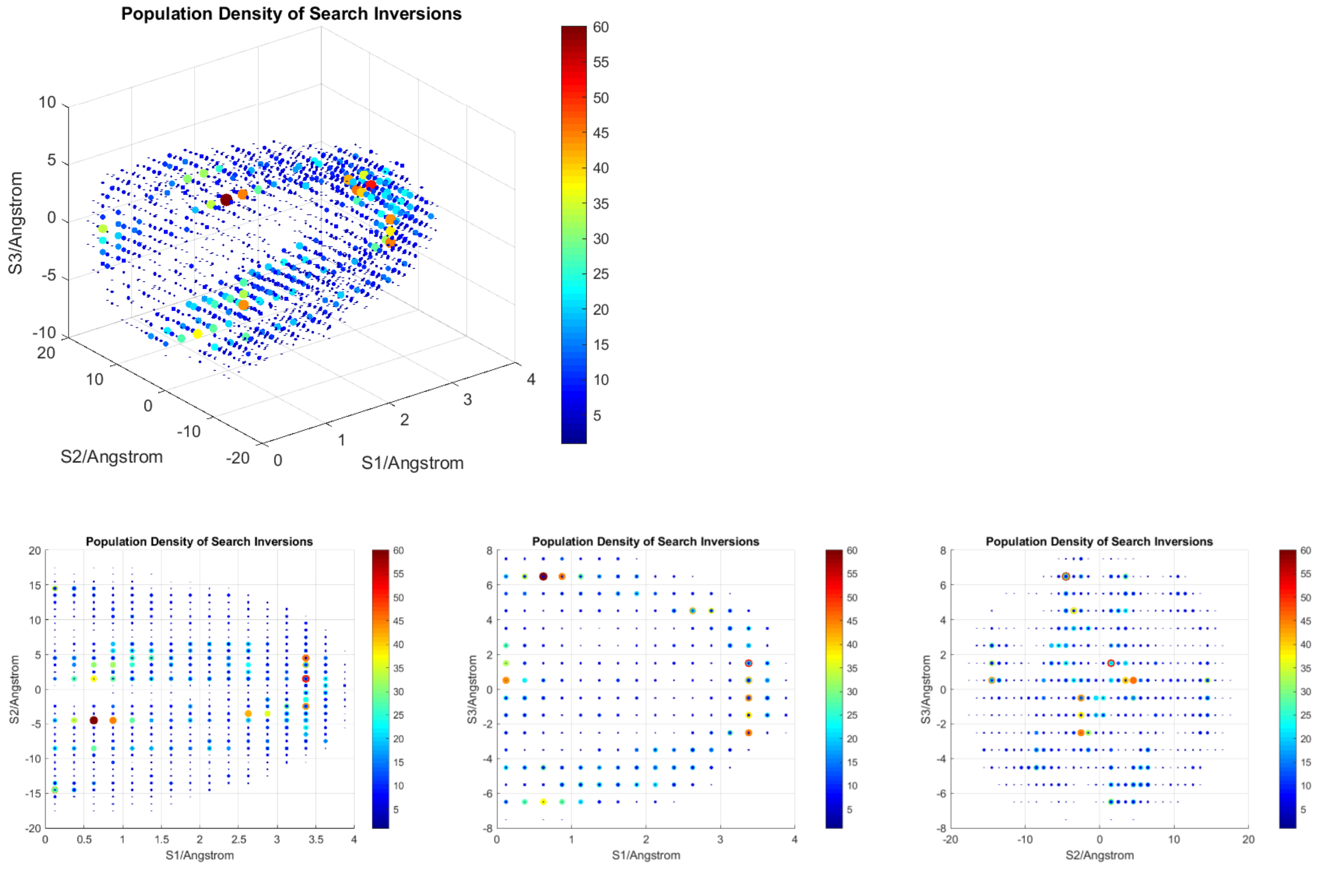

Figure S17. Population Density of inversion contacts in the CSP Set. S1 is divided into 0.25 Å intervals, and S2 and S3 are divided into 1 Å intervals. The color and size of the points are determined by the number of inversion contacts (in the first coordination ellipsoid) in that box.

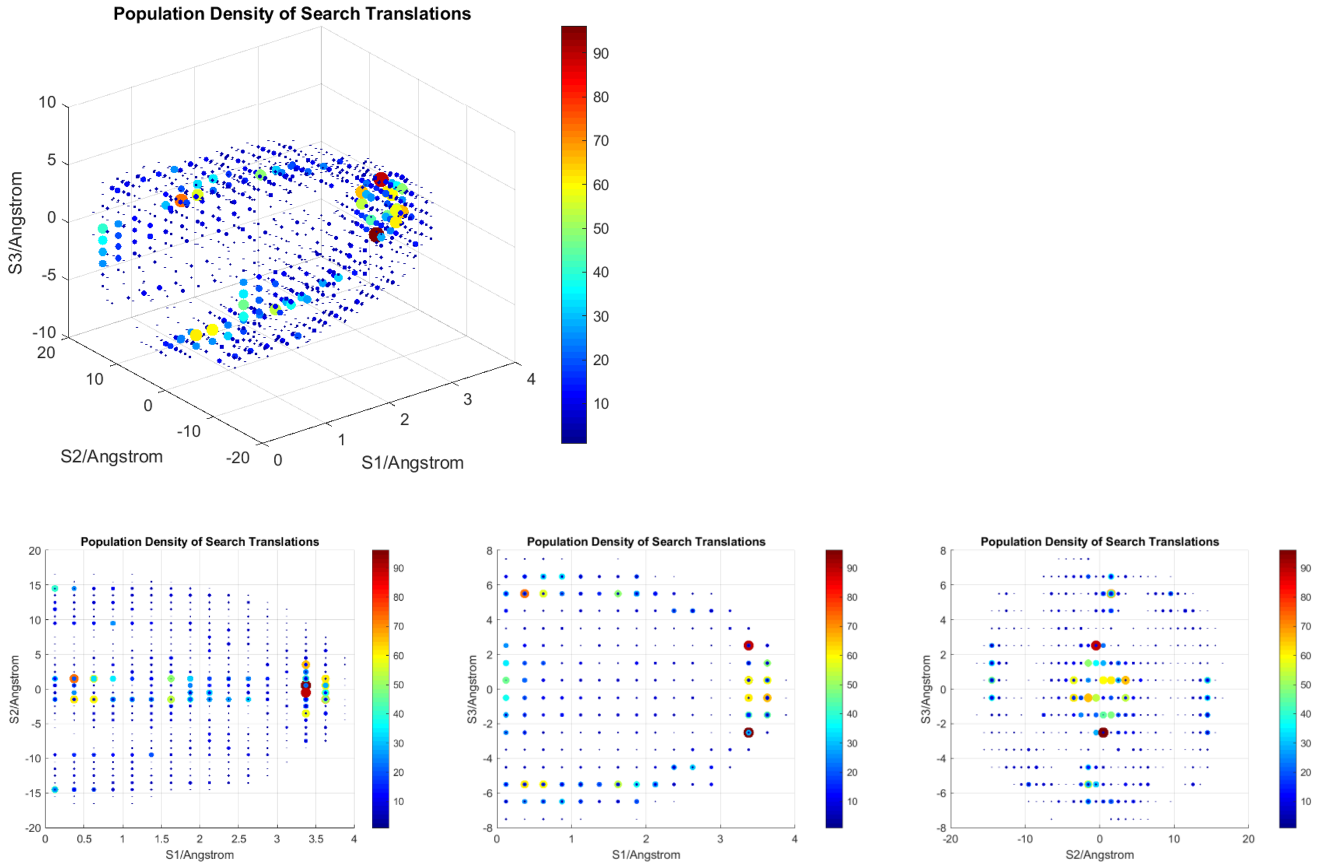

Figure S18. Population Density of translation contacts in the CSP Set. S1 is divided into 0.25 Å intervals, and S2 and S3 are divided into 1 Å intervals. The color and size of the points are determined by the number of translations contacts (in the first coordination ellipsoid) in that box.

Table S11. All regions of Figures S12-S15 that contain 5+ contacts for the structures in the Experimental Set or 30+ contacts for the structures of the CSP Set.

S1min	S1max	S2min	S2max	S3min	S3max	Count	Motif
Inversion contacts in Experimental Set							
3	3.25	13	14	-1	0	6	I9
3.25	3.5	-3	-2	-3	-2	21	I1
3.25	3.5	-2	-1	-3	-2	12	I1
3.25	3.5	-2	-1	2	3	5	I2
3.25	3.5	-1	0	-1	0	5	I1
3.25	3.5	0	1	4	5	9	I2
3.25	3.5	1	2	4	5	5	I2
3.25	3.5	4	5	-1	0	6	I3
3.5	3.75	-2	-1	3	4	14	I2
3.5	3.75	-1	0	-2	-1	9	I1
3.5	3.75	-1	0	-1	0	9	I1
3.5	3.75	-1	0	3	4	15	I2
3.75	4	-1	0	3	4	6	I2
Inversion contacts in CSP Set							
0	0.25	-15	-14	0	1	44	I6
0	0.25	-15	-14	1	2	33	I6
0	0.25	14	15	0	1	34	I7
0.25	0.5	-5	-4	6	7	34	I4
0.5	0.75	-5	-4	6	7	60	I4
0.5	0.75	1	2	-7	-6	37	I5
0.5	0.75	3	4	6	7	31	I8
0.75	1	-5	-4	6	7	45	I4
0.75	1	3	4	6	7	32	I8
2.5	2.75	-4	-3	4	5	42	I10
2.75	3	-4	-3	4	5	36	I10
3.25	3.5	-3	-2	-3	-2	45	I1
3.25	3.5	-3	-2	-2	-1	38	I1
3.25	3.5	-3	-2	-1	0	44	I1
3.25	3.5	-2	-1	-3	-2	32	I1
3.25	3.5	1	2	1	2	51	I2
3.25	3.5	3	4	0	1	40	I3
3.25	3.5	4	5	0	1	46	I3

S1min	S1max	S2min	S2max	S3min	S3max	Count	Motif
Translation contacts in Experimental Set							
0	0.25	-2	-1	-6	-5	8	T1
0	0.25	-1	0	-6	-5	12	T1
0	0.25	0	1	5	6	12	T1
0	0.25	1	2	5	6	9	T1
0	0.25	16	17	1	2	6	T5
0.25	0.5	-2	-1	-6	-5	5	T1
0.25	0.5	1	2	5	6	13	T1
2	2.25	0	1	5	6	5	T2
3.25	3.5	-1	0	2	3	5	T3
Translation contacts in CSP Set							
0	0.25	-15	-14	-1	0	38	T5
0	0.25	-15	-14	0	1	47	T5
0	0.25	-15	-14	1	2	35	T5
0	0.25	14	15	-1	0	36	T5
0	0.25	14	15	0	1	41	T5
0.25	0.5	-2	-1	-6	-5	61	T1
0.25	0.5	1	2	5	6	73	T1
0.5	0.75	-2	-1	-6	-5	59	T1
0.5	0.75	1	2	5	6	57	T1
0.5	0.75	1	2	6	7	33	T1
0.75	1	-2	-1	-7	-6	31	T1
0.75	1	1	2	6	7	34	T1
1.5	1.75	-2	-1	-6	-5	53	T2
1.5	1.75	1	2	5	6	49	T2
1.75	2	-2	-1	-6	-5	38	T2
1.75	2	1	2	5	6	32	T2
2	2.25	-1	0	-6	-5	32	T2
2.5	2.75	-2	-1	-5	-4	31	T2
3.25	3.5	-4	-3	-1	0	61	T4
3.25	3.5	-4	-3	0	1	58	T4
3.25	3.5	-1	0	2	3	89	T3
3.25	3.5	0	1	-3	-2	96	T3
3.25	3.5	1	2	-2	-1	43	T3
3.25	3.5	3	4	-1	0	54	T4
3.25	3.5	3	4	0	1	65	T4
3.5	3.75	-2	-1	-1	0	65	T3
3.5	3.75	-2	-1	1	2	49	T3
3.5	3.75	-1	0	-1	0	54	T3
3.5	3.75	-1	0	1	2	36	T3
3.5	3.75	0	1	-2	-1	45	T3
3.5	3.75	0	1	0	1	60	T3
3.5	3.75	1	2	-2	-1	48	T3
3.5	3.75	1	2	0	1	61	T3

Table S12. Common inversion and translation motifs in the coordination ellipsoid for both the Experimental and CSP Sets. The motifs correspond to the given rectangular volumes which are listed in Table S11 as being highly populated. The example shown is the contact closest to the centre of the area of interest from the structures of the CSP Set. Where different definitions are used for the contacts within the Experimental Set and the CSP Set, they are shown in red and blue respectively.

Motif	Definition	Example	Example	# in Experimental Set	# in CSP Set
I1, face-to-face inversions	3.25<S1<3.75<br>-3<S2<0<br>-3<S3<0<br>3.25<S1<3.5<br>-3<S2<-1<br>-3<S3<0	A13629<br>S1=3.42<br>S2=-2.11<br>S3=-1.52	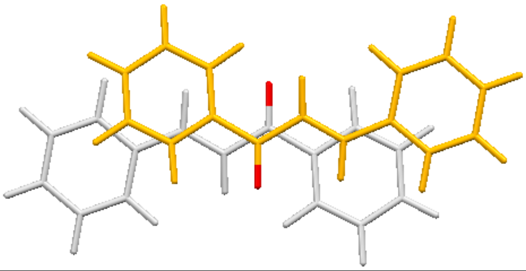	69 (23%)	177 (3.5%)
I2, face-to-face inversions	3.25<S1<4<br>-2<S2<2<br>2<S3<5<br>3.25<S1<3.5<br>1<S2<2<br>1<S3<2	A18179<br>S1=3.31<br>S2=1.44<br>S3=1.51	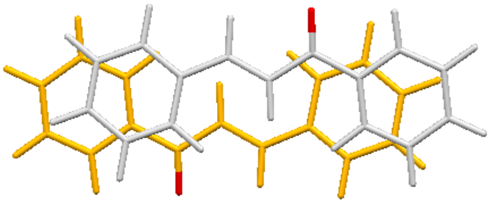	72 (22%)	51 (1.0%)
I3, face-to-face inversions	3.25<S1<3.5<br>4<S2<5<br>-1<S3<0<br>3.25<S1<3.5<br>3<S2<5<br>0<S3<1	A13078<br>S1=3.30<br>S2=4.02<br>S3=0.57	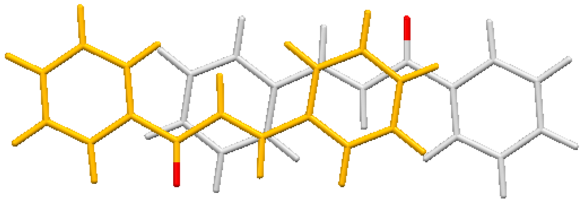	6 (2.2%)	86 (1.7%)
I4, side-to-side inversions	0.25<S1<1<br>-5<S2<-4<br>6<S3<7	A10286<br>S1=0.64<br>S2=-4.48<br>S3=6.64	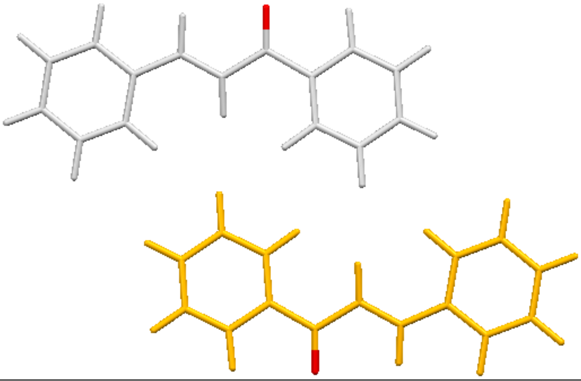	none	139 (2.8%)
I5, side-to-side inversions	0.5<S1<0.75<br>1<S2<2<br>-7<S3<-6	A11777<br>S1=0.53<br>S2=1.44<br>S3=-6.20	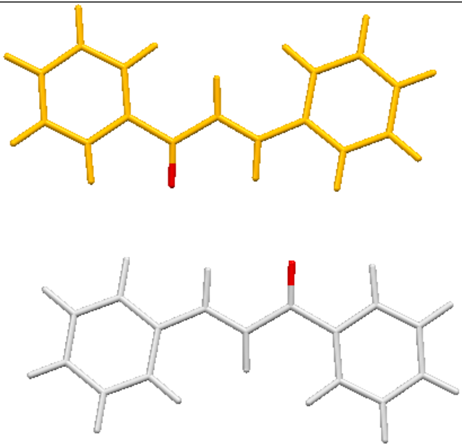	3 (1.3%)	37 (0.7%)

I6, end-to-end inversions	0<S1<0.25<br>-15<S2<-14<br>0<S3<2	A708<br>S1=0.08<br>S2=-14.37<br>S3=1.08	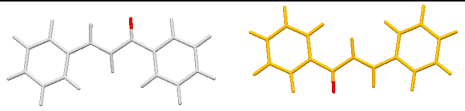	2 (0.9%)	77 (1.5%)
I7, end-to-end inversions	0<S1<0.25<br>14<S2<15<br>0<S3<1	A16098<br>S1=0.16<br>S2=14.70<br>S3=0.40	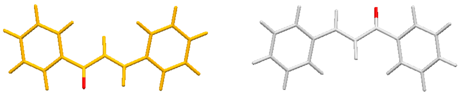		34 (0.7%)
I8, side-to-side and end-to-end	0.5<S1<1<br>3<S2<4<br>6<S3<7	A3708<br>S1=0.71<br>S2=3.53<br>S3=6.44	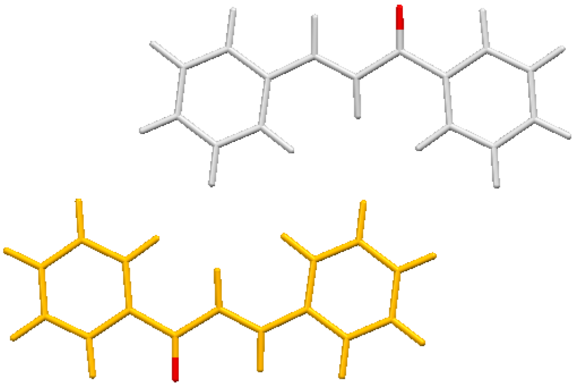	none	63 (1.3%)
I9, face-to-face and end-to-end	3<S1<3.25<br>13<S2<14<br>-1<S3<0	A10586<br>S1=2.87<br>S2=12.03<br>S3=0.56	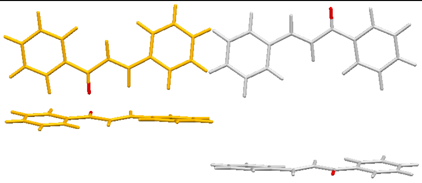	6 (2.6%)	none
I10, offset in all directions	2.5<S1<3<br>-4<S2<-3<br>4<S3<5	A18010<br>S1=2.81<br>S2=-3.50<br>S3=4.42	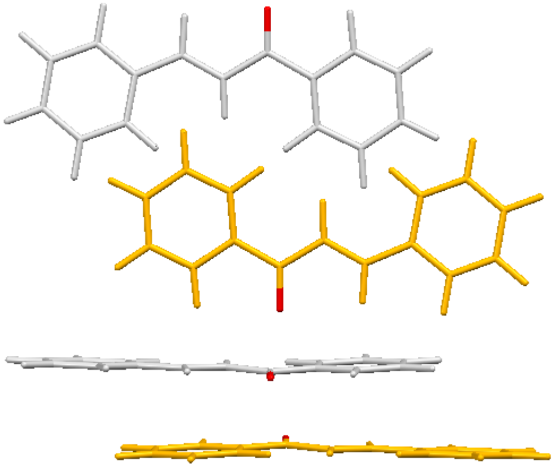	none	78 (1.5%)
T1, side-to-side translations	0<S1<0.5<br>-2<S2<0;<br>0<S2<2<br>-6<S3<-5;<br>5<S3<6<br>0.25<S1<1<br>-2<S2<-1;<br>1<S2<2<br>-7<S3<-5;<br>5<S3<7	A17562<br>S1=0.63<br>S2=1.48<br>S3=5.86	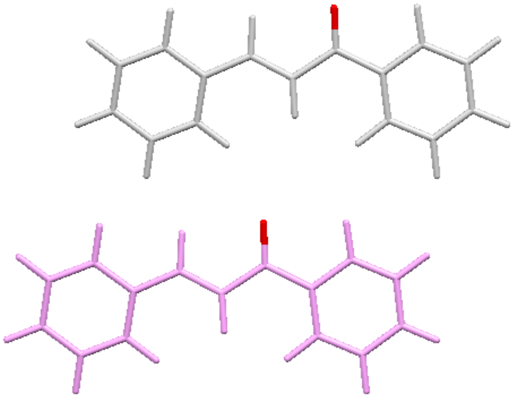	65 (27%)	463 (8.8%)

T2, side-to-side translations	2<S1<2.25<br>0<S2<1<br>5<S3<6<br>1.5<S1<2.75<br>-2<S2<0;<br>1<S2<2<br>-6<S3<-4;<br>5<S3<6	A12360<br>S1=2.10<br>S2=-1.06<br>S3=-5.10	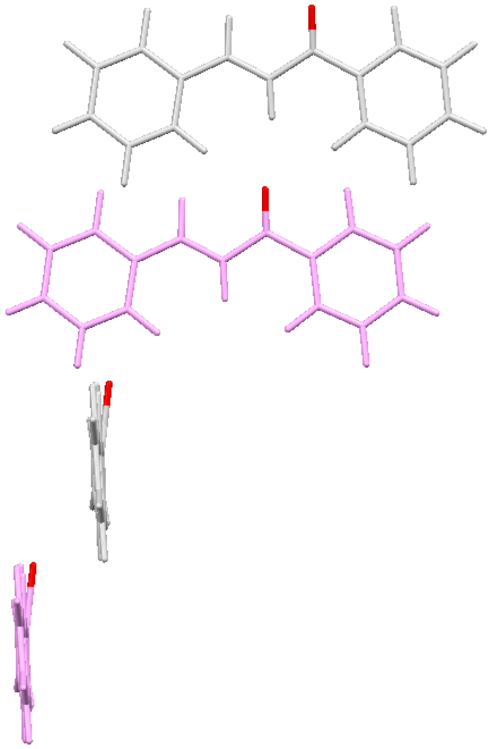	5 (2.2%)	384 (7.2%)
T3, face-to-face translations	3.25<S1<3.5<br>-1<S2<0<br>2<S3<3<br>3.25<S1<3.75<br>-2<S2<2<br>-3<S3<3	A4361<br>S1=3.66<br>S2=0.00<br>S3=0.02	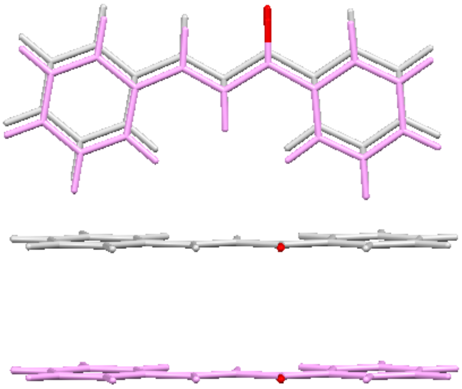	5 (2.2%)	1030 (18%)
T4, face-to-face translations	3.25<S1<3.5<br>-4<S2<-3;<br>3<S2<4<br>-1<S3<1	A11049<br>S1=3.36<br>S2=-3.52<br>S3=-0.05	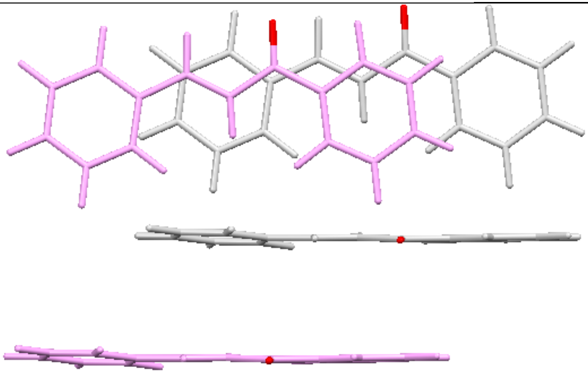	7 (3.0%)	238 (4.4%)
T5, end-to-end translations	0<S1<0.25<br>16<S2<17<br>1<S3<2<br>0<S1<0.25<br>-15<S2<-14;<br>14<S2<15<br>-1<S3<2	A9986<br>S1=0.15<br>S2=-14.48<br>S3=0.48	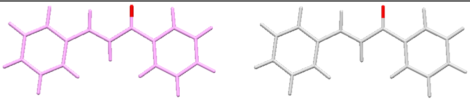	6 (2.6%)	199 (3.9%)

Table S12 shows the highly populated areas of the 3d scatterplots of S1, S2 and S3. There are more distinct regions in the inversion close contacts than in the translation close contacts. This is partly because there can be many more inversion contacts for a molecule, since interactions on opposite sides are different (see Section S7.3.5).

S8. Common 1-D motifs

The most prevalent motif found is a 1-D, translation-related line of molecules. This occurred in three different manners, all closely related. The most common featured non-coplanar rings, and is defined by

$S1<0.7 \wedge -2.6<S2<2.6 \wedge (-6.3<S3<-5 \vee 5<S3<6.3) \rightarrow T1$

since this includes all the dimer contacts that are within the cluster (Figure S19). The less populated T2 motif is defined by

$2<S1<2.5 \wedge 0<S2<1 \wedge 5<S3<6 \rightarrow T2$

The definitions of T1 and T2 are close on the 3-D scatterplots of Figure S16, although Table S11 shows that there are distinct boxes between the two regions without 5 contacts. However, close inspection of Figure 7b in the main paper shows that this area of the scatterplot is more diffuse, and so a better definition is

$((-3<S2<1.5 \wedge -7<S3<-3.5) \vee (-1.5<S2<3 \wedge 3.5<S3<7)) \wedge |S1|<|S3| \rightarrow TrSS$

which encompasses all the 1-D side-to-side contacts for Region A conformations in the translation plots (the final |S1|<|S3| is to ensure that the contact is side-to-side rather than face-to-face, since this approaches a spectrum). Within this large set, the contacts can be sub divided as above, or divided by conformation.

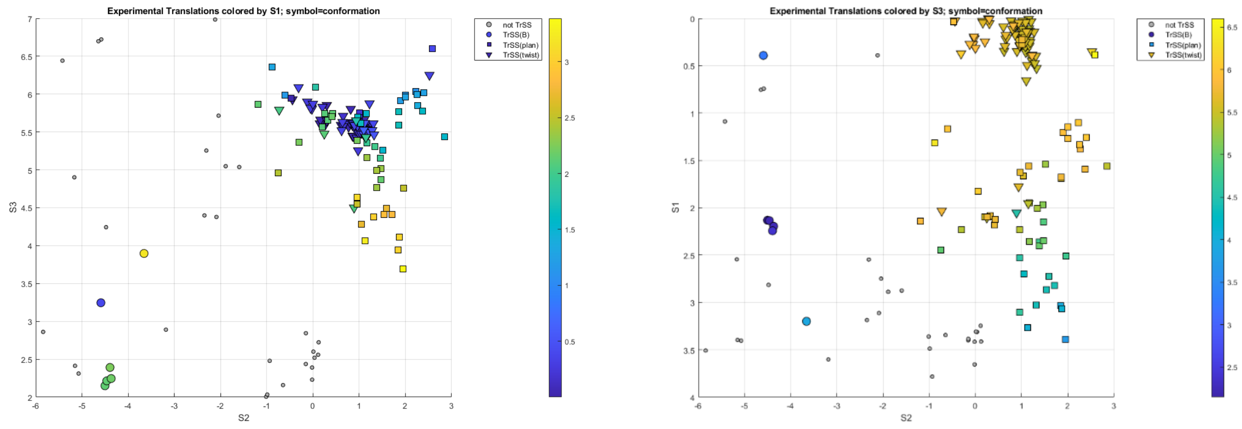

Figure S19. Enlargement of the section of Figure 7(b) of the main paper which shows the TrSS motif, with points with negative S3 values moved to the upper section. Part (a) view along S1 colored by S1 and part (b) view along S3 colored by S3. The colored triangles and squares show -1.5<S2<3, 3.5<S3<7, S1<S3, with triangles for |planar1|>30 and squares |planar1|<30. The colored circles show θ_1<90° ($TrSS_B$). All other points on the plot are outside these three definitions.

Figure S19 shows that, *in general*, the T1 contacts contain the molecule with planar1 ~ ±50°, while the contacts within the wider 1-D definition that are not within the T1 definition usually contain molecules with planar1 ~ 0°. For Z'>1 structures, the molecular parameters are those of the first molecule, and the contact may involve the second or later independent molecule, which can have a different conformation. These account for the squares in the region of Figure S19 which contains the $TrSS_{twist}$ motif and the triangles in the region of Figure S19 that contains the $TrSS_{plan}$ motif. Figure S20 shows the histograms of planar1 angle within the structures with T1 contacts (part (a)) and TrSS contacts not within T1 (part (b)). Almost all the contacts which contain discrepant planar1 values are for higher Z' structures (Ho2p is the exception, and the large angle of one of the rings is caused by the ortho nitro substituent). Some structures contain multiple 1-D contacts, such as A-6p-II, Hp4p, Dp7p, Hp6p, Bp21p, Cp7p-b and Jp7p.

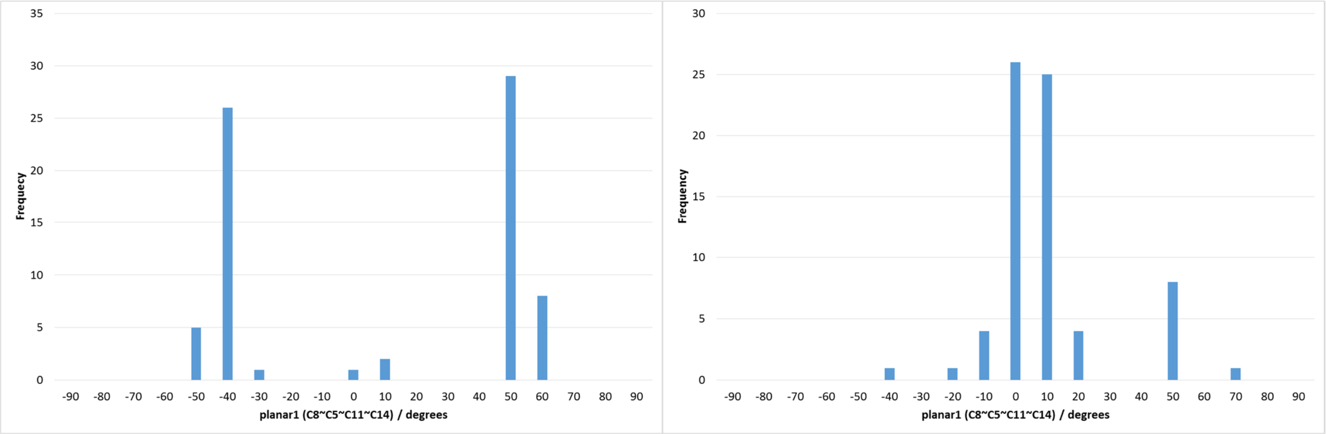

Figure S20. Histogram of planar1 values for crystal structures in the experimental set (a) that contain the T1 dimer motif, and (b) that contain the TrSS motif but neither the T1 dimer motif nor the Region B conformation.

A set of contacts closely related to TrSS, where the molecule contains the Region B conformation ($\theta_1 < 90°$), contains 6 structures (of the 8 structures of exclusively Region B conformations) which share this common side-to-side contact ($TrSS_B$ on Figure S19). This is defined by

$\theta_1 < 90 \wedge ((3<S2<7 \wedge -5<S3<-2) \vee (-7<S2<-3 \wedge 2<S3<5)) \wedge |S1|<|S3| \rightarrow TrSS_B$.

This can be compared with the definition

$((-3<S2<1.5 \wedge -7<S3<-3.5) \vee (-1.5<S2<3 \wedge 3.5<S3<7)) \wedge |S1|<|S3| \rightarrow TrSS$

which shows that the very different shape of the Region B molecules means that it falls outside the definition of TrSS because of the different shape, despite also being a close-packed translation motif.

S8.1.1 Alternative views of the comparison between $TrSS_{twist}$ and $TrSS_{plan}$ motifs

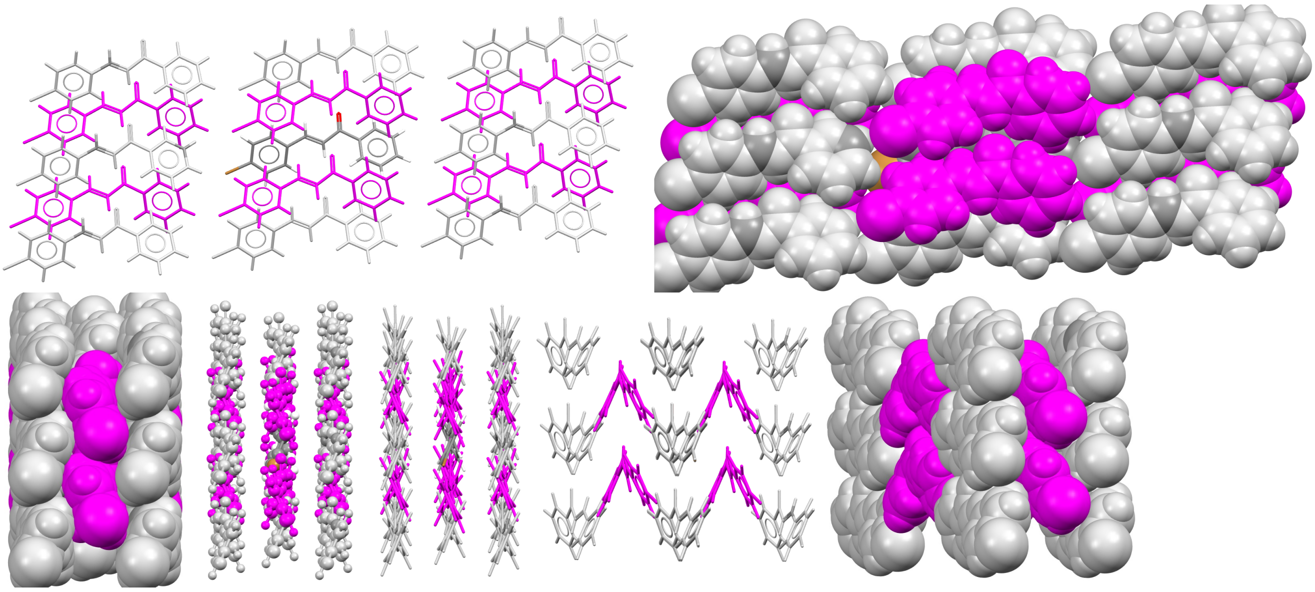

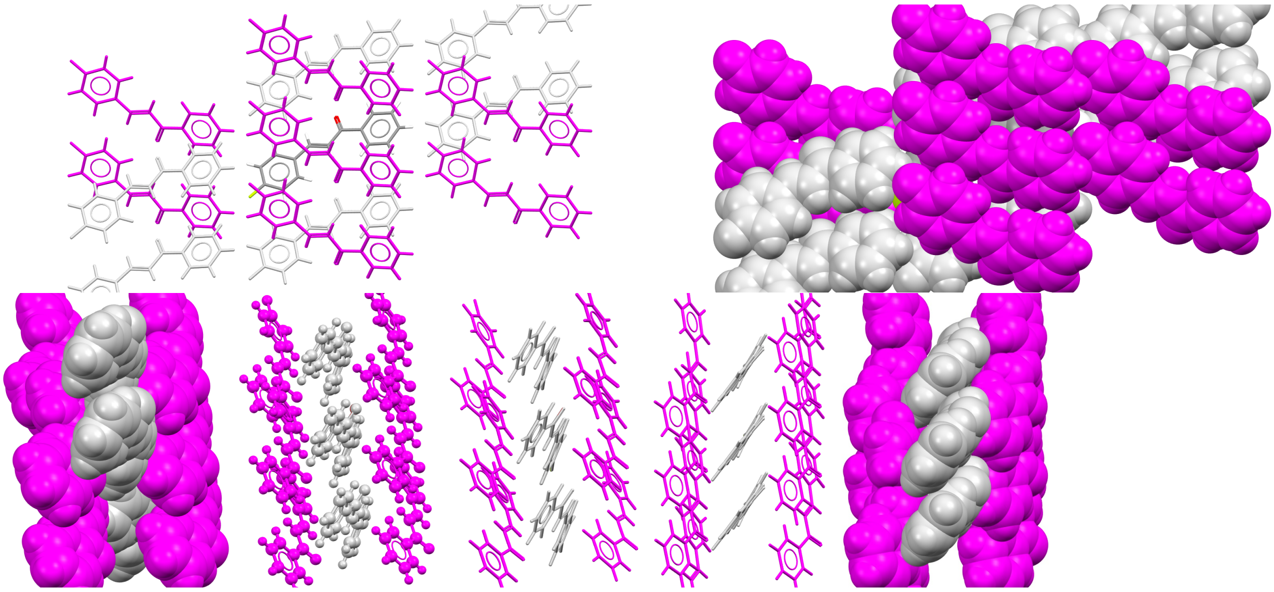

Figure S21. Some pictures of TARCIY (A-4p) which has the $TrSS_{twist}$ motif (defined as having molecules with planar1~±50°) shown in grey for the translation related molecules and in magenta for the glide related molecules (relative to the molecule in atomic colors) and BUDYOP (A-2p-I) showing the $TrSS_{plan}$ motif (planar1=0). These crystal structures are in the same space group, so have similar packings of the 1-D motifs.

S9. Mercury's Crystal Packing Similarity and XPac analysis

The crystal structures in the Experimental Set were inspected for their similarities using the pairwise comparisons of Mercury's Crystal Packing Similarity tool and XPac. For Mercury's Crystal Packing Similarity tool, the number of molecules in common was recorded, and for XPac, the type of the common feature was recorded (see Table S13).

Table S13. Description and codes used for XPac analysis.

Description	Code	Description	Code
No match	0	1-D match (chain / ribbon)	5
0-D match (eg centrosymmetric dimer)	1	Doubled 1-D match	6
2 distinct 0-D matches	2	2-D match (sheet)	7
Approximately 1-D match, often when one structure is Z'=2	3	Doubled 2-D match	8
Doubled approximately 1-D, as above, but doubled up	4	3-D match (isostructural)	9

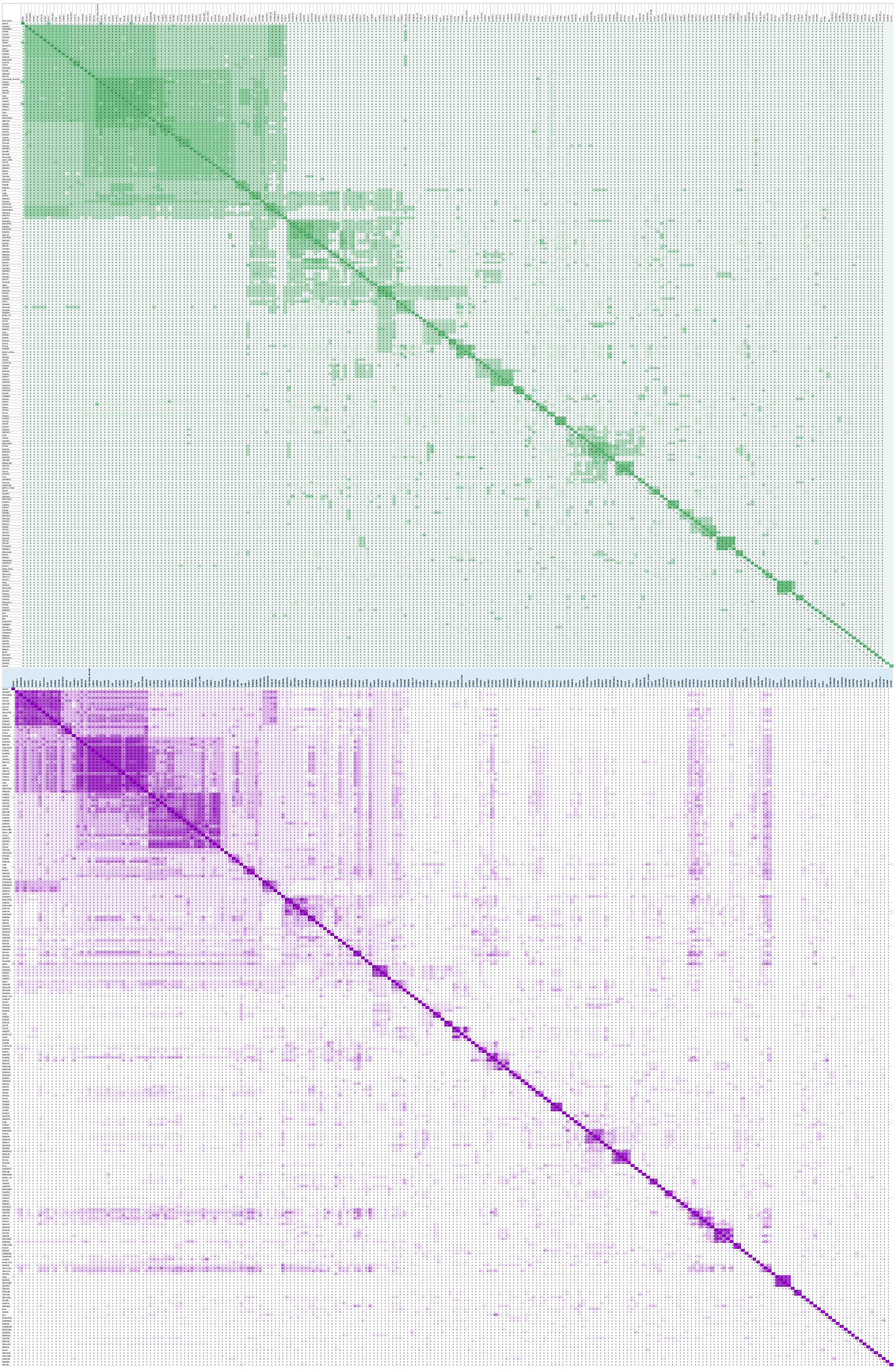

Figure S22. Spreadsheets generated from the (top) XPac analysis and (bottom) Crystal Packing Similarity analysis. The colors are darker for the higher number of matching molecules (scale 0-10 for XPac, see Table S13 with 10 denoting the matches along the diagonal, and scale 0-20 for Crystal Packing Similarity, with 25 denoting matches along the diagonal). These plots are in the separate Supporting Information Excel spreadsheet file, in which format they can be expanded to identify the structure labels. The order of the structures is the same in the two plots above, which was manually chosen for the XPac analysis.

Heat maps of the structural matches established using XPac and Mercury's Crystal Packing Similarity (Figure S22) were generated in Excel. Using these heat maps, it was possible to identify common structural sets, and match the most significant of these to the areas of interest seen in the dimer-based analysis carried out in Section S7.

(1) D'silva, E. D.; Narayan Rao, D.; Philip, R.; Butcher, R. J.; Rajnikant; Dharmaprakash, S. M. Second harmonic chalcone crystal: Synthesis, growth and characterization. *Physica B: Condensed Matter* **2011**, *406* (11), 2206-2210. DOI: https://doi.org/10.1016/j.physb.2011.03.032.

(2) Dong, M.-Y.; Mei, X.-D.; Li, Y.-F.; Zhang, L.-X.; Pan, W.-L.; Gao, Z.-L.; Ning, J. Crystal structure of (E)-3-(4-tert-butyl)phenyl)-1-(3-chlorophenyl)prop-2-en-1-one, C18H17ClO. *Zeitschrift für Kristallographie - New Crystal Structures* **2016**, *231* (1), 29-30. DOI: doi:10.1515/ncrs-2014-9076.

(3) Charvátová, H.; Císařová, I.; Štěpnička, P. Synthesis of Phosphanylferrocenecarboxamides Bearing Guanidinium Substituents and Their Application in the Palladium-Catalyzed Cross-Coupling of Boronic Acids with Acyl Chlorides. *European Journal of Inorganic Chemistry* **2017**, *2017* (2), 288-296, https://doi.org/10.1002/ejic.201600461. DOI: https://doi.org/10.1002/ejic.201600461 (acccessed 2021/10/06).

(4) Rabinovich, D.; Schmidt, G. M. J.; Shaked, Z. Topochemistry. Part XXXIV. Crystal and molecular structure of p'-bromochalcone. *Journal of the Chemical Society, Perkin Transactions 2* **1973**, (1), 33-37, 10.1039/P29730000033. DOI: 10.1039/P29730000033.

(5) Jungk, A. E.; Schmidt, G. M. J. Conformational studies. Part II. Crystal and molecular structures of 3-bromo-, 3-chloro-, and 4-bromo-2'-nitrochalcone. *Journal of the Chemical Society B: Physical Organic* **1970**, (0), 1427-1434, 10.1039/J29700001427. DOI: 10.1039/J29700001427.

(6) Jing, L.-H. (E)-1-(4-Nitrophenyl)-3-phenylprop-2-en-1-one. *Acta Crystallographica Section E* **2009**, *65* (10), o2510.

(7) Jing, L.-H. (E)-3-(4-Fluorophenyl)-1-phenyl-2-propen-1-one. *Acta Crystallographica Section E* **2009**, *65* (10), o2515.

(8) Arias-Ruiz, S. N.; Romero, N.; Lobato-Garcia, C. E.; Gomez-Rivera, A.; Mendoza, A. Second monoclinic form of (E)-3-(4-fluorophenyl)-1-phenylprop-2-en-1-one. *Acta Crystallographica Section E* **2013**, *69* (11), o1694-o1695.

(9) Rabinovich, D. Topochemistry. Part XXX. Crystal and molecular structures of chalcone. *Journal of the Chemical Society B: Physical Organic* **1970**, (0), 11-16, 10.1039/J29700000011. DOI: 10.1039/J29700000011.

(10) Wu, M. H.; Yang, X. H.; Zou, W. D.; Liu, W. J.; Li, C. Refinement of the crystal structure of (E)-1,3-diphenyl-2-propen-1-one, C15H12O. *Zeitschrift Fur Kristallographie-New Crystal Structures* **2006**, *221* (3), 323-324. DOI: 10.1524/ncrs.2006.0093.

(11) Sulpizio, C.; Roller, A.; Giester, G.; Rompel, A. Synthesis, structure, and antioxidant activity of methoxy- and hydroxyl-substituted 2'-aminochalcones. *Monatshefte für Chemie - Chemical Monthly* **2016**, *147* (10), 1747-1757. DOI: 10.1007/s00706-016-1812-9.

(12) Voigt-Martin, I. G.; Kolb, U.; Tenkovtsev, A. V.; Schollmeyer, D. (E)-3-(4-aminophenyl)-1-(4-bromophenyl)prop-2-en-1-one. *CSD Communication (Private Communication)* **2011**, *816983*.

(13) Voigt-Martin, I. G.; Kolb, U.; Tenkovtsev, A. V.; Schollmeyer, D. (E)-3-(4-aminophenyl)-1-(4-bromophenyl)prop-2-en-1-one. *CSD Communication (Private Communication)* **2016**, *818013*.

(14) Voigt-Martin, I. G.; Kolb, U.; Tenkovtsev, A. V.; Schollmeyer, D. (E)-3-(4-aminophenyl)-1-(4-chlorophenyl)prop-2-en-1-one. *CSD Communication (Private Communication)* **2011**, *819320*.

(15) Barsky, I.; Bernstein, J.; Stephens, P. W.; Stone, K. H.; Cheung, E.; Hickey, M. B.; Henck, J.-O. Disappearing and Reappearing Polymorphism in p-Methylchalcone. *Crystal Growth & Design* **2008**, *8* (1), 63-70. DOI: 10.1021/cg7007733.

(16) Rosli, M. M.; Patil, P. S.; Fun, H.-K.; Razak, I. A.; Dharmaprakash, S. M. (2E)-1-(3-Bromophenyl)-3-phenylprop-2-en-1-one. *Acta Crystallographica Section E* **2007**, *63* (5), o2501.

(17) Patil, P. S.; Chantrapromma, S.; Fun, H.-K.; Dharmaprakash, S. M.; Babu, H. B. R. 1-(4-Bromophenyl)-3-(3-methoxyphenyl)prop-2-en-1-one. *Acta Crystallographica Section E* **2007**, *63* (5), o2612.

(18) Jasinski, J. P.; Butcher, R. J.; Lakshmana, K.; Narayana, B.; Yathirajan, H. S. (2E)-3-(2-Chlorophenyl)-1-phenylprop-2-en-1-one. *Acta Crystallographica Section E* **2007**, *63* (12), o4715.

(19) Thiruvalluvar, A.; Subramanyam, M.; Butcher, R. J.; Adhikari, A. V.; Karabasanagouda, T. (2E)-3-(4-Chlorophenyl)-1-[4-(methylsulfanyl)phenyl]prop-2-en-1-one. *Acta Crystallographica Section E* **2007**, *63* (12), o4716.

(20) Ng, S.-L.; Patil, P. S.; Razak, I. A.; Fun, H.-K.; Dharmaprakash, S. M. 3-(3-Bromophenyl)-1-phenylprop-2-en-1-one. *Acta Crystallographica Section E* **2006**, *62* (3), o893-o895.
(21) Patil, P. S.; Teh, J. B.-J.; Fun, H.-K.; Razak, I. A.; Dharmaprakash, S. M. 3-(4-Methoxyphenyl)-1-(4-nitrophenyl)prop-2-en-1-one. *Acta Crystallographica Section E* **2006**, *62* (3), o896-o898.
(22) Rabinovich, D.; Shakked, Z. Optical induction in chiral crystals. I. The crystal and molecular structures of 4,4'-dimethylchalcone. *Acta Crystallographica Section B* **1974**, *30* (12), 2829-2834.
(23) Kobkeatthawin, T.; Chantrapromma, S.; Fun, H.-K. (E)-1-(4-Chlorophenyl)-3-[4-(diethylamino)phenyl]prop-2-en-1-oneThis paper is dedicated to His Majesty King Bhumibol Adulyadej of Thailand (King Rama IX) on the occasion of his 82th Birthday Anniversary which fell on December 5th, 2009. *Acta Crystallographica Section E* **2010**, *66* (2), o254-o255.
(24) Jasinski, J. P.; Pek, A. E.; Narayana, B.; Kamath, P. K.; Yathirajan, H. S. (E)-1-(4-Bromophenyl)-3-(2-methoxyphenyl)prop-2-en-1-one. *Acta Crystallographica Section E* **2010**, *66* (8), o1995.
(25) Jasinski, J. P.; Guild, C. J.; Narayana, B.; Nayak, P. S.; Yathirajan, H. S. (E)-3-(4-Methoxyphenyl)-1-[4-(piperidin-1-yl)phenyl]prop-2-en-1-one. *Acta Crystallographica Section E* **2010**, *66* (8), o1996.
(26) Hall, C. L.; Hamilton, V.; Potticary, J.; Cremeens, M. E.; Pridmore, N. E.; Sparkes, H. A.; D'Ambruoso, G. D.; Warren, S. D.; Matsumoto, M.; Hall, S. R. Crystal structures of three functionalized chalcones: 4'-dimethylamino-3-nitrochalcone, 3-dimethylamino-3'-nitrochalcone and 3'-nitrochalcone. *Acta Crystallographica Section E* **2020**, *76* (10), 1599-1604.
(27) Hall, C. L.; Guo, R.; Potticary, J.; Cremeens, M. E.; Warren, S. D.; Andrusenko, I.; Gemmi, M.; Zwijnenburg, M. A.; Sparkes, H. A.; Pridmore, N. E.; et al. Color Differences Highlight Concomitant Polymorphism of Chalcones. *Crystal Growth & Design* **2020**, *20* (10), 6346-6355. DOI: 10.1021/acs.cgd.0c00285.
(28) Ekbote, A.; Patil, P. S.; Maidur, S. R.; Chia, T. S.; Quah, C. K. Structure and nonlinear optical properties of (E)-1-(4-aminophenyl)-3-(3-chlorophenyl) prop-2-en-1-one: A promising new D-π-A-π-D type chalcone derivative crystal for nonlinear optical devices. *Journal of Molecular Structure* **2017**, *1129*, 239-247. DOI: https://doi.org/10.1016/j.molstruc.2016.09.077.
(29) Shoja, M. Crystal structure of 2'-hydroxy-4-methylchalcone, C16H14O2. *Zeitschrift für Kristallographie - New Crystal Structures* **1999**, *214* (2), 235-236. DOI: doi:10.1515/ncrs-1999-0243.
(30) Arshad, S.; Raveendran Pillai, R.; Zainuri, D. A.; Khalib, N. C.; Razak, I. A.; Armaković, S.; Armaković, S. J.; Renjith, R.; Panicker, C. Y.; Van Alsenoy, C. Synthesis, XRD crystal structure, spectroscopic characterization, local reactive properties using DFT and molecular dynamics simulations and molecular docking study of (E)-1-(4-bromophenyl)-3-(4-(trifluoromethoxy)phenyl)prop-2-en-1-one. *Journal of Molecular Structure* **2017**, *1137*, 419-430. DOI: https://doi.org/10.1016/j.molstruc.2017.02.045.
(31) Prabhu, S. R.; Jayarama, A.; Upadhyaya, V.; Bhat, K. S.; Ng, S. W. Structure and Characterization of a Novel Chalcone Crystal Having Nitro as an Acceptor Group. *Molecular Crystals and Liquid Crystals* **2015**, *607* (1), 200-214. DOI: 10.1080/15421406.2014.928434.
(32) Ravishankar, T.; Chinnakali, K.; Nanjundan, S.; Selvam, P.; Fun, H.-K.; Yu, X.-L. 1-(4-Chlorophenyl)-3-(4-hydroxyphenyl)prop-2-en-1-one. *Acta Crystallographica Section E* **2005**, *61* (2), o405-o407.
(33) Yesilyurt, F.; Aydin, A.; Gul, H. I.; Akkurt, M.; Ozcelik, N. D. Crystal structure of (2E)-1-(4-hydroxyphenyl)-3-(4-methoxyphenyl)prop-2-en-1-one. *European Journal of Chemistry; Vol 9, No 2 (2018): June 2018* **2018**. DOI: 10.5155/eurjchem.9.2.147-150.1733.
(34) Moorthi, S. S.; Chinnakali, K.; Nanjundan, S.; Unnithan, C. S.; Fun, H.-K.; Yu, X.-L. 1-(4-Bromophenyl)-3-(3-hydroxyphenyl)prop-2-en-1-one. *Acta Crystallographica Section E* **2005**, *61* (2), o483-o485.
(35) Köysal, Y.; Bülbül, H.; Özdemir, F.; İlhan, İ. Ö.; Dege, N. Crystal structure of 3-(4-methoxyphenyl)-1-(4-methylphenyl)prop-2-en-1-one, C17H16O2. *Zeitschrift für Kristallographie - New Crystal Structures* **2017**, *232* (4), 593-594. DOI: doi:10.1515/ncrs-2016-0357.
(36) Moorthi, S. S.; Chinnakali, K.; Nanjundan, S.; Selvam, P.; Fun, H.-K.; Yu, X.-L. 3-(3-Hydroxyphenyl)-1-(4-methoxyphenyl)prop-2-en-1-one. *Acta Crystallographica Section E* **2005**, *61* (3), o743-o745.
(37) Rajendraprasad, S.; Chidan Kumar, C. S.; Quah, C. K.; Chandraju, S.; Lokanath, N. K.; Naveen, S.; Warad, I. (E)-1-(3-Bromophenyl)-3-(3-fluorophenyl)prop-2-en-1-one. *IUCrData* **2017**, *2* (3), x170379.
(38) Wang, L.; Yang, W.; Zhang, D.-C. 1,3-Bis(4-chlorophenyl)prop-2-en-1-one. *Acta Crystallographica Section E* **2005**, *61* (9), o2820-o2822.
(39) Ng, S.-L.; Razak, I. A.; Fun, H.-K.; Shettigar, V.; Patil, P. S.; Dharmaprakash, S. M. 3-(4-Bromophenyl)-1-(4-chlorophenyl)prop-2-en-1-one. *Acta Crystallographica Section E* **2006**, *62* (6), o2175-o2177.

(40) Narayana, B.; Lakshmana, K.; Sarojini, B. K.; Yathirajan, H. S.; Bolte, M. 3-(2-Chlorophenyl)-1-(4-hydroxyphenyl)prop-2-en-1-one. *CSD Communication (Private Communication)* **2007**, *664910*.
(41) Narayana, B.; Salian, V. V.; Sarojini, B. K.; Jasinski, J. P. (2E)-1-(4-Chlorophenyl)-3-[4-(propan-2-yl)phenyl]prop-2-en-1-one. *Acta Crystallographica Section E* **2014**, *70* (8), o855.
(42) Rossi, M.; Caruso, F.; Crespi, E. J.; Pedersen, J. Z.; Nakano, G.; Duong, M.; McKee, C.; Lee, S.; Jiwrajka, M.; Caldwell, C.; et al. Probing antioxidant activity of 2′-hydroxychalcones: Crystal and molecular structures, in vitro antiproliferative studies and in vivo effects on glucose regulation. *Biochimie* **2013**, *95* (10), 1954-1963. DOI: https://doi.org/10.1016/j.biochi.2013.07.002.
(43) Qiu, X.-Y.; Ma, J.-L. (E)-3-(2-Chlorophenyl)-1-(4-methoxyphenyl)prop-2-en-1-one. *Acta Crystallographica Section E* **2006**, *62* (11), o5041-o5042.
(44) Harrison, W. T. A.; Yathirajan, H. S.; Narayana, B.; Sreevidya, T. V.; Sunil, K. (2E)-1-(4-Bromophenyl)-3-(4-nitrophenyl)prop-2-en-1-one. *Acta Crystallographica Section E* **2006**, *62* (11), o4829-o4831.
(45) Feng, M. C.; Tay, M. G.; Yeap, G. Y.; Norani, M. M.; Teoh, S. G.; Tiekink, E. R. T. Crystal structure of E-1-(2-hydroxyphenyl)-3-(3-hydroxyphenyl)-2- propen-1-one, C15H12O3. *Zeitschrift für Kristallographie - New Crystal Structures* **1999**, *214* (2), 205-206. DOI: doi:10.1515/ncrs-1999-0230.
(46) Gularyan, S. K.; Dobretsov, G. E.; Polyak, B. M.; Svetlichny, V. Y.; Zhukhlistova, N. E.; Krasovitskii, B. M.; Kormilova, L. I.; Zavodnik, V. E. 4-Dimethylaminochalcone as a fluorescent probe: quantum chemical calculations of its interaction with the environment. *Russian Chemical Bulletin* **2006**, *55* (10), 1737-1742. DOI: 10.1007/s11172-006-0481-y.
(47) Kupcewicz, B.; Jarzęcki, A. A.; Małecka, M.; Krajewska, U.; Rozalski, M. Cytotoxic activity of substituted chalcones in terms of molecular electronic properties. *Bioorganic & Medicinal Chemistry Letters* **2014**, *24* (17), 4260-4265. DOI: https://doi.org/10.1016/j.bmcl.2014.07.027.
(48) Kashino, S.; Tanaka, M.; Todorov, T. D. 2,2'-Dihydroxychalcone. *Acta Crystallographica Section E* **2001**, *57* (8), o735-o737.
(49) Jasinski, J. P.; Butcher, R. J.; Narayana, B.; Veena, K.; Yathirajan, H. S. (2E)-1-(3-Chlorophenyl)-3-phenylprop-2-en-1-one. *Acta Crystallographica Section E* **2010**, *66* (1), o157.
(50) Jasinski, J. P.; Butcher, R. J.; Narayana, B.; Veena, K.; Yathirajan, H. S. (2E)-3-(4-Bromophenyl)-1-(3-chlorophenyl)prop-2-en-1-one. *Acta Crystallographica Section E* **2010**, *66* (1), o158.
(51) Wang, L.; Ma, L.-Y.; Huang, Y.-L.; Zheng, B.-Y. (E)-3-[4-(Dimethylamino)phenyl]-1-(4-methylphenyl)prop-2-en-1-one. *Acta Crystallographica Section E* **2010**, *66* (1), o174.
(52) Ravishankar, T.; Chinnakali, K.; Nanjundan, S.; Radhakrishnan, S.; Usman, A.; Fun, H.-K. 1-(4-Methacryloyloxyphenyl)-3-(3-bromophenyl)prop-2-en-1-one. *Acta Crystallographica Section E* **2003**, *59* (2), o138-o140.
(53) Parthasarathi, V.; Praveen, V. N.; Thamotharan, S.; Vijayalakshmi, L.; Bhaskar, A. 1-(4-Hydroxyphenyl)-3-phenylprop-2-en-1-one. *Acta Crystallographica Section E* **2002**, *58* (1), o86-o87.
(54) Ng, S.-L.; Razak, I. A.; Fun, H.-K.; Patil, P. S.; Dharmaprakash, S. M. 4'-Fluorochalcone. *Acta Crystallographica Section E* **2006**, *62* (7), o2897-o2899.
(55) Anuradha, N.; Thiruvalluvar, A.; Mahalinga, M.; Butcher, R. J. (E)-3-(4-Fluorophenyl)-1-[4-(methylsulfanyl)phenyl]prop-2-en-1-one. *Acta Crystallographica Section E* **2008**, *64* (11), o2118-o2119.
(56) Li, H.; Sarojini, B. K.; Raj, C. G. D.; Madhu, L. N.; Yathirajan, H. S. (E)-3-(3-Bromophenyl)-1-(4-methylphenyl)prop-2-en-1-one. *Acta Crystallographica Section E* **2008**, *64* (11), o2238.
(57) Jasinski, J. P.; Butcher, R. J.; Siddaraju, B. P.; Narayana, B.; Yathirajan, H. S. (2E)-3-[4-(Dimethylamino)phenyl]-1-(4-fluorophenyl)prop-2-en-1-one. *Acta Crystallographica Section E* **2011**, *67* (2), o313-o314.
(58) Agilandeshwari, R.; Meenatchi, V.; Meenakshisundaram, S. P. Synthesis, growth, structure and characterization of chalcone crystal: A novel organic NLO material. *Journal of Molecular Structure* **2016**, *1118*, 356-366. DOI: https://doi.org/10.1016/j.molstruc.2016.02.099.
(59) Salinas-Ortega, I.; Ocayo, F.; Santos, J. C.; Trujillo, A.; Escobar, C. A. Synthesis, characterization and crystal structure of 4′-ethynylflavanone and its chalcone precursor. *Journal of Molecular Structure* **2017**, *1128*, 361-367. DOI: https://doi.org/10.1016/j.molstruc.2016.09.005.
(60) Turowska-Tyrk, I.; Grześniak, K.; Trzop, E.; Zych, T. Monitoring structural transformations in crystals. Part 4. Monitoring structural changes in crystals of pyridine analogs of chalcone during [2+2]-photodimerization and possibilities of the reaction in hydroxy derivatives. *Journal of Solid State Chemistry* **2003**, *174* (2), 459-465. DOI: https://doi.org/10.1016/S0022-4596(03)00304-9.

(61) Jennings, G.; Smith, M. D.; Kuang, S.-M.; Mark Hodges, L.; Tyrell, J.; Thomas Williamson, R.; Seaton, P. Orthorhombic Polymorphs of 1-Phenyl-3-(3-Hydroxyphenyl)-2-Propen-1-One. *Journal of Chemical Crystallography* **2012**, *42* (2), 159-164. DOI: 10.1007/s10870-011-0218-0.
(62) Zainuri, D. A.; Arshad, S.; Khalib, N. C.; Razak, I. A.; Pillai, R. R.; Sulaiman, S. F.; Hashim, N. S.; Ooi, K. L.; Armaković, S.; Armaković, S. J.; et al. Synthesis, XRD crystal structure, spectroscopic characterization (FT-IR, 1H and 13C NMR), DFT studies, chemical reactivity and bond dissociation energy studies using molecular dynamics simulations and evaluation of antimicrobial and antioxidant activities of a novel chalcone derivative, (E)-1-(4-bromophenyl)-3-(4-iodophenyl)prop-2-en-1-one. *Journal of Molecular Structure* **2017**, *1128*, 520-533. DOI: https://doi.org/10.1016/j.molstruc.2016.09.022.
(63) Wang, L.; Zhang, Y.; Lu, C.-R.; Zhang, D.-C. 1-(4-Bromophenyl)-3-(4-methylphenyl)prop-2-en-1-one Supported by the Key Subject Programme of Jiangsu Province (grant No. S1109001). *Acta Crystallographica Section C* **2004**, *60* (9), o696-o698.
(64) Moorthi, S. S.; Chinnakali, K.; Nanjundan, S.; Santhi, R.; Fun, H.-K. 1-(4-Aminophenyl)-3-(3-bromophenyl)prop-2-en-1-one. *Acta Crystallographica Section E* **2005**, *61* (10), o3514-o3516.
(65) Abonia, R.; Cabrera, L.; Quiroga, J.; Insuasty, B.; Moreno-Fuquen, R.; Kennedy, A. R. (E)-1-(2-Aminophenyl)-3-(4-chlorophenyl)prop-2-en-1-one. *Molbank* **2016**, *2016* (4). DOI: 10.3390/M911.
(66) Fun, H.-K.; Chantrapromma, S.; Patil, P. S.; Dharmaprakash, S. M. (E)-1-(3-Bromophenyl)-3-(4-ethoxyphenyl)prop-2-en-1-one. *Acta Crystallographica Section E* **2008**, *64* (7), o1356-o1357.
(67) Butcher, R. J.; Jasinski, J. P.; Yathirajan, H. S.; Veena, K.; Narayana, B. (2E)-1-(4-Methylphenyl)-3-(4-nitrophenyl)prop-2-en-1-one. *Acta Crystallographica Section E* **2007**, *63* (9), o3680.
(68) Butcher, R. J.; Jasinski, J. P.; Yathirajan, H. S.; Narayana, B.; Mithun, A. (2E)-1-(4-Bromophenyl)-3-[4-(methylsulfanyl)phenyl]prop-2-en-1-one. *Acta Crystallographica Section E* **2007**, *63* (9), o3731-o3732.
(69) Butcher, R. J.; Jasinski, J. P.; Yathirajan, H. S.; Narayana, B.; Veena, K. (E)-3-(4-Fluorophenyl)-1-(4-methylphenyl)prop-2-en-1-one. *Acta Crystallographica Section E* **2007**, *63* (9), o3833.
(70) Fun, H.-K.; Patil, P. S.; Dharmaprakash, S. M.; Chantrapromma, S. (E)-1-(4-Bromophenyl)-3-(2-chlorophenyl)prop-2-en-1-one. *Acta Crystallographica Section E* **2008**, *64* (8), o1464.
(71) Zaini, M. F.; Arshad, S.; Thanigaimani, K.; Khalib, N. C.; Zainuri, D. A.; Abdullah, M.; Razak, I. A. New halogenated chalcones: Synthesis, crystal structure, spectroscopic and theoretical analyses for third-order nonlinear optical properties. *Journal of Molecular Structure* **2019**, *1195*, 606-619. DOI: https://doi.org/10.1016/j.molstruc.2019.05.122.
(72) Zheng-Dong, L.; Liang-Ren, H.; Gen-Bo, S.; Hong-Jun, W. 4-Bromo-4'-methoxy-chalcone. *Jiegou Huaxue* **1992**, *11*, 1.
(73) Hurst, D. P.; Titterington, J. A.; Van Wier, S. P.; Whitwood, A. C.; Wood, N. J. 1-(4-methoxyphenyl)-3-phenylprop-2-en-1-one. *CSD Communication (Private Communication)* **2018**, *1842224*.
(74) Fun, H.-K.; Chia, T. S.; Sapnakumari, M.; Narayana, B.; Sarojini, B. K. (E)-3-(4-Chlorophenyl)-1-(4-fluorophenyl)prop-2-en-1-one. *Acta Crystallographica Section E* **2012**, *68* (3), o629.
(75) Su, G. B.; Li, Z. D.; Pan, R. F.; Wu, D. X.; Yang, T. Q. Growth of Large Crystals of Pentaerythritol and Trishydroxymethylaminomethone. *Journal of Crystal Growth* **1992**, *119* (3-4), 368-370.
(76) Ng, S.-L.; Shettigar, V.; Razak, I. A.; Fun, H.-K.; Patil, P. S.; Dharmaprakash, S. M. 1,3-Bis(4-bromophenyl)prop-2-en-1-one. *Acta Crystallographica Section E* **2006**, *62* (4), o1421-o1423.
(77) Yang, W.; Wang, L.; Zhang, D. X-ray structure analysis of 1-(4-bromophenyl)-3-(4-chlorophenyl)-prop-2-en-1-one. *Journal of Chemical Crystallography* **2006**, *36* (3), 195-198. DOI: 10.1007/s10870-005-9046-4.
(78) Braun, R. U.; Ansorge, M.; Müller, T. J. J. Coupling–Isomerization Synthesis of Chalcones. *Chemistry – A European Journal* **2006**, *12* (35), 9081-9094, https://doi.org/10.1002/chem.200600530. DOI: https://doi.org/10.1002/chem.200600530 (acccessed 2021/10/13).
(79) Wallet, J. C.; Molins, E.; Miravitlles, C. 2'-Hydroxy-2-methoxychalcone. *Acta Crystallographica Section C* **1995**, *51* (1), 123-125.
(80) Fun, H.-K.; Jebas, S. R.; Patil, P. S.; Dharmaprakash, S. M. (E)-1-(4-Chlorophenyl)-3-(4-methylphenyl)prop-2-en-1-one. *Acta Crystallographica Section E* **2008**, *64* (6), o1038.
(81) Hussain, T.; Siddiqui, H. L.; Zia-ur-Rehman, M.; Masoom Yasinzai, M.; Parvez, M. Anti-oxidant, anti-fungal and anti-leishmanial activities of novel 3-[4-(1H-imidazol-1-yl) phenyl]prop-2-en-1-ones. *European Journal of Medicinal Chemistry* **2009**, *44* (11), 4654-4660. DOI: https://doi.org/10.1016/j.ejmech.2009.06.038.
(82) Ramos, R. R.; da Silva, C. C.; Guimaraes, F. F.; Martins, F. T. Polymorphism and conformerism in chalcones. *Crystengcomm* **2016**, *18* (12), 2144-2154, Article. DOI: 10.1039/c5ce02591e.

(83) Harrison, W. T. A.; Yathirajan, H. S.; Sarojini, B. K.; Narayana, B.; Indira, J. 1-(4-Chlorophenyl)-3-(4-methoxyphenyl)prop-2-en-1-one. *Acta Crystallographica Section E* **2006**, *62* (4), o1647-o1649.
(84) Jasinski, J. P.; Butcher, R. J.; Narayana, B.; Samshuddin, S.; Yathirajan, H. S. A monoclinic polymorph of 1-(4-chlorophenyl)-3-(4-methoxyphenyl)prop-2-en-1-one. *Acta Crystallographica Section E* **2010**, *66* (2), o269-o270.
(85) Rosli, M. M.; Patil, P. S.; Fun, H.-K.; Razak, I. A.; Dharmaprakash, S. M. 3-(4-Bromophenyl)-1-(4-nitrophenyl)prop-2-en-1-one. *Acta Crystallographica Section E* **2006**, *62* (4), o1466-o1468.
(86) Shettigar, V.; Rosli, M. M.; Fun, H.-K.; Razak, I. A.; Patil, P. S.; Dharmaprakash, S. M. 1-(4-Bromophenyl)-3-(4-methoxyphenyl)prop-2-en-1-one. *Acta Crystallographica Section E* **2006**, *62* (9), o4128-o4129.
(87) Arshad, M. N.; Al-Dies, A.-A. M.; Asiri, A. M.; Khalid, M.; Birinji, A. S.; Al-Amry, K. A.; Braga, A. A. C. Synthesis, crystal structures, spectroscopic and nonlinear optical properties of chalcone derivatives: A combined experimental and theoretical study. *Journal of Molecular Structure* **2017**, *1141*, 142-156. DOI: https://doi.org/10.1016/j.molstruc.2017.03.090.
(88) Slabber, C. A.; Grimmer, C. D.; Munro, O. Q.; Robinson, R. S. Synthesis, Characterization, and Crystal Structure of (2E)-3-(4-Fluorophenyl)-1-(2-hydroxyphenyl)prop-2-en-1-one. *Journal of Chemical Crystallography* **2018**, *48* (4), 213-219. DOI: 10.1007/s10870-018-0732-4.
(89) Fun, H.-K.; Jebas, S. R.; Patil, P. S.; D'Silva, E. D.; Dharmaprakash, S. M. (E)-1-(4-Fluorophenyl)-3-(4-methylphenyl)prop-2-en-1-one. *Acta Crystallographica Section E* **2008**, *64* (5), o935.
(90) Whitwood, A. C.; Pugh, D. S.; Wood, N. J.; Mills, A. L. F.; Aspell, A. M.; Coyne, E.; Hill, I. A.; Moore, O.; Latter, R. C. 1-(3-methylphenyl)-3-(3-nitrophenyl)prop-2-en-1-one. *CSD Communication (Private Communication)* **2019**, *1900322*.
(91) Hurst, D. P.; Titterington, J. A.; Van Wier, S. P.; Whitwood, A. C.; Wood, N. J. 3-(4-methoxyphenyl)-1-phenylprop-2-en-1-one. *CSD Communication (Private Communication)* **2018**, *1842222*.
(92) Niu, C.; Tuerxuntayi, A.; Li, G.; Kabas, M.; Dong, C.-Z.; Aisa, H. A. Design, synthesis and bioactivity of chalcones and its analogues. *Chinese Chemical Letters* **2017**, *28* (7), 1533-1538. DOI: https://doi.org/10.1016/j.cclet.2017.03.018.
(93) Braun, R.; Muller, T. J. J.; Polborn, K. 4-((1E)-3-(4-(diethylamino)phenyl)-3-oxoprop-1-enyl)benzonitrile. *CSD Communication (Private Communication)* **2005**, *273749*.
(94) Harrison, W. T. A.; Yathirajan, H. S.; Mithun, A.; Narayana, B.; Sarojini, B. K. 3-[4-(Methylsulfanyl)phenyl]-1-(4-nitrophenyl)prop-2-en-1-one. *Acta Crystallographica Section E* **2006**, *62* (10), o4508-o4509.
(95) Rosli, M. M.; Patil, P. S.; Fun, H.-K.; Razak, I. A.; Dharmaprakash, S. M. (2E)-3-[4-(Dimethylamino)phenyl]-1-(3-nitrophenyl)prop-2-en-1-one. *Acta Crystallographica Section E* **2007**, *63* (5), o2692.
(96) Dutkiewicz, G.; Veena, K.; Narayana, B.; Yathirajan, H. S.; Kubicki, M. (2E)-1-(4-Bromophenyl)-3-(4-fluorophenyl)prop-2-en-1-one. *Acta Crystallographica Section E* **2010**, *66* (5), o1243-o1244.
(97) Peng, J.; Xu, H.; Li, Z.; Zhang, Y.; Wu, J. 1-(4-Aminophenyl)-3-[2-(trifluoromethyl)phenyl]prop-2-en-1-one. *Acta Crystallographica Section E* **2010**, *66* (5), o1156-o1157.
(98) Ekbote, A.; Patil, P. S.; Maidur, S. R.; Chia, T. S.; Quah, C. K. Structural, third-order optical nonlinearities and figures of merit of (E)-1-(3-substituted phenyl)-3-(4-fluorophenyl) prop-2-en-1-one under CW regime: New chalcone derivatives for optical limiting applications. *Dyes and Pigments* **2017**, *139*, 720-729. DOI: https://doi.org/10.1016/j.dyepig.2017.01.002.
(99) Teh, J. B.-J.; Patil, P. S.; Fun, H.-K.; Razak, I. A.; Dharmaprakash, S. M. 3-(3-Bromophenyl)-1-(4-bromophenyl)prop-2-en-1-one. *Acta Crystallographica Section E* **2006**, *62* (6), o2399-o2400.
(100) Hamilton, V.; Harris, C.; Hall, C. L.; Potticary, J.; Cremeens, M. E.; D'Ambruoso, G. D.; Matsumoto, M.; Warren, S. D.; Pridmore, N. E.; Sparkes, H. A.; et al. Structural effects of halogen bonding in iodochalcones. *Acta Crystallographica Section B* **2021**, *77* (3), 347-356.
(101) D'Silva, E. D. 1-(4-bromophenyl)-3-(4-(methylsulfanyl)phenyl)prop-2-en-1-one 1-(4-chlorophenyl)-3-(4-(methylsulfanyl)phenyl)prop-2-en-1-one. *CSD Communication (Private Communication)* **2016**, *875419*.
(102) Prabhu, S. R.; Jayarama, A.; Chandrasekharan, K.; Upadhyaya, V.; Ng, S. W. Synthesis, growth, structural characterization, Hirshfeld analysis and nonlinear optical studies of a methyl substituted chalcone. *Journal of Molecular Structure* **2017**, *1136*, 244-252. DOI: https://doi.org/10.1016/j.molstruc.2017.01.069.
(103) Arshad, S.; Pillai, R. R.; Zainuri, D. A.; Khalib, N. C.; Razak, I. A.; Armaković, S.; Armaković, S. J.; Panicker, C. Y.; Van Alsenoy, C. Synthesis, crystal structure, Hirshfeld surface analysis, spectroscopic characterization, reactivity study by DFT and MD approaches and molecular docking study of a novel chalcone derivative. *Journal of Molecular Structure* **2017**, *1135*, 234-246. DOI: https://doi.org/10.1016/j.molstruc.2017.01.080.

(104) Kumara, K.; Jyothi, M.; Zabiulla; Shivalingegowda, N.; Khanum, S. A.; Krishnappagowda, L. N. Synthesis, characterization, crystal structure and Hirshfeld surface analysis of 1-(4-ethoxyphenyl)-3-(4-methylphenyl) prop-2en-1-one. *Chemical Data Collections* **2017**, *9-10*, 152-163. DOI: https://doi.org/10.1016/j.cdc.2017.06.003.
(105) Harrison, W. T. A.; Yathirajan, H. S.; Anilkumar, H. G.; Sarojini, B. K.; Narayana, B. 1-(4-Fluorophenyl)-3-(4-methoxyphenyl)prop-2-en-1-one. *Acta Crystallographica Section E* **2006**, *62* (8), o3251-o3253.
(106) Goswami, P.; Talukdar, M.; Bora, T. C.; Phukan, P.; Sarma, J. C. Chlorotrimethylsilane catalyzed synthesis of 1,3-diphenyl-2-propenones and their antimicrobial activities. *Comptes Rendus Chimie* **2013**, *16* (5), 442-450. DOI: https://doi.org/10.1016/j.crci.2012.11.014.
(107) Masuyama, Y.; Takamura, W.; Suzuki, N. Tin(II) Chloride Mediated Coupling Reactions between Alkynes and Aldehydes. *European Journal of Organic Chemistry* **2013**, *2013* (35), 8033-8038, https://doi.org/10.1002/ejoc.201301189. DOI: https://doi.org/10.1002/ejoc.201301189 (acccessed 2021/10/13).
(108) Zaini, M. F.; Razak, I. A.; Anis, M. Z.; Arshad, S. Crystal structure, Hirshfeld surface analysis and DFT studies of (E)-1-(4-bromophenyl)-3-(3-fluorophenyl)prop-2-en-1-one. *Acta Crystallographica Section E* **2019**, *75* (1), 58-63.
(109) Wong, Q. A.; Chia, T. S.; Kwong, H. C.; Chidan Kumar, C. S.; Quah, C. K.; Arafath, M. A. Crystal structure and Hirshfeld surface analysis of a chalcone derivative: (E)-3-(4-fluorophenyl)-1-(4-nitrophenyl)prop-2-en-1-one. *Acta Crystallographica Section E* **2019**, *75* (1), 53-57.
(110) Li, H.; Kamath, K. P.; Narayana, B.; Yathirajan, H. S.; Harrison, W. T. A. (2E)-3-(3-Bromophenyl)-1-(4-chlorophenyl)prop-2-en-1-one: a non-merohedral twin. *Acta Crystallographica Section E* **2009**, *65* (8), o1915.
(111) Dutkiewicz, G.; Chidan Kumar, C. S.; Yathirajan, H. S.; Narayana, B.; Kubicki, M. (E)-3-(Biphenyl-4-yl)-1-(3-bromophenyl)prop-2-en-1-one. *Acta Crystallographica Section E* **2009**, *65* (11), o2856-o2857.
(112) Toda, F.; Tanaka, K.; Kato, M. Stereoselective photodimerisation of chalcones in the molten state. *Journal of the Chemical Society-Perkin Transactions 1* **1998**, (7), 1315-1318.
(113) Hurst, D. P.; Titterington, J. A.; Van Wier, S. P.; Whitwood, A. C.; Wood, N. J. 1-(4-methylphenyl)-3-phenylprop-2-en-1-one. *CSD Communication (Private Communication)* **2018**, *1842229*.
(114) Fun, H.-K.; Farhadikoutenaei, A.; Narayana, B.; Nayak, P. S.; Sarojini, B. K. (2E)-3-(2-Fluorophenyl)-1-(4-fluorophenyl)prop-2-en-1-one. *Acta Crystallographica Section E* **2012**, *68* (9), o2658.
(115) Zhao, P.-H.; Hao, E.-J.; Liu, Y.-Q.; Zhao, G.-Z. (E)-1-Phenyl-3-[4-(trifluoromethyl)phenyl]prop-2-en-1-one. *Acta Crystallographica Section E* **2012**, *68* (9), o2750.
(116) Qiu, X.-Y.; Yang, S.-L.; Liu, W.-S.; Zhu, H.-L. (E)-3-(4-Hydroxyphenyl)-1-(4-methoxyphenyl)prop-2-en-1-one. *Acta Crystallographica Section E* **2006**, *62* (8), o3324-o3325.
(117) Qiu, X.-Y.; Luo, Z.-G.; Yang, S.-L.; Liu, W.-S. (E)-1-(4-Chlorophenyl)-3-(4-fluorophenyl)prop-2-en-1-one. *Acta Crystallographica Section E* **2006**, *62* (8), o3525-o3526.
(118) Teh, J. B.-J.; Patil, P. S.; Fun, H.-K.; Razak, I. A.; Dharmaprakash, S. M. 1-(4-Aminophenyl)-3-(4-chlorophenyl)prop-2-en-1-one. *Acta Crystallographica Section E* **2006**, *62* (11), o5150-o5151.
(119) Fun, H.-K.; Patil, P. S.; Dharmaprakash, S. M.; Chantrapromma, S. 3-(4-Chlorophenyl)-1-(2-hydroxyphenyl)prop-2-en-1-one. *Acta Crystallographica Section E* **2007**, *63* (2), o561-o562.
(120) Fun, H.-K.; Patil, P. S.; Dharmaprakash, S. M.; Chantrapromma, S. 1-(4-Bromophenyl)-3-(4-ethoxyphenyl)prop-2-en-1-one. *Acta Crystallographica Section E* **2008**, *64* (8), o1540-o1541.
(121) Kant, R.; Maiti, B.; Awasthi, S. K.; Agarwal, A. Hydrogen Bonding Patterns and DFT Studies of (4-Acetylphenyl)amino 2,2-Dimethylpropanoate and (E)-1-(4-Aminophenyl)-3-[4-(dimethylamino)phenyl]prop-2-en-1-one. *Journal of Chemical Crystallography* **2014**, *44* (8), 421-434. DOI: 10.1007/s10870-014-0533-3.
(122) Jasinski, J. P.; Butcher, R. J.; Veena, K.; Narayana, B.; Yathirajan, H. S. (2E)-1-(2-Bromophenyl)-3-(4-chlorophenyl)prop-2-en-1-one. *Acta Crystallographica Section E* **2010**, *66* (7), o1638.
(123) Jasinski, J. P.; Butcher, R. J.; Veena, K.; Narayana, B.; Yathirajan, H. S. (2E)-1-(2-Bromophenyl)-3-(4-methoxyphenyl)prop-2-en-1-one. *Acta Crystallographica Section E* **2010**, *66* (7), o1661.
(124) Jasinski, J. P.; Butcher, R. J.; Veena, K.; Narayana, B.; Yathirajan, H. S. (2E)-1-(2-Bromophenyl)-3-(4-bromophenyl)prop-2-en-1-one. *Acta Crystallographica Section E* **2010**, *66* (7), o1701.
(125) Serdiuk, I. E.; Wera, M.; Roshal, A. D. Structural and Spectral Features of 4′-Substituted 2′-Hydroxychalcones in Solutions and Crystals: Spectroscopic and Theoretical Investigations. *The Journal of Physical Chemistry A* **2018**, *122* (8), 2030-2038. DOI: 10.1021/acs.jpca.7b10361.

(126) Zaini, M. F.; Arshad, S.; Ibrahim, A. R.; Che Khalib, N.; Zainuri, D. A. Structural Properties and Quantum Chemical Analysis on a New Chalcone Derivative of (E)-3-(4-bromophenyl)-1-(4-fluorophenyl)prop-2-en-1-one. *Journal of Physics: Conference Series* **2018**, *1083*, 012047. DOI: 10.1088/1742-6596/1083/1/012047.
(127) Whitwood, A. C.; Pugh, D. S.; Wood, N. J.; Mills, A. L. F.; Aspell, A. M.; Coyne, E.; Hill, I. A.; Moore, O.; Latter, R. C. 3-(4-bromophenyl)-1-(4-fluorophenyl)prop-2-en-1-one. *CSD Communication (Private Communication)* **2019**, *1900323*.
(128) Fun, H.-K.; Chantrapromma, S.; Patil, P. S.; D'Silva, E. D.; Dharmaprakash, S. M. (E)-3-(4-Methylphenyl)-1-(4-nitrophenyl)prop-2-en-1-one. *Acta Crystallographica Section E* **2008**, *64* (6), o954-o955.
(129) Fun, H.-K.; Chantrapromma, S.; Patil, P. S.; Dharmaprakash, S. M. (E)-3-(2-Chlorophenyl)-1-(4-nitrophenyl)prop-2-en-1-one. *Acta Crystallographica Section E* **2008**, *64* (6), o958-o959.
(130) Fun, H.-K.; Kia, R.; Patil, P. S.; Dharmaprakash, S. M.; Razak, I. A. (E)-1-(4-Aminophenyl)-3-(2-chlorophenyl)prop-2-en-1-one. *Acta Crystallographica Section E* **2008**, *64* (10), o2014-o2015.
(131) Ovchinnikova, I. G.; Valova, M. S.; Matochkina, E. G.; Kodess, M. I.; Tumashov, A. A.; Slepukhin, P. A.; Fedorova, O. V.; Rusinov, G. L.; Charushin, V. N. Specific features of heterocyclization of (E)-3-(2-ethoxyphenyl)-1-phenylprop-2-en-1-one with aminoazoles. *Russian Chemical Bulletin* **2014**, *63* (7), 1552-1576. DOI: 10.1007/s11172-014-0635-2.
(132) Spruce, K. J.; Hall, C. L.; Potticary, J.; Pridmore, N. E.; Cremeens, M. E.; D'Ambruoso, G. D.; Matsumoto, M.; Warren, G. I.; Warren, S. D.; Hall, S. R. Crystal structure and Hirshfeld surface analysis of (E)-3-(3-iodophenyl)-1-(4-iodophenyl)prop-2-en-1-one. *Acta Crystallographica Section E* **2020**, *76* (1), 72-76.
(133) Jasinski, J. P.; Butcher, R. J.; Narayana, B.; Veena, K.; Yathirajan, H. S. (2E)-1-(3-Chlorophenyl)-3-(4-chlorophenyl)prop-2-en-1-one. *Acta Crystallographica Section E* **2009**, *65* (11), o2641-o2642.
(134) Guo, H.-M.; Liu, L.-Q.; Yang, J.; Jian, F.-F. 3-(4-Methoxyphenyl)-1-(2-nitrophenyl)prop-2-en-1-one. *Acta Crystallographica Section E* **2009**, *65* (12), o3117.
(135) Dimmock, J. R.; Kandepu, N. M.; Hetherington, M.; Quail, J. W.; Pugazhenthi, U.; Sudom, A. M.; Chamankhah, M.; Rose, P.; Pass, E.; Allen, T. M.; et al. Cytotoxic Activities of Mannich Bases of Chalcones and Related Compounds. *Journal of Medicinal Chemistry* **1998**, *41* (7), 1014-1026. DOI: 10.1021/jm970432t.
(136) Fischer, A.; Yathirajan, H. S.; Ashalatha, B. V.; Narayana, B.; Sarojini, B. K. (2E)-3-(Biphenyl-4-yl)-1-(4-methoxyphenyl)prop-2-en-1-one. *Acta Crystallographica Section E* **2007**, *63* (3), o1349-o1350.
(137) Fischer, A.; Yathirajan, H. S.; Ashalatha, B. V.; Narayana, B.; Sarojini, B. K. (2E)-3-(Biphenyl-4-yl)-1-phenylprop-2-en-1-one. *Acta Crystallographica Section E* **2007**, *63* (3), o1357-o1358.
(138) Fischer, A.; Yathirajan, H. S.; Ashalatha, B. V.; Narayana, B.; Sarojini, B. K. (2E)-3-(Biphenyl-4-yl)-1-(4-bromophenyl)prop-2-en-1-one. *Acta Crystallographica Section E* **2007**, *63* (3), o1355-o1356.
(139) Fischer, A.; Yathirajan, H. S.; Ashalatha, B. V.; Narayana, B.; Sarojini, B. K. (2E)-3-(Biphenyl-4-yl)-1-(4-chlorophenyl)prop-2-en-1-one. *Acta Crystallographica Section E* **2007**, *63* (3), o1353-o1354.
(140) Thiruvalluvar, A.; Subramanyam, M.; Butcher, R. J.; Karabasanagouda, T.; Adhikari, A. V. (E)-1-[4-(Methylsulfanyl)phenyl]-3-phenylprop-2-en-1-one. *Acta Crystallographica Section E* **2008**, *64* (7), o1263.
(141) Abbas, A.; Khawar Rauf, M.; Bolte, M.; Hasan, A. (E)-3-[4-(Pentyloxy)phenyl]-1-phenylprop-2-en-1-one. *Acta Crystallographica Section E* **2009**, *65* (6), o1280.
(142) Guo, H.-M. 3-(2-Fluorophenyl)-1-(4-methoxyphenyl)prop-2-en-1-one. *Acta Crystallographica Section E* **2009**, *65* (12), o3013.
(143) Horkaew, J.; Chantrapromma, S.; Saewan, N.; Fun, H.-K. (E)-3-(4-Ethoxyphenyl)-1-(2-hydroxyphenyl)prop-2-en-1-one. *Acta Crystallographica Section E* **2010**, *66* (9), o2346-o2347.
(144) Potticary, J.; Hall, C.; Hamilton, V.; Sparkes, H. A.; Cremeens, M. E.; D'Ambruoso, G. D.; Matsumoto, M.; Warren, S. D.; Harr, S. R. 1-(4-chlorophenyl)-3-(4-iodophenyl)prop-2-en-1-one. *CSD Communication (Private Communication)* **2020**, *2044441*.
(145) Li, Z.; Pa, F.; Su, G. Structure of 4-bromochalcone. *Acta Crystallographica Section C* **1992**, *48* (4), 712-714.
(146) Yathirajan, H. S.; Sreevidya, T. V.; Narayana, B.; Sarojini, B. K.; Bolte, M. (2E)-3-(4-Butoxyphenyl)-1-(4-chlorophenyl)prop-2-en-1-one. *Acta Crystallographica Section E* **2006**, *62* (12), o5923-o5924.
(147) Michelini, L. J.; Castro, M. R. C.; Custodio, J. M. F.; Naves, L. F. N.; Vaz, W. F.; Lobón, G. S.; Martins, F. T.; Perez, C. N.; Napolitano, H. B. A novel potential anticancer chalcone: Synthesis, crystal structure and cytotoxic assay. *Journal of Molecular Structure* **2018**, *1168*, 309-315. DOI: https://doi.org/10.1016/j.molstruc.2018.05.010.
(148) Hurst, D. P.; Titterington, J. A.; Van Wier, S. P.; Whitwood, A. C.; Wood, N. J. 1-(4-fluorophenyl)-3-(4-nitrophenyl)prop-2-en-1-one. *CSD Communication (Private Communication)* **2018**, *1842230*.

(149) Butcher, R. J.; Jasinski, J. P.; Narayana, B.; Lakshmana, K.; Yathirajan, H. S. (2E)-3-(4-Fluorophenyl)-1-(3-hydroxyphenyl)prop-2-en-1-one. *Acta Crystallographica Section E* **2007**, *63* (8), o3586.
(150) Butcher, R. J.; Jasinski, J. P.; Narayana, B.; Lakshmana, K.; Yathirajan, H. S. (2E)-1-(3-Hydroxyphenyl)-3-(4-methylphenyl)prop-2-en-1-one. *Acta Crystallographica Section E* **2007**, *63* (8), o3660.
(151) Butcher, R. J.; Jasinski, J. P.; Yathirajan, H. S.; Lakshmana, K.; Narayana, B. (2E)-3-(4-Chlorophenyl)-1-(3-hydroxyphenyl)prop-2-en-1-one. *Acta Crystallographica Section E* **2007**, *63* (8), o3661.
(152) Yeap, G.-Y.; Feng, M.-C.; Ng, A.-C.; Tiekink, E. R. T. Crystal structure of 3-(2-hydroxyphenyl)-2-propene-1-one, C15H12O2. *Zeitschrift für Kristallographie - Crystalline Materials* **1996**, *211* (12), 949-950. DOI: doi:10.1524/zkri.1996.211.12.949.
(153) Ahmad, N.; Siddiqui, H. L.; Zia-ur-Rehman, M.; Parvez, M. (E)-3-(3-Chlorophenyl)-1-(4-methoxyphenyl)prop-2-en-1-one. *Acta Crystallographica Section E* **2010**, *66* (6), o1346-o1347.
(154) Pan, Z.-f. (E)-3-(4-Fluorophenyl)-1-(2-nitrophenyl)prop-2-en-1-one. *Acta Crystallographica Section E* **2010**, *66* (6), o1414.
(155) Sarojini, B. K.; Yathirajan, H. S.; Lakshmana, K.; Narayana, B.; Bolte, M. (2E)-3-(2-Chlorophenyl)-1-(3-nitrophenyl)prop-2-en-1-one. *Acta Crystallographica Section E* **2007**, *63* (7), o3211.
(156) Jasinski, J. P.; Butcher, R. J.; Yathirajan, H. S.; Sarojini, B. K.; Musthafa Khaleel, V. (2E)-3-(4-Chlorophenyl)-1-(4-hydroxyphenyl)prop-2-en-1-one. *Acta Crystallographica Section E* **2011**, *67* (4), o756.
(157) Wu, J.; Wang, C.; Cai, Y.; Peng, J.; Liang, D.; Zhao, Y.; Yang, S.; Li, X.; Wu, X.; Liang, G. Synthesis and crystal structure of chalcones as well as on cytotoxicity and antibacterial properties. *Medicinal Chemistry Research* **2012**, *21* (4), 444-452. DOI: 10.1007/s00044-011-9549-9.
(158) Carvalho, P. S.; Custodio, J. M. F.; Vaz, W. F.; Cirilo, C. C.; Cidade, A. F.; Aquino, G. L. B.; Campos, D. M. B.; Cravo, P.; Coelho, C. J.; Oliveira, S. S.; et al. Conformation analysis of a novel fluorinated chalcone. *Journal of Molecular Modeling* **2017**, *23* (3), 97. DOI: 10.1007/s00894-017-3245-8.
(159) Patil, P. S.; Teh, J. B.-J.; Fun, H.-K.; Babu, H. B. R.; Razak, I. A.; Dharmaprakash, S. M. 3-(3-Methoxyphenyl)-1-(4-methoxyphenyl)prop-2-en-1-one. *Acta Crystallographica Section E* **2007**, *63* (4), o1895-o1896.
(160) Teh, J. B.-J.; Patil, P. S.; Fun, H.-K.; Satheesh, Y. E.; Razak, I. A.; Dharmaprakash, S. M. (2E)-1-(3-Bromophenyl)-3-(4-chlorophenyl)prop-2-en-1-one. *Acta Crystallographica Section E* **2007**, *63* (4), o1844-o1845.
(161) Patil, P. S.; Chantrapromma, S.; Fun, H.-K.; Dharmaprakash, S. M. 1-(3-Bromophenyl)-3-[4-(dimethylamino)phenyl]prop-2-en-1-one. *Acta Crystallographica Section E* **2007**, *63* (4), o1738-o1740.
(162) Moulton, B. E.; Duhme-Klair, A. K.; Fairlamb, I. J. S.; Lynam, J. M.; Whitwood, A. C. A Rationale for the Linear Correlation of Aryl Substituent Effects in Iron(0) Tricarbonyl Complexes Containing α,β-Unsaturated Enone (Chalcone) Ligands. *Organometallics* **2007**, *26* (25), 6354-6365. DOI: 10.1021/om7006425.
(163) Lei, X.; Bai, X. (E)-1-(4-Chlorophenyl)-3-[4-(dimethylamino)phenyl]prop-2-en-1-one. *Acta Crystallographica Section E* **2009**, *65* (4), o739.
(164) Janardhana, K.; Ravindrachary, V.; Rajesh Kumar, P. C.; Yogisha; Ismayil. Third order nonlinear optical studies of 1-(4-chloro phenyl)-3-(4-dimethylamino phenyl) prop-2-en-1-one. *Journal of Crystal Growth* **2013**, *368*, 11-20. DOI: https://doi.org/10.1016/j.jcrysgro.2012.12.169.
(165) Hurst, D. P.; Titterington, J. A.; Van Wier, S. P.; Whitwood, A. C.; Wood, N. J. 1,3-bis(4-methoxyphenyl)prop-2-en-1-one. *CSD Communication (Private Communication)* **2018**, *1842226*.
(166) Qiu, X.-Y.; Liu, W.-S.; Zhu, H.-L. 3-(3-Hydroxyphenyl)-1-(4-nitrophenyl)prop-2-en-1-one. *Acta Crystallographica Section E* **2006**, *62* (4), o1304-o1305.
(167) Sarojini, B. K.; Yathirajan, H. S.; Mustafa, K.; Sarfraz, H.; Bolte, M. (2E)-1-(2-Hydroxyphenyl)-3-[4-(methylsulfanyl)phenyl]prop-2-en-1-one. *Acta Crystallographica Section E* **2007**, *63* (11), o4448.
(168) Fun, H.-K.; Jebas, S. R.; Razak, I. A.; Patil, P. S.; Dharmaprakash, S. M.; Deepak D'Silva, E. (E)-3-(2-Chlorophenyl)-1-(4-chlorophenyl)prop-2-en-1-one. *Acta Crystallographica Section E* **2008**, *64* (6), o1177.
(169) Shanthi, D.; Vidhyasagar, T.; Rajeswari, K.; Kayalvizhi, M.; Vasuki, G.; Thiruvalluvar, A. Crystal structure of (E)-1-([1,1'-biphenyl]-4-yl)-3-(3-nitrophenyl)prop-2-en-1-one. *Acta Crystallographica Section E* **2015**, *71* (2), o119-o120.
(170) Harini, K. S.; Quah, C. K.; Chidan Kumar, C. S.; Chandraju, S.; Lokanath, N. K.; Naveen, S.; Warad, I. (E)-1-(3-Bromophenyl)-3-(4-nitrophenyl)prop-2-en-1-one. *IUCrData* **2017**, *2* (2), x170287.
(171) Maragatham, G.; Selvarani, S.; Rajakumar, P.; Lakshmi, S. Crystal structures of three 1-[4-(4-bromobutoxy)phenyl] chalcone derivatives: (E)-1-[4-(4-bromobutoxy)phenyl]-3-phenylprop-2-en-1-one, (E)-1-[4-(4-bromobutoxy)phenyl]-3-(4-

methoxyphenyl)prop-2-en-1-one and (E)-1-[4-(4-bromobutoxy)phenyl]-3-(3,4-dimethoxyphenyl)prop-2-en-1-one. *Acta Crystallographica Section E* **2017**, *73* (8), 1232-1236.
(172) Salian, V. V.; Narayana, B.; Sarojini, B. K.; Mahesh, N.; Byrappa, K.; Kumar, S. M. Synthesis, crystal structures and Hirshfeld surface studies of chalcone derivatives: (2E)-1-(4-2, 4-Dichlorophenyl)-3-[4-(propan-2-yl)phenyl]prop-2-en-1-one and (2E)-1-(4-Fluorophenyl)-3-[4-(propan-2-yl) phenyl] prop-2-en-1-one. *Chemical Data Collections* **2018**, *15-16*, 54-61. DOI: https://doi.org/10.1016/j.cdc.2018.04.003.
(173) Sarojini, B. K.; Yathirajan, H. S.; Mustafa, K.; Sarfraz, H.; Bolte, M. (2E)-1-(2-Hydroxyphenyl)-3-(4-methoxyphenyl)prop-2-en-1-one. *Acta Crystallographica Section E* **2007**, *63* (11), o4477.
(174) Jasinski, J. P.; Butcher, R. J.; Narayana, B.; Lakshmana, K.; Yathirajan, H. S. (2E)-3-(4-Methylphenyl)-1-(3-nitrophenyl)prop-2-en-1-one. *Acta Crystallographica Section E* **2008**, *64* (1), o1-o2.
(175) Whitwood, A. C.; Pugh, D. S.; Wood, N. J.; Mills, A. L. F.; Aspell, A. M.; Coyne, E.; Hill, I. A.; Moore, O.; Latter, R. C. 3-(4-methylphenyl)-1-(3-nitrophenyl)prop-2-en-1-one. *CSD Communication (Private Communication)* **2019**, *1900359*.
(176) Shanthi, D.; Vidhya Sagar, T.; Kayalvizhi, M.; Vasuki, G.; Thiruvalluvar, A. (E)-1-([1,1'-Biphenyl]-4-yl)-3-(2-methylphenyl)prop-2-en-1-one. *Acta Crystallographica Section E* **2014**, *70* (7), o809-o810.
(177) Harshitha, K. R.; Sarojini, B. K.; Narayana, B.; G. Lobo, A.; Kumar, S. M.; Byrappa, K. Single crystal X-ray studies and Hirshfeld surface analysis of ethoxy phenyl substituted chalcone derivatives. *Chemical Data Collections* **2018**, *17-18*, 121-131. DOI: https://doi.org/10.1016/j.cdc.2018.08.002.
(178) Song, Z.; Kwok, R. T. K.; Zhao, E.; He, Z.; Hong, Y.; Lam, J. W. Y.; Liu, B.; Tang, B. Z. A Ratiometric Fluorescent Probe Based on ESIPT and AIE Processes for Alkaline Phosphatase Activity Assay and Visualization in Living Cells. *ACS Applied Materials & Interfaces* **2014**, *6* (19), 17245-17254. DOI: 10.1021/am505150d.
(179) Fun, H.-K.; Chia, T. S.; Nayak, P. S.; Narayana, B.; Sarojini, B. K. (2E)-3-(3-Nitrophenyl)-1-[4-(piperidin-1-yl)phenyl]prop-2-en-1-one. *Acta Crystallographica Section E* **2012**, *68* (4), o974.
(180) Patil, P. S.; Rosli, M. M.; Fun, H.-K.; Razak, I. A.; Puranik, V. G.; Dharmaprakash, S. M. 3-(3-Bromophenyl)-1-(4-methoxyphenyl)prop-2-en-1-one. *Acta Crystallographica Section E* **2006**, *62* (11), o4798-o4799.
(181) Harrison, W. T. A.; Yathirajan, H. S.; Narayana, B.; Mithun, A.; Sarojini, B. K. (2E)-1-(4-Chlorophenyl)-3-[4-(methylsulfanyl)phenyl]prop-2-en-1-one. *Acta Crystallographica Section E* **2006**, *62* (11), o5290-o5292.
(182) Ma, J.-L. (E)-3-(2-Methoxyphenyl)-1-(4-nitrophenyl)prop-2-en-1-one. *Acta Crystallographica Section E* **2007**, *63* (2), o806-o807.
(183) Ma, J.-L. (E)-3-(4-Methoxyphenyl)-1-(3-nitrophenyl)prop-2-en-1-one. *Acta Crystallographica Section E* **2007**, *63* (2), o808-o809.
(184) Patil, P. S.; Fun, H.-K.; Chantrapromma, S.; Dharmaprakash, S. M. 1-(4-Chlorophenyl)-3-(4-ethoxyphenyl)prop-2-en-1-one. *Acta Crystallographica Section E* **2007**, *63* (5), o2497-o2498.
(185) Sarojini, B. K.; Yathirajan, H. S.; Sreevidya, T. V.; Narayana, B.; Bolte, M. 3-(Biphenyl-4-yl)-1-(4-fluorophenyl)prop-2-en-1-one. *Acta Crystallographica Section E* **2007**, *63* (6), o2945.
(186) Custodio, J. M. F.; Faria, E. C. M.; Sallum, L. O.; Duarte, V. S.; Vaz, W. F.; de Aquino, G. L. B.; Carvalho, P. S.; Napolitano, H. B. The Influence of Methoxy and Ethoxy Groups on Supramolecular

Arrangement of Two Methoxy-chalcones <br clear="all" style="page-break-before:always;

mso-break-type:section-break" />. *Journal of the Brazilian Chemical Society* **2017**, *28* (11), 2180-2191. DOI: http://dx.doi.org/10.21577/0103-5053.20170067.
(187) Fun, H.-K.; Jebas, S. R.; Patil, P. S.; Dharmaprakash, S. M. (E)-3-(2-Chlorophenyl)-1-(3-methoxyphenyl)prop-2-en-1-one. *Acta Crystallographica Section E* **2008**, *64* (8), o1525.
(188) Jeyabharathi, A.; Ponnuswamy, M. N.; Nanjundan, S.; Fun, H.-K.; Chantrapromma, S.; Usman, A.; Razak, I. A. 4-[3-(3,4-Dimethoxyphenyl)prop-2-enoyl]phenyl methacrylate and 4-[3-(2-bromophenyl)prop-2-enoyl]phenyl methacrylate. *Acta Crystallographica Section C* **2002**, *58* (1), o26-o28.
(189) Zhao, P.-S.; Wang, X.; Guo, H.-M.; Jian, F.-F. 3-(4-Fluorophenyl)-1-(4-methoxyphenyl)prop-2-en-1-one. *Acta Crystallographica Section E* **2009**, *65* (6), o1402.
(190) Hasan, A.; Akhtar, N.; Lajis, N. H.; Rusli, A. F. B.; Lo, K. M. (3E)-3-[4-(Dimethylamino)phenyl]-1-(4-hydroxyphenyl)prop-2-en-1-one. *Acta Crystallographica Section E* **2010**, *66* (7), o1808.
(191) Yamuna, T. S.; Yathirajan, H. S.; Jasinski, J. P.; Keeley, A. C.; Narayana, B.; Sarojini, B. K. (2E)-1-(4-Chlorophenyl)-3-(4-nitrophenyl)prop-2-en-1-one. *Acta Crystallographica Section E* **2013**, *69* (5), o790-o791.

(192) Sugden, I.; Adjiman, C. S.; Pantelides, C. C. Accurate and efficient representation of intramolecular energy in ab initio generation of crystal structures. I. Adaptive local approximate models. *Acta Crystallographica Section B-Structural Science Crystal Engineering and Materials* **2016**, *72*, 864-874, Article. DOI: 10.1107/S2052520616015122.
(193) Kazantsev, A. V.; Karamertzanis, P. G.; Adjiman, C. S.; Pantelides, C. C. CrystalOptimizer. An efficient Algorithm for Lattice Energy Minimisation of Organic Crystal using Isolated-Molecule Quantum Mechanical Calculations. In *Molecular System Engineering*, Adjiman, C. S., Galindo, A. Eds.; Process Systems Engineering, Vol. 6; WILEY-VCH Verlag GmbH & Co., 2010; pp 1-42.
(194) Price, S. L.; Leslie, M.; Welch, G. W. A.; Habgood, M.; Price, L. S.; Karamertzanis, P. G.; Day, G. M. Modelling Organic Crystal Structures using Distributed Multipole and Polarizability-Based Model Intermolecular Potentials. *Physical Chemistry Chemical Physics* **2010**, *12* (30), 8478-8490.
(195) Stone, A. J. GDMA: A Program for Performing Distributed Multipole Analysis of Wave Functions Calculated Using the Gaussian Program System. *GDMA2.2* **2010**.
(196) Williams, D. E.; Cox, S. R. Nonbonded Potentials For Azahydrocarbons: the Importance of the Coulombic Interaction. *Acta Crystallographica Section B - Structural Science* **1984**, *40* (8), 404-417. Cox, S. R.; Hsu, L. Y.; Williams, D. E. Nonbonded Potential Function Models for Crystalline Oxohydrocarbons. *Acta Crystallographica Section A - Crystal Physics, Diffraction, Theoretical and General Crystallography* **1981**, *37* (MAY), 293-301.
(197) Pidcock, E.; Motherwell, W. D. S. A new model of crystal packing. *Chemical Communications* **2003**, (24), 3028-3029.
(198) Hansch, C.; Leo, A.; Taft, R. W. A Survey of Hammett Substituent Constants and Resonance and Field Parameters. *Chemical Reviews* **1991**, *91* (2), 165-195. DOI: 10.1021/cr00002a004.
